# Differential genomic arrangements in Caryophyllales through deep transcriptome sequencing of *A*. *hypochondriacus*

**DOI:** 10.1371/journal.pone.0180528

**Published:** 2017-08-07

**Authors:** Meeta Sunil, Nivedita Hariharan, Shubham Dixit, Bibha Choudhary, Subhashini Srinivasan

**Affiliations:** 1 Institute of Bioinformatics and Applied Biotechnology, Bangalore, Karnataka, India; 2 Manipal University, Manipal, Karnataka, India; University of Western Sydney, AUSTRALIA

## Abstract

Genome duplication event in edible dicots under the orders Rosid and Asterid, common during the oligocene period, is missing for species under the order Caryophyllales. Despite this, grain amaranths not only survived this period but display many desirable traits missing in species under rosids and asterids. For example, grain amaranths display traits like C4 photosynthesis, high-lysine seeds, high-yield, drought resistance, tolerance to infection and resilience to stress. It is, therefore, of interest to look for minor genome rearrangements with potential functional implications that are unique to grain amaranths. Here, by deep sequencing and assembly of 16 transcriptomes (86.8 billion bases) we have interrogated differential genome rearrangement unique to *Amaranthus hypochondriacus* with potential links to these phenotypes. We have predicted 125,581 non-redundant transcripts including 44,529 protein coding transcripts identified based on homology to known proteins and 13,529 predicted as novel/amaranth specific coding transcripts. Of the protein coding *de novo* assembled transcripts, we have identified 1810 chimeric transcripts. More than 30% and 19% of the gene pairs within the chimeric transcripts are found within the same loci in the genomes of *A*. *hypochondriacus* and *Beta vulgaris* respectively and are considered real positives. Interestingly, one of the chimeric transcripts comprises two important genes, namely DHDPS1, a key enzyme implicated in the biosynthesis of lysine, and alpha-glucosidase, an enzyme involved in sucrose catabolism, in close proximity to each other separated by a distance of 612 bases in the genome of *A*. *hypochondriacus* in a convergent configuration. We have experimentally validated that transcripts of these two genes are also overlapping in the 3’ UTR with their expression negatively correlated from bud to mature seed, suggesting a potential link between the high seed lysine trait and unique genome organization.

## Introduction

*Amaranthus hypochondriacus* (Linn.) is a diploid plant species (2n = 32) belonging to the dicot family Amaranthaceae classified under the order Caryophyllales. *A*. *hypochondriacus* (Ah) and the other grain amaranths (*A*. *caudatus* and *A*. *cruentus*) are known to possess many desirable traits including unique nutritional profile, high seed lysine, high seed yield, drought resistance, pest tolerance and high biomass [1,2], which are rare in other edible dicots. Grain amaranths are also of great interest as they undergo C4 photosynthesis for carbon fixation also rare among edible dicots. Also grain amaranths use betalains, a class of accessory pigments in plants unique to species under Caryophyllales, for coloration possessing high antioxidant properties [3]. All these agronomically desirable traits displayed by grain amaranth is stimulating further research to link these phenotypes to the respective genotypes.

The genomes of majority of the plants of economic interest has now been sequenced. This has already revealed that genome duplication has been used by plant species to survive catastrophic environmental changes during the cretaceous and oligocene periods [4]. More recently, sequencing the genomes of grain amaranths and *Beta vulgaris* (Bv), the first few species to be sequenced under Caryophyllales, reveal that species under Caryophyllales may not have gone through genome duplication during the oligocene period common among other edible dicots [5,6]. It is of interest to know how the species under Caryophyllales survived this period and still display many desirable traits missing from other edible dicots.

Small-scale genome reorganization (overlapping genes) forms the second level of gene regulation in plants [7] and is responsible for diversity in traits displayed by the species. Overlapping genes in prokaryotic genomes have been well known. They are seen commonly in viruses, mitochondria, and bacteria, and are suggested to compose a compact genome organization to facilitate gene regulation efficiency like operons [8,9]. Recently, reports from eukaryotic genomes have emerged. Overlapping genes resulting in chimeric transcripts, especially readthrough transcripts are now well documented for animals, especially in human diseases such as cancer [10–12]. However, in plants, few reports have started emerging on transcription induced chimera (TICs) and natural antisense transcripts (NATs). Of these studies, majority are from *Arabidopsis thaliana*, which is a model organism for plant studies. Almost 11% of *A*. *thaliana* genes are reported to be overlapping (in 5’ or 3’ ends) with their NATs that may provide a substrate for RNAi activity through the formation of nat-siRNAs. Another 28% of sense-antisense overlapping gene pairs are regulated in tissue-specific manner [13,14].

Clusters of genes forming an operon-like regulon are also identified in plants. For example, Bx genes TaBx-1-5, implicated in the synthesis of secondary metabolite Benzoxazinones, has been reported to form a single cluster in *Zea mays* genome [7]. These genes are found split into two groups that are not in the same chromosomes in the genomes of Rye, barley and wheat, perhaps from divergent genome reorganization. Similarly, genes implicated in avenacin biosynthetic pathways are also found clustered in *Avena sativa*. In this case, it is believed that one of the homologous gene landed within a non-homologous gene cluster belonging to this pathway by way of gene duplication [15].

More recently, because of NGS technologies, many more plant genomes and transcriptomes have been sequenced and reported providing the much needed resource to study differential genome organization. While most transcriptome efforts report chimeric transcripts from assembly artefacts, these reports have not yet been utilized to study differential genome reorganization with potential functional consequences. For example, a report on transcriptome assembly of *Ricinus communis* elaborates more on the assembly methods that result in least number of false chimeras [16]. Here, by deep sequencing of developmental transcriptomes of Ah across different tissues we have identified and characterized in Ah and reveal genome organization that is unique to Caryophyllales with potential functional consequences.

## Results and discussion

From the 84.8 billion bases of high quality transcriptome sequencing, we predict 1,25,581 non-redundant transcripts (Table 1). The 1,25,581 predicted transcripts correspond to 88,999,253 bases which is equivalent to 19% of the 466 Mb genome getting transcribed. Of these, 58058 are protein coding transcripts based on both orthology (44,529) to all known proteins and gene prediction algorithms (13,529). The rest, 67,523 are classified as non-coding transcripts. The 58,058 predicted coding transcripts correspond to 50,229,140 bases which is equivalent to 10.76% of the genome. The completeness of the proteome has been validated by aligning predicted proteome to the 248 core eukaryotic orthologous genes (CEGMA KOGs) with a coverage of >70%.

**Table 1:** Assembly metrics of the merged transcriptome.

**Total number of transcripts**	1,25,581
**Longest transcript (bases)**	16,560
**Average transcript length (bases)**	709
**N50 (bases)**	834
**Total assembly size (bases)**	88,999,254
**GC %**	38.61

**Fig 1:**
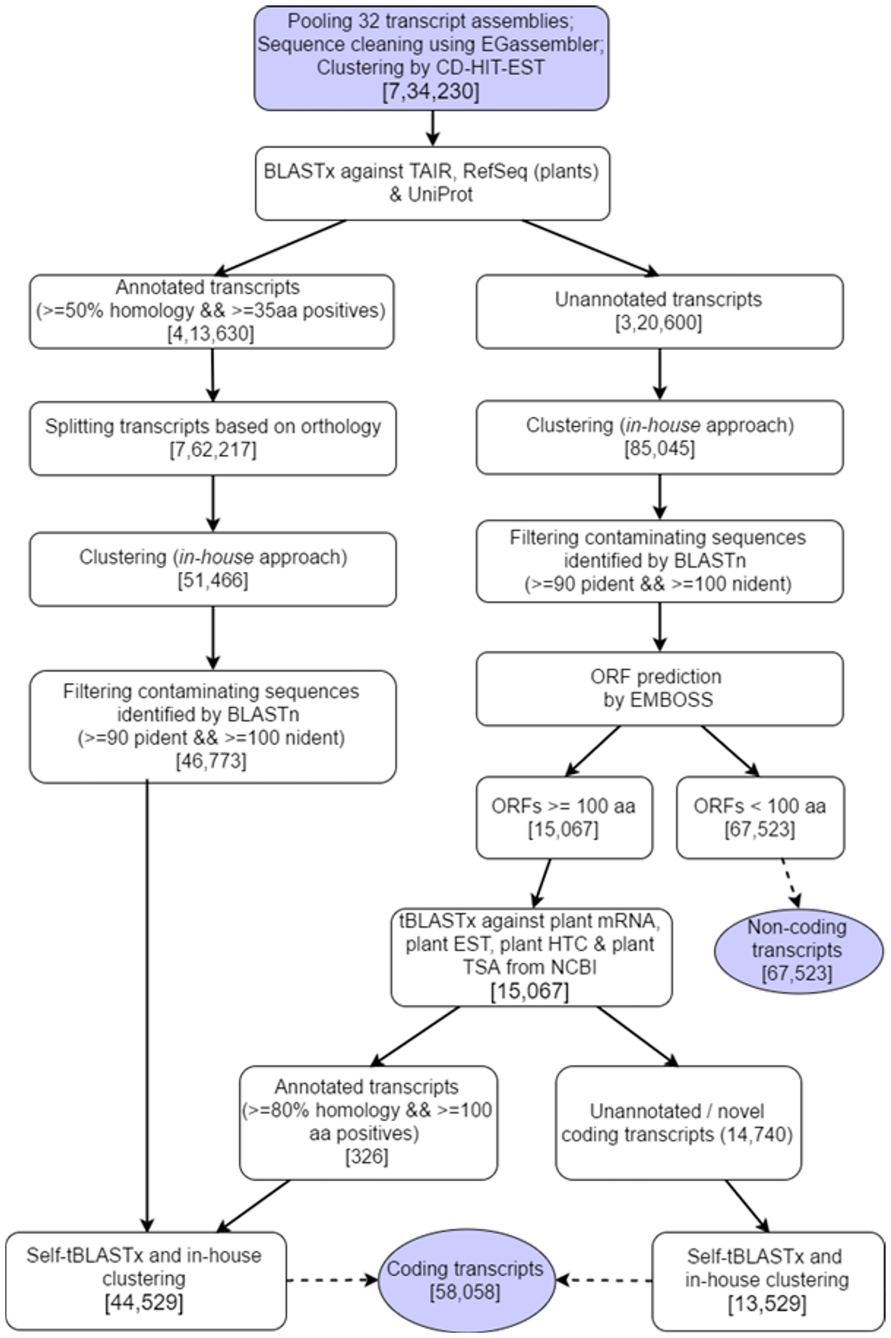
Flow chart showing the approach for generating non-redundant set of transcripts from multiple assemblies.

**Fig 2:**
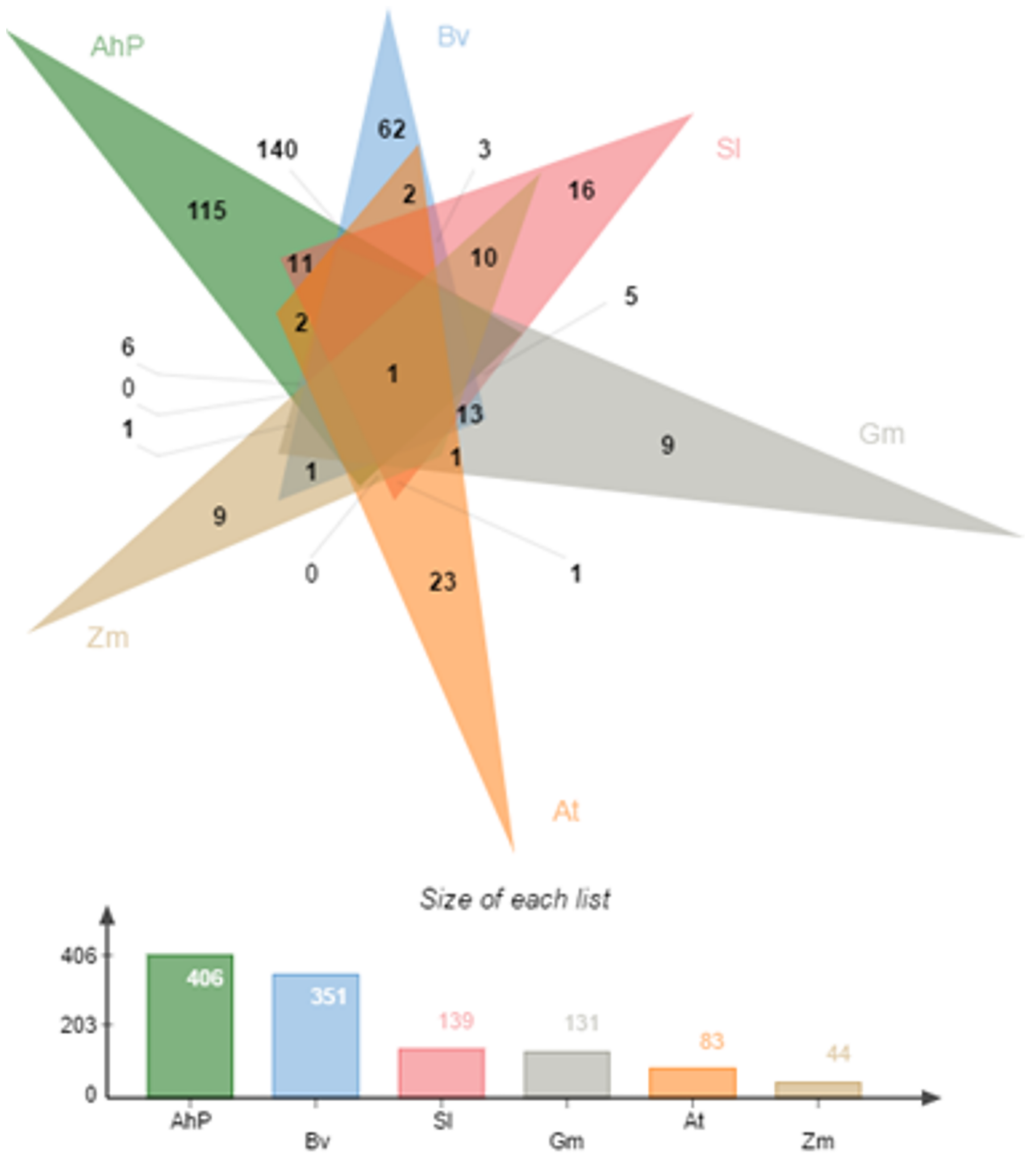
Venn diagram showing the number of chimeric transcripts validated in plant genomes (AhP: *Amaranthus hypochondriacus*; Bv: *Beta vulgaris*; Sl: *Solanum hypochondriacus*; Gm: *Glycine max*; At: *Arabidopsis thaliana*; Zm: *Zea mays*).

A total of 1810 chimeric transcripts were identified in the transcript assembly. From these, we removed large number of false positives stemming from transcript assembly tools and automation of the annotation pipeline (shown in Fig 1) owing to incomplete annotation or variation in annotation of same proteins across different databases and species. Among the 1810 chimeric transcripts (S1 Dataset), there are 581 that are likely to be real positives as the gene pairs forming the chimera are found within the same loci in plant genomes compared here (S2 Dataset). Of the 581, 406 (70%) are found in the same loci on Ah genome, 351 (60%) in Bv genome (Caryophyllales), 139 (24%) in Sl genome (Asterid), 131 (20%) in Gm, 83 (14%) in At (Rosid), and 44 (7%) in Zm (monocot) as shown in Fig 2 and Table 2. As expected, 32% (581) of the 1810 initial chimeric transcripts turned out to be real positives and are validated in dicots. The loci of the genes comprising one chimera is found to be conserved across the plant kingdom, which comprises of genes Phosphoglycerate mutase family protein (AT1G22170) and Sec14p-like phosphatidylinositol transfer family protein (AT1G22180). Gene pairs of 8 other chimeric transcripts have shared loci in all dicot genomes compared here with 140 chimeric transcripts specific to Caryophyllales and 115 specific to amaranth. A Gene Ontology enrichment analyses of these chimeric genes revealed that apart from being chloroplastic or mitochondrial in origin, many genes are nuclear and are involved in integral biological processes like transport, developmental processes, stress-response and signal transduction. For example, as shown in S1 Fig, 6.1% are enriched for developmental processes, 5.6% for response to abiotic and biotic stimulus, 5.6% for protein metabolism and 5.2% for transport. Also, Caryophyllales-specific genes are from diverse cellular compartments including 13.7% chloroplastic, 8.2% nuclear, 7% plastid, 6% mitochondrial, and 5.5% in plasma membrane.

**Table 2:** Count of chimeric transcripts validated across plant genomes.

	Ah	Bv	At	Gm	Sl	Zm	Ah-specific	Caryo-specific
# chimera	406	351	83	131	139	44	110	132
# multi-gene	4	2	0	1	12	10	0	1
**# pairs**	**404**	**349**	**83**	**140**	**130**	**40**	**110**	**131**
Unique	402	349	83	140	127	34	109	131
Duplicated	2	0	0	0	3	6	1	2
**# overlapping**	**30**	**6**	**25**	**11**	**11**	**7**	**4**	**15**
Convergent	28	5	25	11	10	2	4	13
Divergent	2	0	0	0	0	1	0	2
Co-oriented	0	1	0	0	1	4	0	0
**# contained**	**0**	**1**	**1**	**5**	**0**	**5**	**0**	**0**
Convergent	0	0	0	0	0	0	0	0
Divergent	0	0	0	0	0	0	0	0
Co-oriented	0	1	1	5	0	5	0	0
**# non-overlapping**	**374**	**342**	**57**	**124**	**119**	**28**	**106**	**116**
Convergent	302	276	29	94	67	3	78	102
Divergent	6	9	4	1	3	5	2	1
Co-oriented	66	57	24	29	49	20	26	13

It is not surprising that many chimeric transcripts are coming from chloroplasts and mitochondria which tend to form operon-like structures, as has been reported in other plant species [17]. Among the dicot-specific chimera of nuclear origin, we have identified a chimeric transcript comprising 3 genes for SAUR-like auxin-responsive proteins. These genes are quite homologous at the protein level and are present in different configurations in different plant genomes compared here (S2 Fig). In Ah genome, the same gene locus is the best hit for all the three SAUR genes on scaffolds 1147 and 2872, but on scaffold 401, only the first two genes hit to identical coordinates and the third gene is in divergent configuration with them at a distance of 3.7 Kb. In Bv genome, the second and third genes hit to the identical coordinates on the genome and are in divergent configuration with the first gene at a distance of 11.3 Kb. In At, genes 2 (AT1G29490) and 1 (AT1G29510) are in a co-oriented configuration with an interval of 2.1 Kb. In Gm, genes 2 and 1 hit to identical coordinates on the genome and are in co-oriented configuration with gene 3 at a distance of 2.1 Kb. In Sl, the chimera is validated on two chromosomes. While chromosome 4 has genes 1 and 3 in co-orientation at a distance of 2.1 Kb and genes 3 and 2 in convergence at a distance of 10.3 Kb, chromosome 11 has genes 2 and 3 hitting to identical coordinates and being co-oriented to gene 1 at a distance of 6.3 Kb. Interestingly, these three genes are also found to be tightly co-expressed in Ah with the expression being suppressed in the root and spike up in the bud stage. (S3, S4 & S5 Datasets, Transcript 1245). In Gm, SAUR genes have been reported [18] to form a cluster comprising five homologous genes, spaced at intervals of about 1.25 kilobases and transcribed in alternate directions. In this chimeric transcript, we identify two of the five genes from the cluster in Gm.

Table 2 shows the orientations of the genes within the 581 chimeric transcripts validated in several genomes compared here. For example, the genes within the 406 chimeric transcripts that share the same gene loci on the Ah genome, are classified into 402 paired and 4 multi-gene chimera. Among the 402 chimeric pairs, 2 pairs (‘duplicated’) are found to be present in more than one copy on the Ah genome, thus adding up to 404 chimeric pairs (with 400 unique pairs and two copies of two pairs). Of these 404 chimeric pairs, 374 have their gene mates separated on the genome by a distance up to 50 Kb and are referred to as ‘non-overlapping’ pairs. In other 30 pairs, the coordinates of one gene partially overlaps in the coding region with the coordinates of the other gene. Such pairs are referred to as ‘overlapping’ throughout this manuscript. Those pairs where the coding part of one gene completely falls within the coordinates of the other gene, these are termed as ‘contained’ throughout this manuscript, which are very few in number.

The chimeric pairs are further classified based on their orientations in the genome as convergent (with 3’ sharing), divergent (with 5’ sharing) and co-oriented (with same orientation in either 3’-5’ or 5’3’ direction). It is observed that the gene pairs where one gene is contained in the coordinates of the other, are always co-oriented. Also, the number of convergent gene pairs predominates while the divergent pairs are few. For example, 28 out of 30 overlapping pairs and 302 of 374 non-overlapping pairs are convergent in configuration while only 2 of the 30 and 6 of the 374 are divergent. While there are no co-oriented configurations under the overlapping category, there are 66 in the non-overlapping category. This is also consistent with the fact that overlapping genes are more likely to be coded on the opposite strands of the genome.

**Fig 3:**
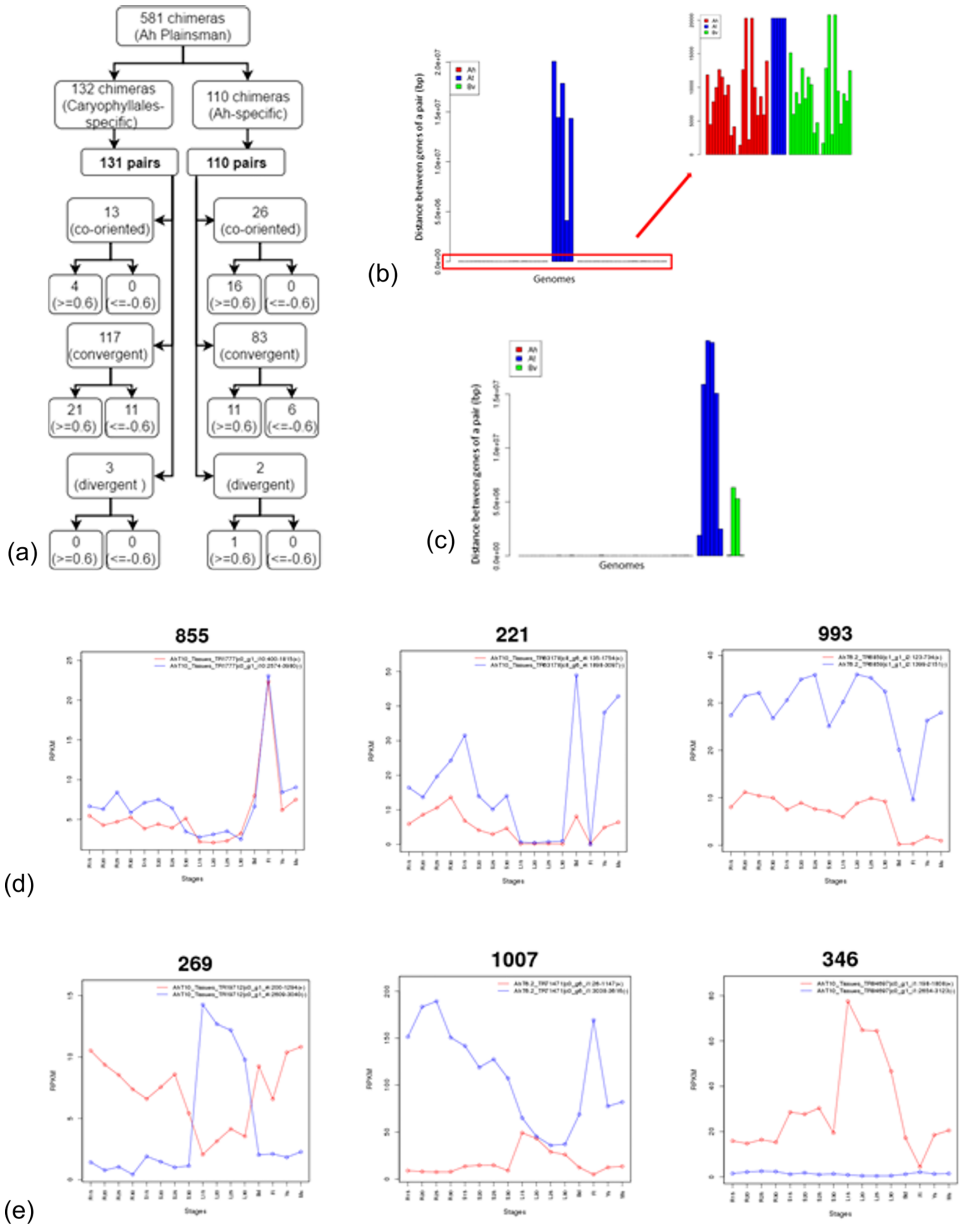
Caryophyllales-specific and amaranth-specific chimeras. Count of different types of chimeras (a), length distribution of caryophyllales-specific (b) and amaranth-specific (c) chimeras, and expression profiles of three positively correlated (d) and three negatively correlated (e) amaranth-specific chimeras.

Fig 3(a) lists the number of chimeric genes with the expression of their individual genes coregulated or anti-regulated. Among the 13 co-oriented Caryophyllales-specific chimeric gene pairs, 4 pairs are positively correlated (with r > = 0.6) in expression across the 16 conditions sequenced (with 4 developmental stages across 4 tissues) and none are negatively correlated. Out of the 117 convergent configurations under the Caryophyllales-specific chimeras, the expression profiles of 21 pairs are positively correlated and 11 pairs are with negative correlation (with r < = -0.6). The 3 chimeric pairs in divergent configuration show no correlation in expression profile. These observations are consistent with those reported for *A*. *thaliana*. i.e. co-oriented gene pairs are more tightly coregulated than sense-antisense gene pairs. Also, it has been reported that adjacent/overlapping gene pairs in a genome are more likely to be tightly co-regulated than unpaired genes [19].

**Fig 4:**
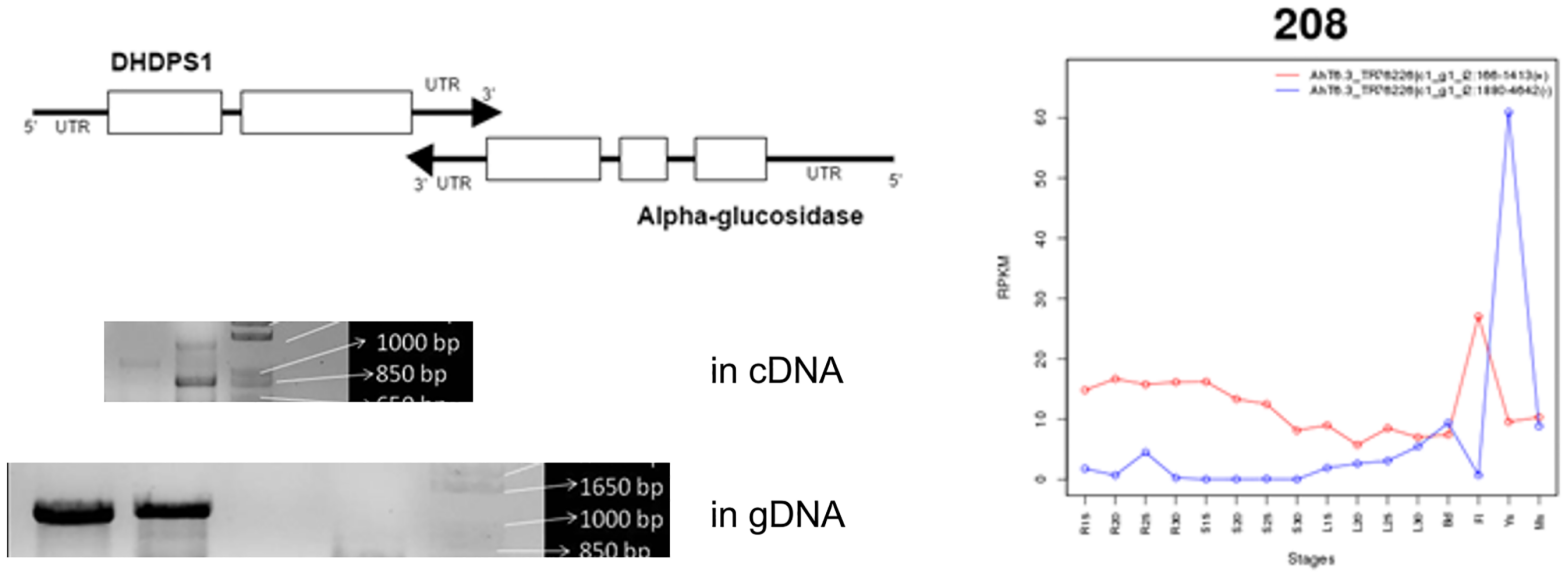
DHDPS1-alpha glucosidase overlap in *A*. *hypochondriacus*. Schematic diagram showing the convergent DHDPS1 and alpha-glucosidase pair with overlapping 3’ UTRs (top left), PCR validation of DHDPS overlap from cDNA and gDNA showing the expected bands of 1.2 Kb for the chimeric product containing the overlapping 3’ UTR region (bottom left) using the primers shown in S3 Fig, and expression profiles of the two transcripts (right) across the 16 tissue and developmental stages sequenced.

Among the Caryophyllales-specific chimeric transcripts we find a gene pair that may have functional consequences on the most important phenotype of Ah, such as high-lysine seeds. One of the chimeric transcripts includes the gene for DHDPS1 and alpha-glucosidase. DHDPS (EC 4.3.3.7) is one of the key enzymes regulating lysine synthesis while alpha-glucosidase (EC 3.2.1.20) is involved in starch and sucrose catabolism. These two genes are separated by a very short distance in the Ah genome and found to be in a convergent configuration (Fig 4). From an in-silico analysis, we observe linkage between DHDPS1 and alpha-glucosidase in Ah and Bv where their coding sequences are only 612 bp and 11.3 Kbp apart, respectively. The expression profiles of DHDPS1 and alpha-glucosidase are shown in Fig 4(c). Alpha-glucosidase is involved in the breakdown of D-sucrose while DHDPS1 is involved in the building up of L-lysine and they are both negatively correlated with one being active while the other is suppressed. Thus, the unique proximity of these two genes in both Ah and Bv (Caryophyllales-specific gene pairs) is very interesting from the nutritional point of view. We validated the distance between the two genes, DHDPS1 and alpha glucosidase in both the cDNA and gDNA from *A*. *hypochondriacus* using multiple primer pairs spanning the 612 bases separating the two genes. Our result validates their proximity on the Ah genome and suggests that the transcripts of the two genes overlap in the 3’ regions, which explains the chimeric nature of the transcripts from transcriptome assembly tools that ignore strand orientation.

**Fig 5:**
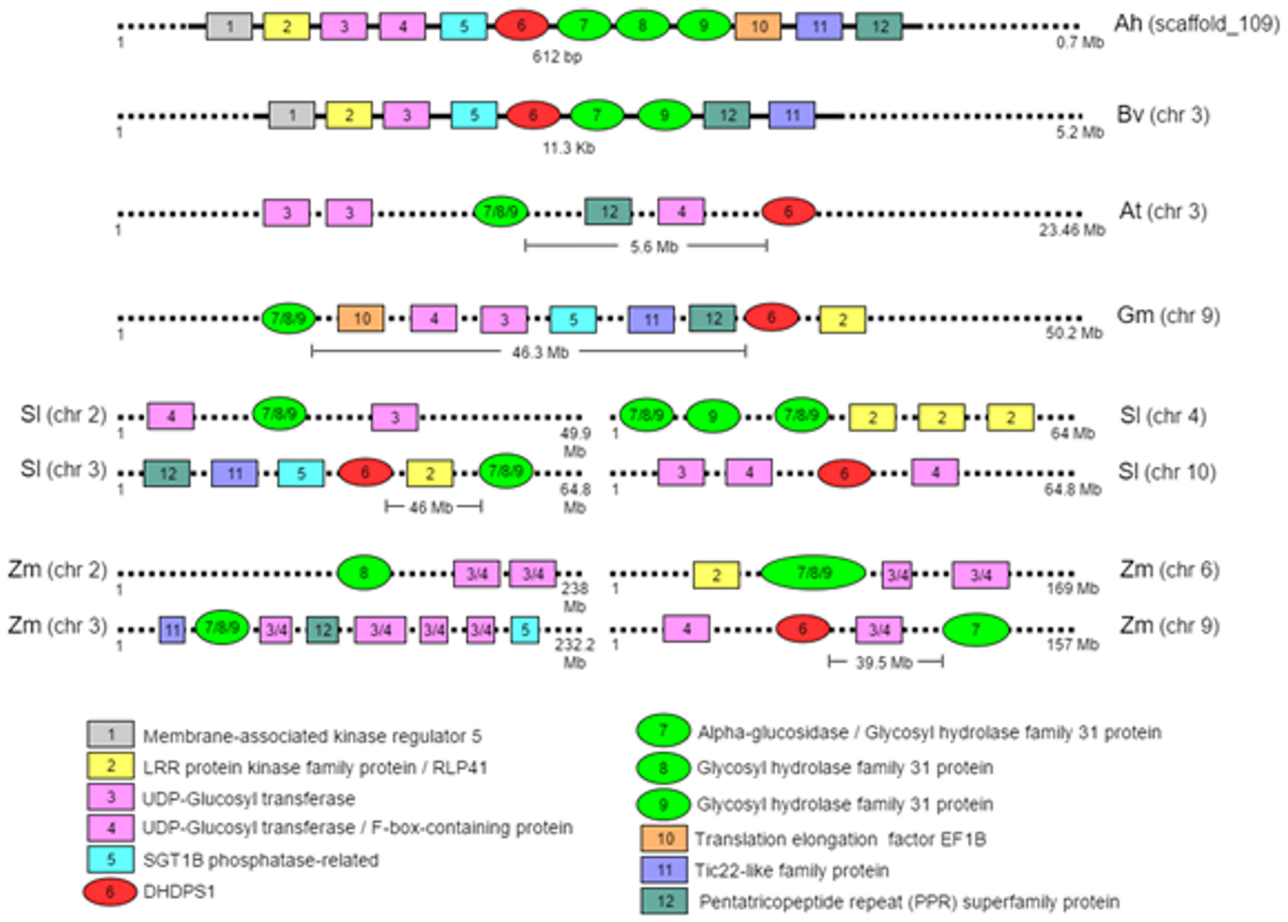
Microsynteny in Ah (*Amaranthus hypochondriacus*) and Bv (*Beta vulgaris*) in the region containing DHDPS1 and alpha glucosidase as compared to the diffused arrangement of the genes in At (*Arabidopsis thaliana*), Gm (*Glycine max*), Sl (*Solanum lycopersicum*) and Zm (*Zea mays*).

DHDPS1 and alpha-glucosidase are neighbouring genes in opposite strands in Ah and Bv whereas they are either far apart or on different chromosomes altogether in other dicots and monocots compared here (Fig 5). In Ah these two genes are separated on the genome by 612 bases and in Bv it is separated by 11.3 kilobases. The distance between these two genes in Gm is 46 million and at in At 5.5 million bases with two intervening genes from the same block of colinear genes in Ah suggesting gene shuffling. In Sl and Zm one of the DHDPS isoenzyme is found closer to one of the glucosidase genes in chromosomes 3 separated by 46 million bases and chr9 separated by 39.5 million bases respectively. This suggests that the two genes may be linked in Caryophyllales which may be a recent convergence evolution in Caryophyllales or divergent evolution in other species triggered by genome duplication event missed by Caryophyllales. The crowding of the 3’ UTR of two genes in the convergent configuration from the opposite strands may suggest cooperative regulation during energy-exhausting seed development.

The high-throughput and low cost of DNA sequencing has enabled us to interrogate the genomes and transcriptomes of many organisms including even the non-agronomic or non-model species. In non-agronomic crops, one does not have the luxury of having thousands of variant plants with variant phenotypes for SNP-based genotyping. In order to explore the genotype for the many desirable traits in Ah we have looked into the chimeric transcripts from transcriptome assembly for clues to differential genome organization across species that can shed light on the differential mechanisms. Since transcriptome sequencing is both essential and cheaper to decipher the biology behind various phenotypes, we have used deep transcriptome assembly to study differential genome organization that may have resulted in differential phenotype. Using the chimeric transcripts from massive assembly of 84.8 billion bases of transcriptome sequencing of 4 tissues across 4 developmental stages, we have identified 406 Caryophyllales-specific functionally important gene pairs that are too close in the genome to be functionally irrelevant. For one such gene pair involving DHDPS1, a critical gene is lysine biosynthetic pathway, and a-glucosidase separated in the genome by as small as 612 bases, we have experimentally validated that the transcripts overlap in the 3’ UTR with potential role in co-regulation and stabilization of RNA. We believe that this study is the first in utilizing chimeric transcripts from transcriptome assembly to study differential genome organization of functionally critical genes that may relate to phenotypes.

## Materials and methods

### Sample collection

Seeds of Ah stored and maintained at IBAB as previously reported [6], were grown for four generations in isolation in the institute premises on natural red soil of Karnataka. Tissues from individuals from two batches of third generation plants and one batch of fourth generation plants were used for transcriptome sequencing. Leaf, stem and root tissues were collected from a pool of more than 10 individuals from the three batches separately at (i) 15 days, (ii) 20 days, (iii) 25 days and (iv) 30 days of age, along with inflorescence including (v) buds, (vi) flowers, (vii) young seeds and (viii) mature seeds for RNA extraction. The tissues, once excised, were immediately cleaned with RNAse-free water, snap frozen in liquid nitrogen and further stored at -80°C until RNA extraction was done. The mature seeds were collected from the inflorescences which were sun-dried, threshed, cleaned and then stored at -80°C. Thus, 48 tissue samples were collected and frozen.

### Extraction of total RNA

Total RNA was extracted from the 48 tissues using the conventional Trizol extraction method [20] as standardized in the laboratory. However, during the homogenization step itself, the three stem and root samples (corresponding to cDNA libraries 6 and 7 in S1 Table) from 15 days old plants were each pooled together due to limitation in the amount of tissue available unlike the other samples where pooling was done after RNA extraction.

### Library preparation and quantification

One microgram of the total RNA extracted from the same type of tissue but from the three different batches of the plants were pooled together to make 3μg of the starting material for library preparation as measured by the NanoDrop Spectrophotometer. Thus, 14 pools of RNA samples were made in addition to the 2 pools (pools 2 and 3 in S1 Table) that were made by mixing equal amounts of the tissues during RNA extraction. Further, transcriptome libraries were prepared using TruSeq RNA Sample Preparation Low Throughput (LT) protocol (Illumina) as per the manufacturer’s guidelines and as reported previously [6]. The quality and quantity of the libraries were estimated by Qubit fluorometry (Invitrogen), and the size distribution was analysed on Bioanalyzer (Agilent) using high sensitivity DNA chips.

### Sequencing

The 16 barcoded cDNA libraries (S1 Table) were multiplexed using 4.5 pM of each and seeded onto three lanes of the flow cell. Cluster generation was done on cBot (Illumina) and paired-end sequencing on Hiseq 2500 (Illumina) to generate 100 bp long reads following the manufacturer’s recommendations.

### Reads QC

The sequenced reads were demultiplexed and QCed using Casava 1.8.2 pipeline (Illumina), in-house scripts and FastQC [21]. All the read pairs having at least 75% of the bases with Phred quality 20 or more and at the most 15 Ns were considered to be of good quality and were used for further analyses.

### *De novo* assembly and quality control

#### Assembly using transcriptome *de novo* assemblers

A total of 20 transcriptomes (as listed in S1 Table) were sequenced in-house and used for this study. Out of these, 16 were sequenced afresh while 4 had been sequenced and assembled previously [6]. Multiple assemblies were run using Oases v0.2.8 [22], SOAPdenovo-Trans v1.03 [23], Trinity r20130216 and Trinity 2.0.5 [24] using a combination of different read pools from the 20 transcriptomes (as listed in S2 Table) and merged for annotation.

#### Merging *de novo* assemblies

The pooled transcriptome for downstream analyses was achieved by merging all the assemblies together first using CD-HIT-EST [25] with -c 1.0 parameter followed by sequence cleaning using the EGassembler [26] pipeline with default parameters, while disabling organelle masking process, and clustering again by CD-HIT-EST with -c 0.99 to remove redundant transcripts that may differ slightly due to sequencing error or mis-assembly. Thus, from a pool of 1.2 million transcripts, 0.7 million transcripts were obtained after clustering with 99% identity.

### Creating non-redundant sets of coding and non-coding transcripts

#### Prediction of coding and non-coding transcripts

The 0.7 million redundant set of transcripts were aligned to known proteins from multiple public repositories for annotation. BLASTX [27] of the transcripts was done against TAIR10, RefSeq (extracted for Viridiplantae) and UniProt databases. The results were parsed to calculate the total number of positives and identities per query-subject pair and further filtered with homology > = 50%, number of positives > = 35 amino acids and evalue < = 1e-05. Out of the 0.7 million transcripts for *A*. *hypochondriacus*, 0.4 million got annotated to coding genes. Some of these transcripts were found to be chimeras which could be assembly artefacts or true biological chimeric transcripts. Therefore, the 0.4 million transcripts that could hit to orthologous proteins were split into 0.7 million transcripts based on alignment coordinates of the orthologous regions extended by 75 bases on each end. These 0.7 million transcripts were further clustered into a non-redundant set of coding transcripts based on a novel clustering approach as described below. The filtering, splitting and clustering steps were done using *in-house* scripts and MySQL tables.

#### Clustering of transcripts into non-redundant sets

A novel *in-house* method for clustering together redundant transcripts from multiple *de novo* assemblies and isoforms of a gene, without alignment to the reference genome, was developed and utilized to create non-redundant sets of coding annotated, coding novel and long non-coding transcripts. The clustering is based on a greedy algorithm that implements the logic that if two *de novo* assembled transcript sequences are > = 95% identical across a stretch of 100 or more identical bases, then they are essentially the same sequences with slight variations owing to sequencing/assembly errors or being isoforms. Therefore, before clustering, a self-BLASTN is done and the hits that pass the given filters are processed by a clustering program written in C++ that will assign clusters greedily to all the transcripts, i.e., if a transcript has been assigned a cluster, all the other transcripts that hit to the cluster-assigned transcript, or any other transcript in the same cluster, will be assigned the same cluster number.

Multiple levels of filtering are essential prior to clustering to minimize merging of distinct clusters due to sequencing, assembly or annotation errors and to capture maximum number of unigenes or isoenzymes. These filters include (i) retaining the longest self-BLASTN alignment block in cases where there are multiple alignments for the same query-subject pair that can occur due to mis-assembly especially with shorter k-mers, (ii) removing transcripts from 21-mer assemblies to avoid merging of otherwise distinct clusters due to representatives of misassembled transcripts in both the sets, (iii) optionally, removing all the transcripts shorter than 300 bases, and (iv) retaining only those self-BLASTN hits that have > = 95% identity across > = 100 nt identical bases, including the self-hits to ensure that every transcript in the set is assigned a cluster.

The clustering method was validated by manually checking for all the unigenes from lysine biosynthesis pathway in the non-redundant coding annotated set. All the isoenzymes implicated in the pathway could be identified in the clustered set. Whereas, many unigenes from the pathway were missing in multiple other clustering attempts made using CD-HIT-EST, especially isoenzyme 1 of dihydrodipicolinate synthase (DHDPS) which is a key regulatory enzyme of the lysine biosynthesis pathway.

#### Screening for human and microbial contamination

The transcript sequences were aligned (BLASTN) against the human (hg19) and microbial (downloaded from NCBI) reference genomes to identify sequences of microbial origin that may come from contamination from the field or from endophytes within the plant tissues. The sequences with percent identity > = 90 and > = 100 positives were considered to be of human/microbial origin and were filtered out from the transcript pool.

A further self-tBLASTx of the predicted coding (orthologous and novel) transcripts was done using the same filters of 90% identity and 100 identities to further reduce some redundancy at protein level.

The entire workflow is depicted in Fig 1.

### Prediction of novel coding transcripts

ORF prediction was done on the transcripts that could not be annotated based on orthology using getorf program from EMBOSS [28] package and selecting for the longest ORF using in-house script.

### Validation of transcript sets

The conventional assembly metrics of the transcripts were calculated using in-house scripts. The completeness of the set of coding transcripts was validated by performing tBLASTn of the set of 248 core eukaryotic genes (CEGMA) against the transcripts.

### Transcript expression and its normalization

The high quality 100 bp Illumina reads were mapped to the *de novo* assembled transcriptome using Bowtie2 [29] and the read counts calculated using SAMtools [30] and BEDTools [31]. Transcript abundance was measured using the conventional RPKM method.

### Identification of gene chimera from transcriptome assembly

Chimeras among the coding transcripts were identified from the BLASTx performed for prediction of coding transcripts (sub-section Creating non-redundant sets of coding and non-coding transcripts). All transcripts which had an orthology to more than one protein with the filters used above, were called chimeras. For every chimeric transcript, individual genes from gene pairs were extracted based on the orthologous coordinates obtained from BLASTx as described in Methods under the section prediction of coding and non-coding transcripts. To compensate for non-orthologous termini of the genes from BLAST in the first and last exons, the individual transcripts were extended by 75 bases on either side. These extended gene pairs were aligned against multiple plant genomes (in Table 2, downloaded from Phytozome [32]) at protein level (using > = 60% identity and > = 60% query coverage per subject as the filters) to (i) ignore chimeras arising out of mis-assembly and identify true chimeras, and (ii) identify Caryophyllales-specific and Ah-specific chimeras. In order to eliminate false positives stemming from transcriptome assembly tools, we have excluded transcripts comprising potential genes that had a significant hit in identical coordinates in the genome and those that were hitting to the genome in a region that was not annotated in the corresponding release of the GFF3 file. Thus, only those chimeras were selected where the gene pairs were in the same gene loci (i.e., either overlapping in coding sequences or closer than 50 kilobases as previously reported [33]), either in a *convergent*, *divergent or co-oriented* manner. A Venn diagram for the chimeric transcripts getting validated in six genomes was drawn using JVenn [34].

### Experimental validation of chimera

Primers were designed from a convergent gene pair, two forward and three reverse primers for each of the genes in chimeric transcripts, using Primer3plus [35] with some manual adjustments. Genomic DNA and total RNA were extracted from mature leaves using the conventional extraction methods (CTAB and trizol, respectively). Reverse transcription of the extracted RNA was done using the conventional protocol using M-MuLV reverse transcriptase. Amplification of the DNA fragments was done using DNAzyme (*Taq* DNA polymerase (in house preparation) and the gene specific primers. PCR was performed using 10 pmol of each oligonucleotide primer (S3 Fig) and 1 U of Taq polymerase (Thermoscientific) in a 10uL reaction volume. The reaction conditions for PCR included denaturation (94°C) for 1 min, primer annealing (52°C) for 1 min and extension (72°C) for 3 min for 35 cycles in 10 ml reaction volume. PCR Products was resolved on 1% agarose gel to check the DNA amplification.

## Supporting information

S1 Fig GO classification of caryophyllales-specific fusion genes in the functional categories of biological processes (a), cellular components (b) and molecular functions (c). (DOCX)

S2 Fig Configuration of SAUR genes on plant genomes that are clustered in a chimeric transcript. (DOCX)

S3 Fig Primers designed from DHDPS1-alpha glucosidase overlapping region for PCR validation from gDNA and cDNA. Two forward primers from 3’ terminus of DHDPS1 CDS, two forward primers (reverse complement of the sequence) from 3’ terminus of alpha glucosidase CDS and 6 primers (with 3 being reverse complement of the other 3) from the overlapping region were designed. The gFP primers also overlap.
(DOCX)

S1 Table List of 20 transcriptomes sequenced across different tissues and stages of growth from *A*. *hypochondriacus*. *Libraries sequenced previously [6].
(DOCX)

S2 Table List of 32 *de novo* assemblies done from the 20 transcriptomes of *A*. *hypochondriacus*. (DOCX)

S1 Dataset Annotation of the 1810 potential chimeric transcripts. (XLSX)

S2 Dataset Inter-genic distances of the genes within 581 validated chimeras. (XLSX)

S3 Dataset RPKM values of the 581 validated chimeric transcripts. (XLSX)

S4 Dataset Expression profiles of the 581 validated chimeric transcripts. (ZIP)

S5 Dataset Pearson’s correlation for the expression profiles of gene pairs within the 581 chimeric transcripts. (XLSX)

## Abbreviations

Ah: *Amaranthus hypochondriacus*
At: *Arabidopsis thaliana*
Bv: *Beta vulgaris*
Gm: *Glycine max*
Sl: *Solanum lycopersicum*
Zm: *Zea mays*
