# Supplementary figures and images for: Differential genomic arrangements in Caryophyllales through deep transcriptome sequencing of A. hypochondriacus (part 1 of 3)
Source: PLoS One. 2017 Aug 7;12(8):e0180528. doi: 10.1371/journal.pone.0180528 (PMC5546567; doi:10.1371/journal.pone.0180528)

| (a) 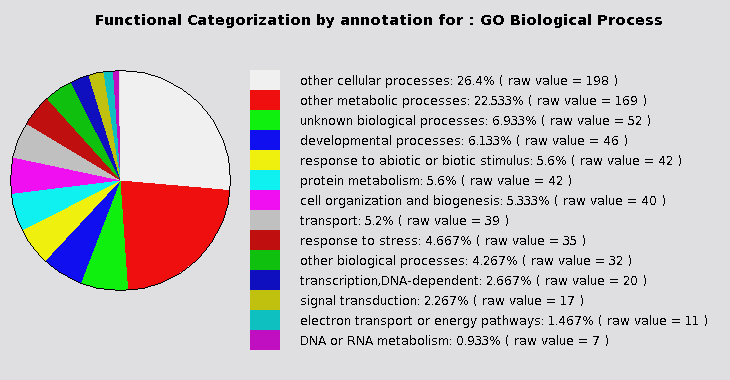 |
| --- |
| (b) 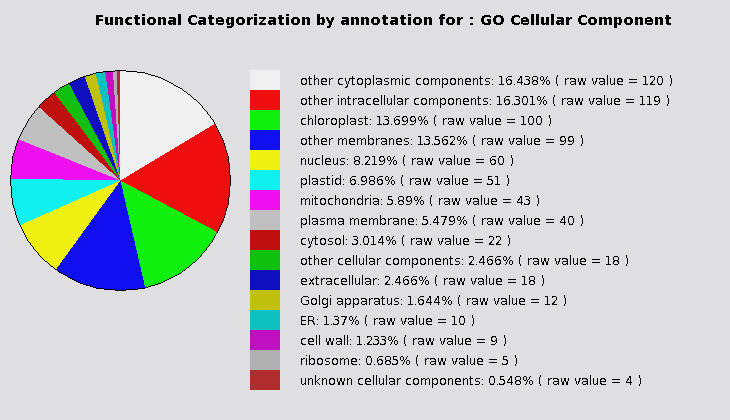 |
| (c) 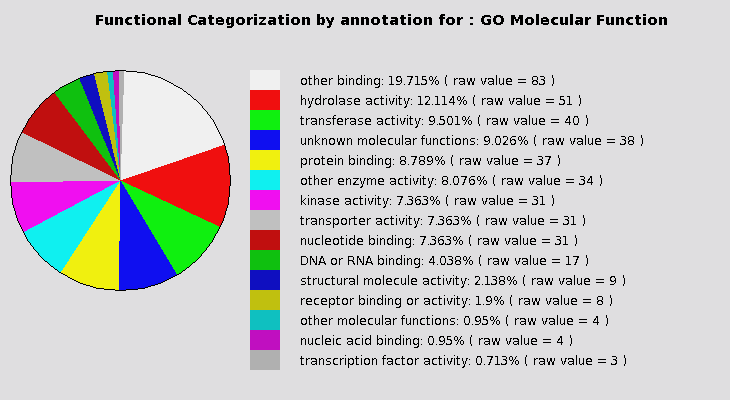 |

Supplement: S1 Fig — (DOCX) [file pone.0180528.s001.docx]

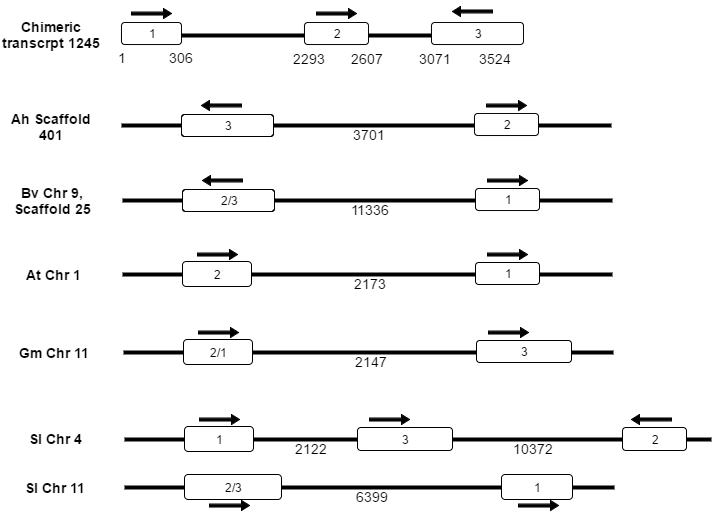

Supplement: S2 Fig — (DOCX) [file pone.0180528.s002.docx]

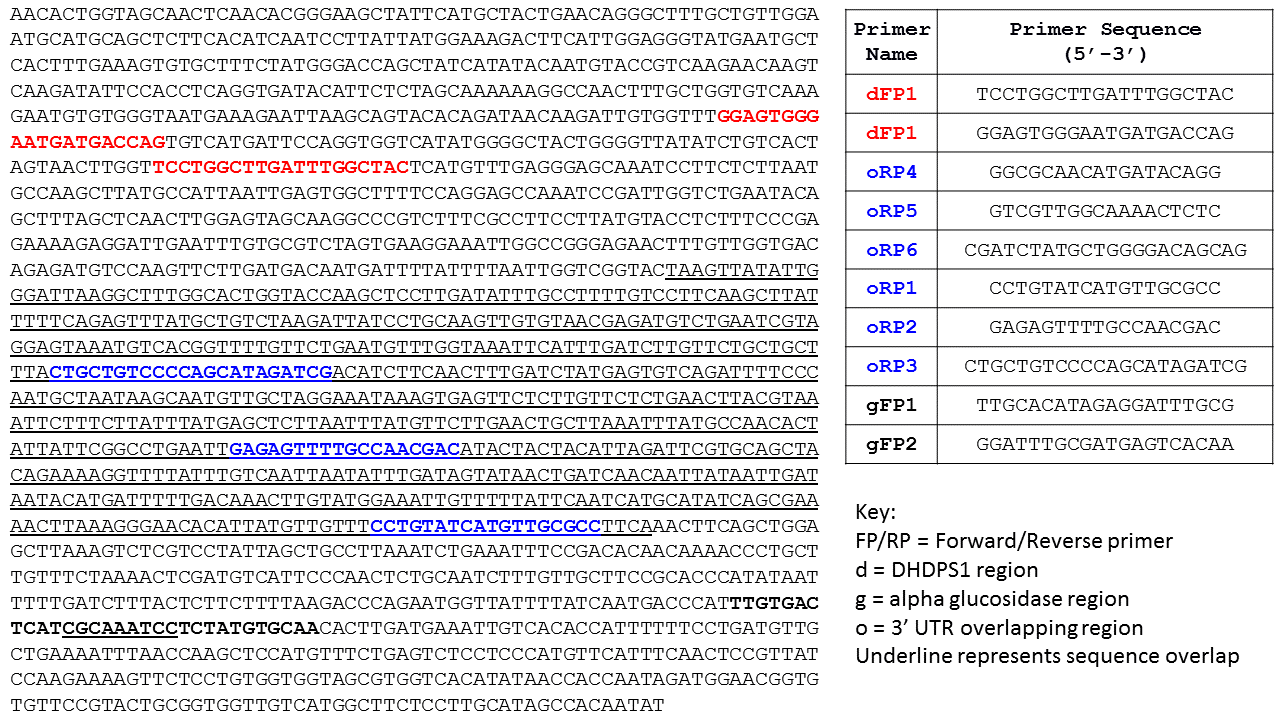

Supplement: S3 Fig — Two forward primers from 3’ terminus of DHDPS1 CDS, two forward primers (reverse complement of the sequence) from 3’ terminus of alpha glucosidase CDS and 6 primers (with 3 being reverse complement of the other 3) from the overlapping region were designed. The gFP primers also overlap. (DOCX) [file pone.0180528.s003.docx]

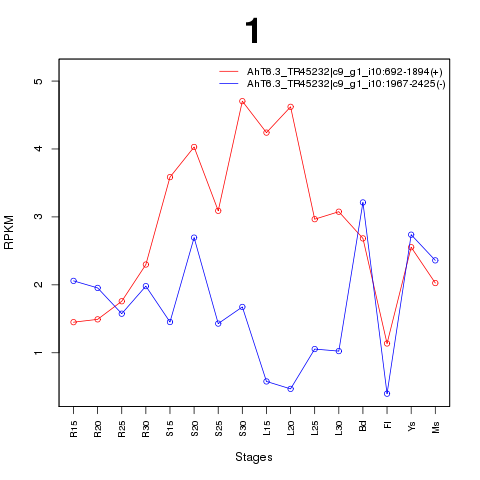

Supplement: S4 Dataset — (ZIP) [file pone.0180528.s009.zip › chimeras_581_PNGs/1.AhT6.3_TR45232_c9_g1_i10.rpkm.png]

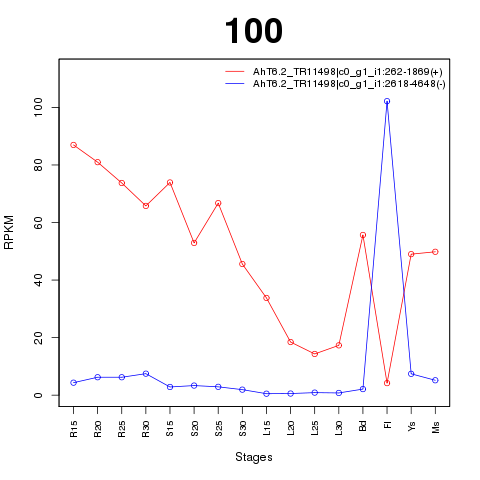

Supplement: S4 Dataset — (ZIP) [file pone.0180528.s009.zip › chimeras_581_PNGs/100.AhT6.2_TR11498_c0_g1_i1.rpkm.png]

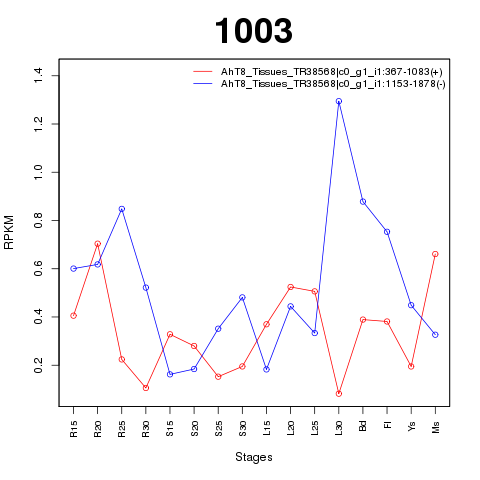

Supplement: S4 Dataset — (ZIP) [file pone.0180528.s009.zip › chimeras_581_PNGs/1003.AhT8_Tissues_TR38568_c0_g1_i1.rpkm.png]

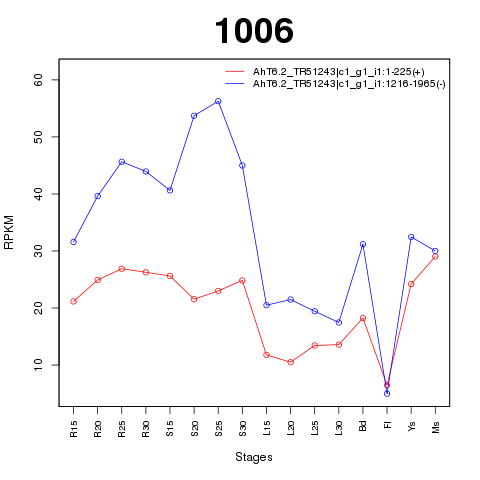

Supplement: S4 Dataset — (ZIP) [file pone.0180528.s009.zip › chimeras_581_PNGs/1006.AhT6.2_TR51243_c1_g1_i1.rpkm.png]

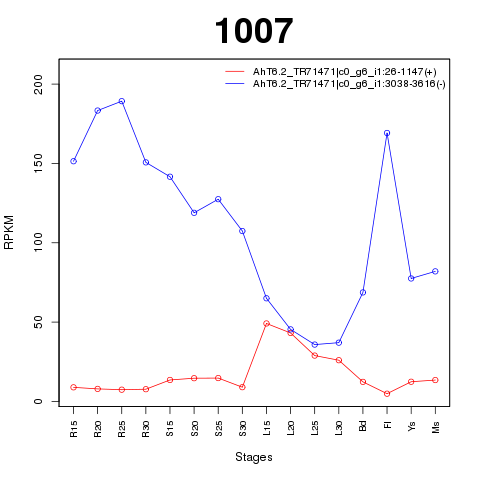

Supplement: S4 Dataset — (ZIP) [file pone.0180528.s009.zip › chimeras_581_PNGs/1007.AhT6.2_TR71471_c0_g6_i1.rpkm.png]

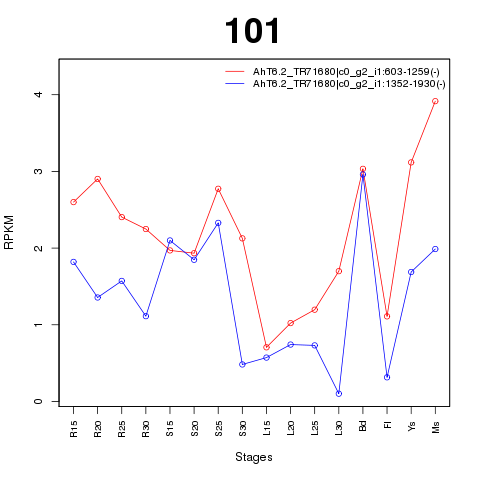

Supplement: S4 Dataset — (ZIP) [file pone.0180528.s009.zip › chimeras_581_PNGs/101.AhT6.2_TR71680_c0_g2_i1.rpkm.png]

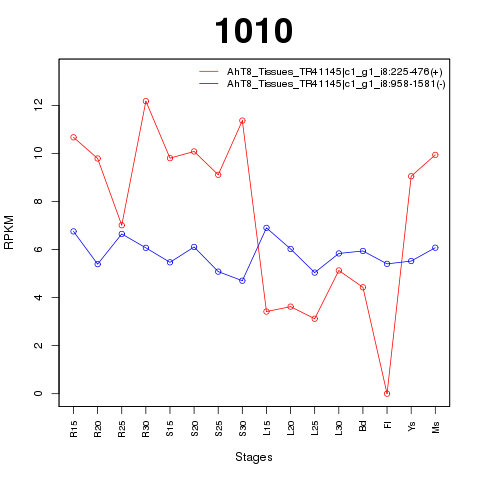

Supplement: S4 Dataset — (ZIP) [file pone.0180528.s009.zip › chimeras_581_PNGs/1010.AhT8_Tissues_TR41145_c1_g1_i8.rpkm.png]

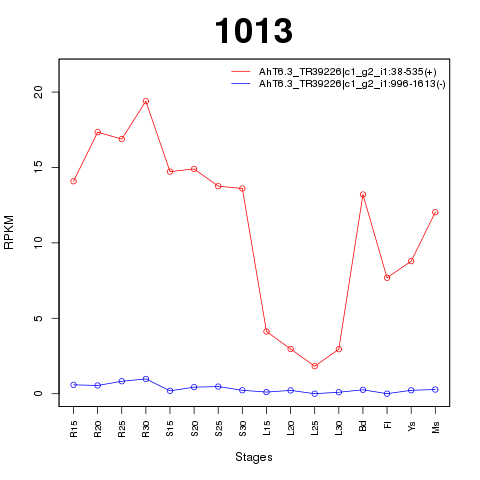

Supplement: S4 Dataset — (ZIP) [file pone.0180528.s009.zip › chimeras_581_PNGs/1013.AhT6.3_TR39226_c1_g2_i1.rpkm.png]

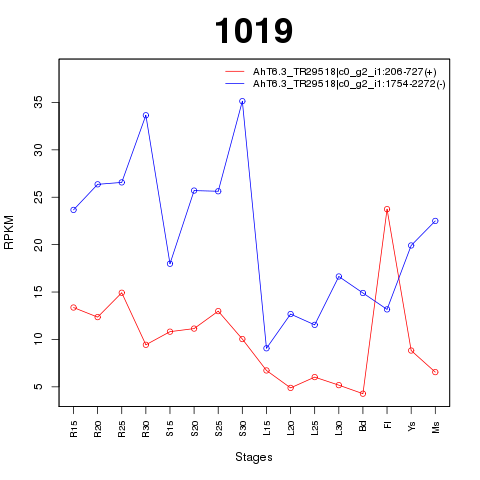

Supplement: S4 Dataset — (ZIP) [file pone.0180528.s009.zip › chimeras_581_PNGs/1019.AhT6.3_TR29518_c0_g2_i1.rpkm.png]

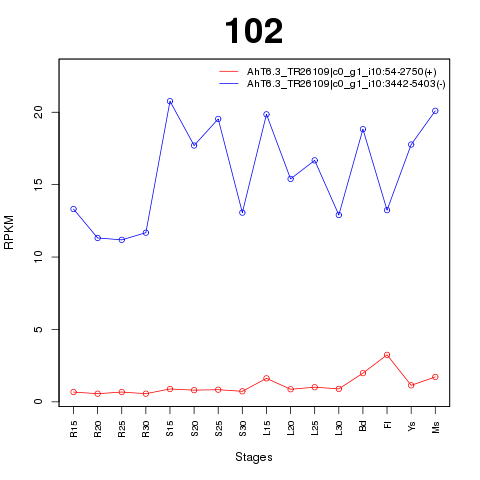

Supplement: S4 Dataset — (ZIP) [file pone.0180528.s009.zip › chimeras_581_PNGs/102.AhT6.3_TR26109_c0_g1_i10.rpkm.png]

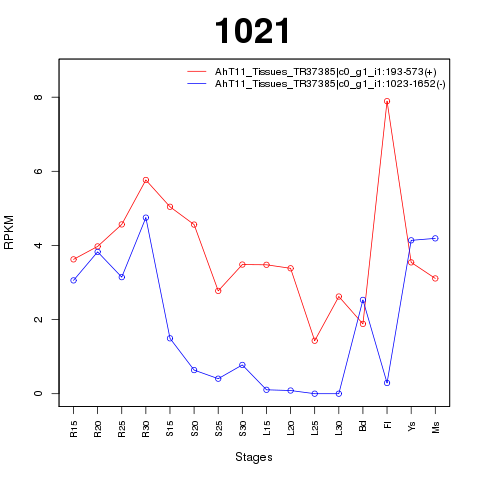

Supplement: S4 Dataset — (ZIP) [file pone.0180528.s009.zip › chimeras_581_PNGs/1021.AhT11_Tissues_TR37385_c0_g1_i1.rpkm.png]

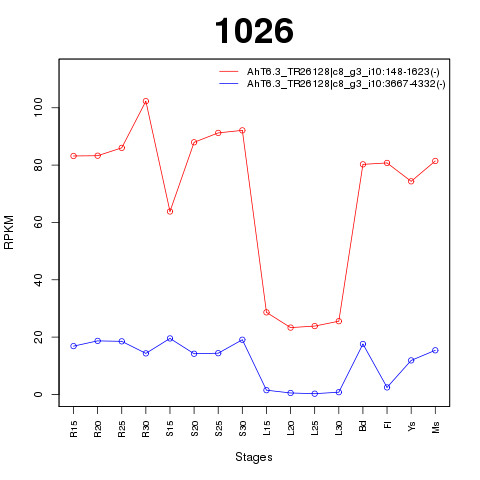

Supplement: S4 Dataset — (ZIP) [file pone.0180528.s009.zip › chimeras_581_PNGs/1026.AhT6.3_TR26128_c8_g3_i10.rpkm.png]

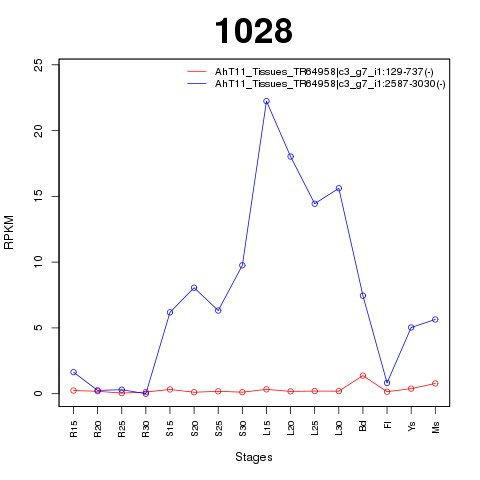

Supplement: S4 Dataset — (ZIP) [file pone.0180528.s009.zip › chimeras_581_PNGs/1028.AhT11_Tissues_TR64958_c3_g7_i1.rpkm.png]

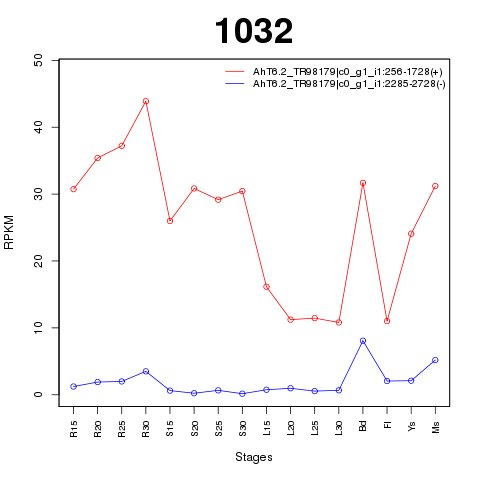

Supplement: S4 Dataset — (ZIP) [file pone.0180528.s009.zip › chimeras_581_PNGs/1032.AhT6.2_TR98179_c0_g1_i1.rpkm.png]

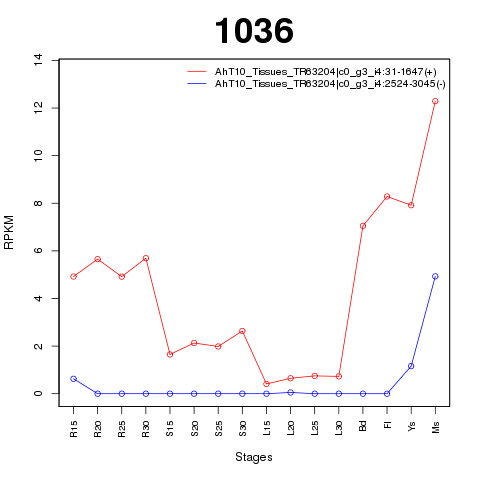

Supplement: S4 Dataset — (ZIP) [file pone.0180528.s009.zip › chimeras_581_PNGs/1036.AhT10_Tissues_TR63204_c0_g3_i4.rpkm.png]

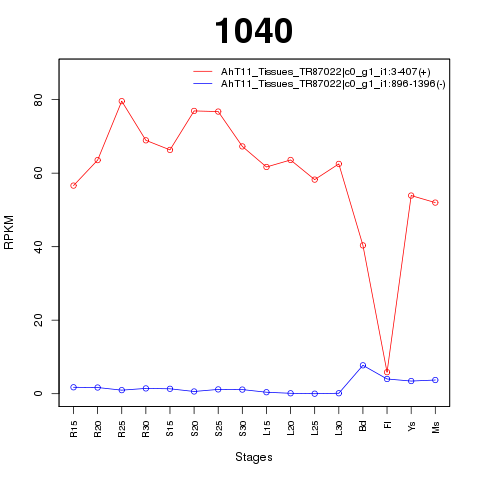

Supplement: S4 Dataset — (ZIP) [file pone.0180528.s009.zip › chimeras_581_PNGs/1040.AhT11_Tissues_TR87022_c0_g1_i1.rpkm.png]

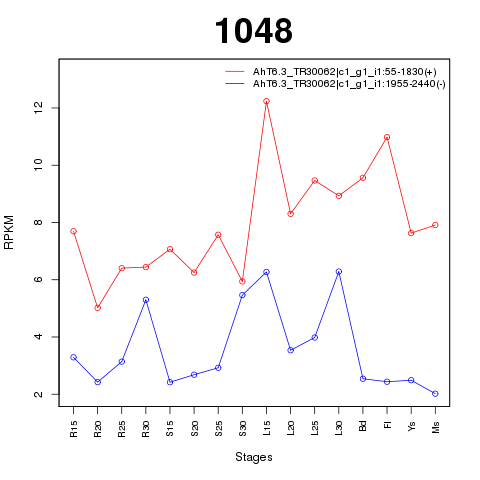

Supplement: S4 Dataset — (ZIP) [file pone.0180528.s009.zip › chimeras_581_PNGs/1048.AhT6.3_TR30062_c1_g1_i1.rpkm.png]

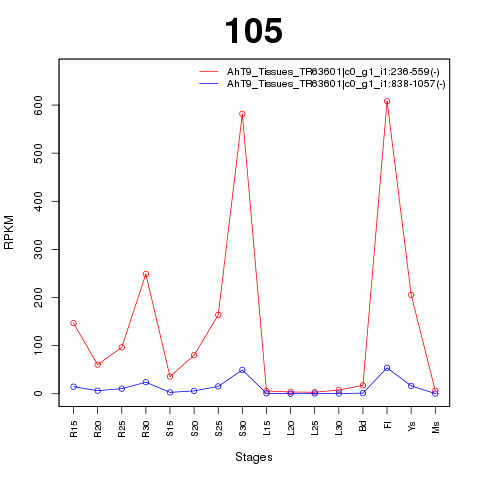

Supplement: S4 Dataset — (ZIP) [file pone.0180528.s009.zip › chimeras_581_PNGs/105.AhT9_Tissues_TR63601_c0_g1_i1.rpkm.png]

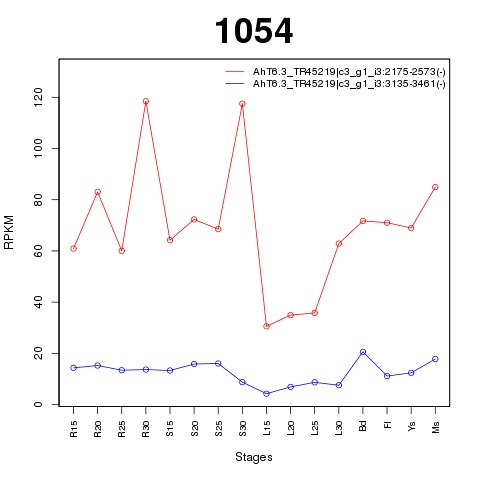

Supplement: S4 Dataset — (ZIP) [file pone.0180528.s009.zip › chimeras_581_PNGs/1054.AhT6.3_TR45219_c3_g1_i3.rpkm.png]

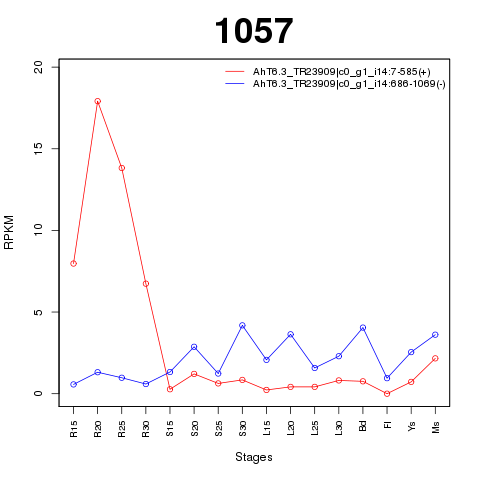

Supplement: S4 Dataset — (ZIP) [file pone.0180528.s009.zip › chimeras_581_PNGs/1057.AhT6.3_TR23909_c0_g1_i14.rpkm.png]

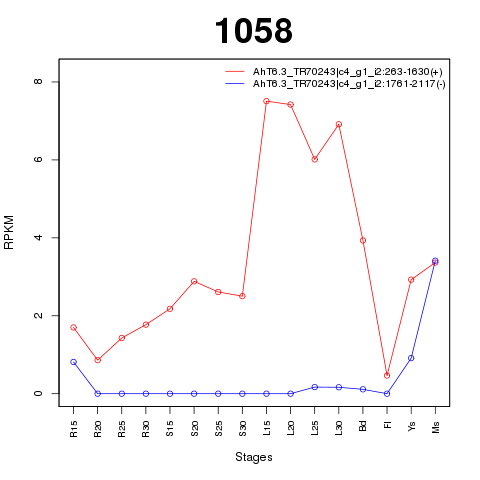

Supplement: S4 Dataset — (ZIP) [file pone.0180528.s009.zip › chimeras_581_PNGs/1058.AhT6.3_TR70243_c4_g1_i2.rpkm.png]

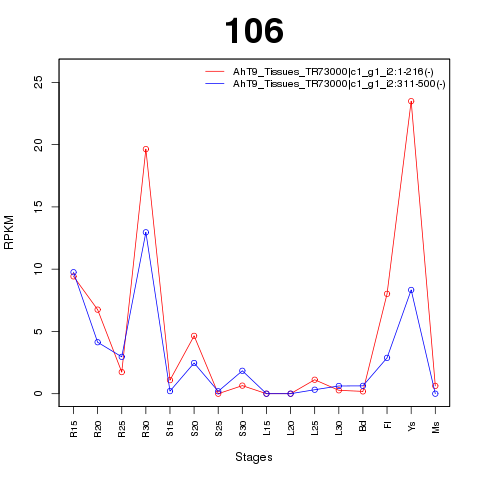

Supplement: S4 Dataset — (ZIP) [file pone.0180528.s009.zip › chimeras_581_PNGs/106.AhT9_Tissues_TR73000_c1_g1_i2.rpkm.png]

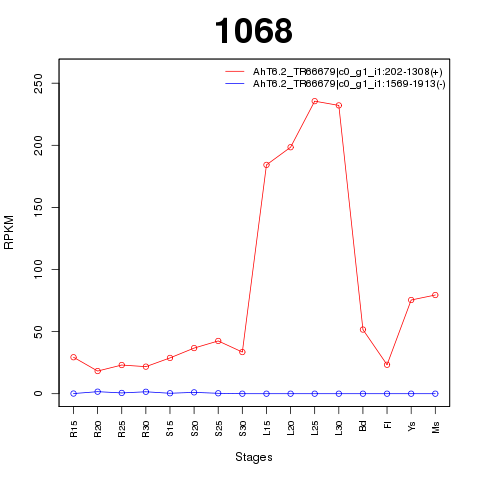

Supplement: S4 Dataset — (ZIP) [file pone.0180528.s009.zip › chimeras_581_PNGs/1068.AhT6.2_TR66679_c0_g1_i1.rpkm.png]

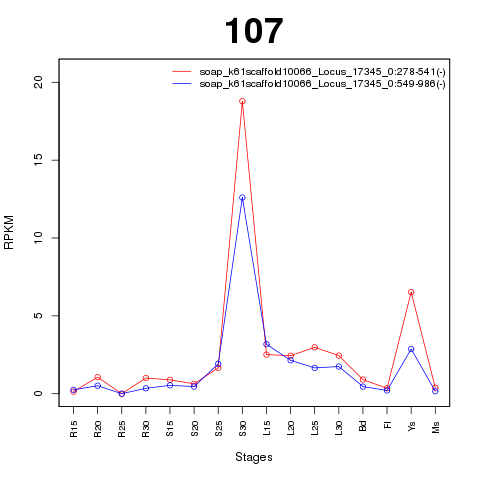

Supplement: S4 Dataset — (ZIP) [file pone.0180528.s009.zip › chimeras_581_PNGs/107.soap_k61scaffold10066_Locus_17345_0.rpkm.png]

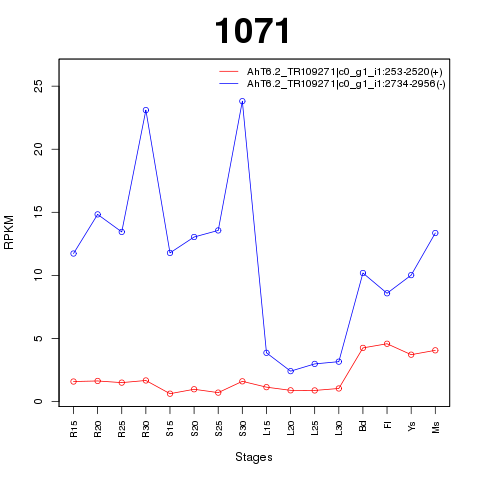

Supplement: S4 Dataset — (ZIP) [file pone.0180528.s009.zip › chimeras_581_PNGs/1071.AhT6.2_TR109271_c0_g1_i1.rpkm.png]

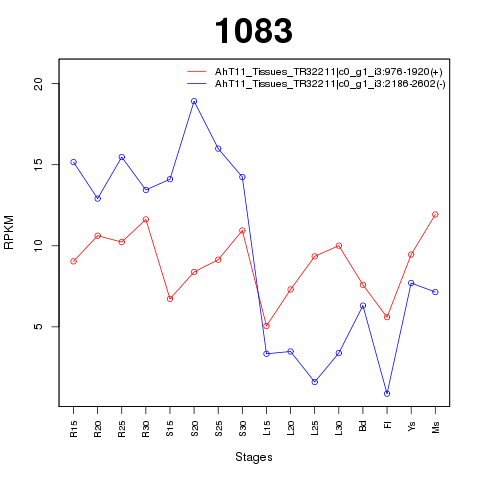

Supplement: S4 Dataset — (ZIP) [file pone.0180528.s009.zip › chimeras_581_PNGs/1083.AhT11_Tissues_TR32211_c0_g1_i3.rpkm.png]

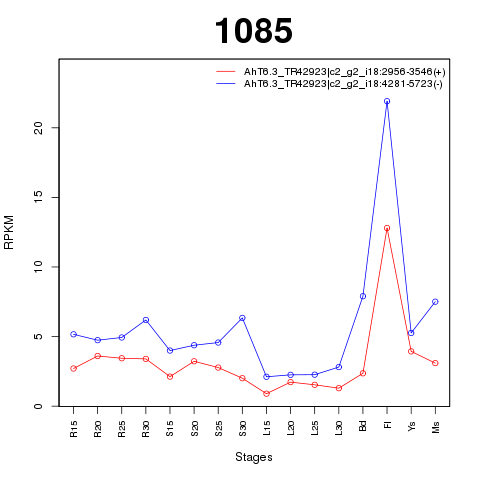

Supplement: S4 Dataset — (ZIP) [file pone.0180528.s009.zip › chimeras_581_PNGs/1085.AhT6.3_TR42923_c2_g2_i18.rpkm.png]

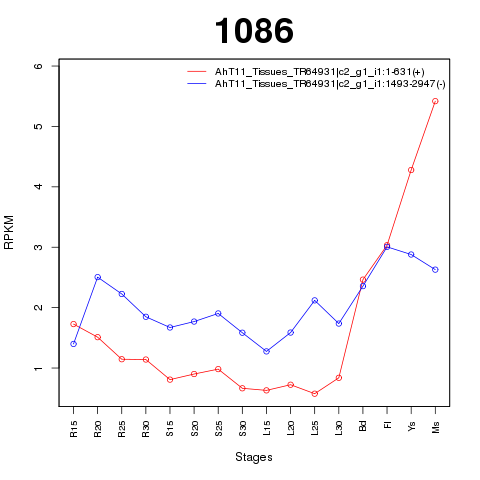

Supplement: S4 Dataset — (ZIP) [file pone.0180528.s009.zip › chimeras_581_PNGs/1086.AhT11_Tissues_TR64931_c2_g1_i1.rpkm.png]

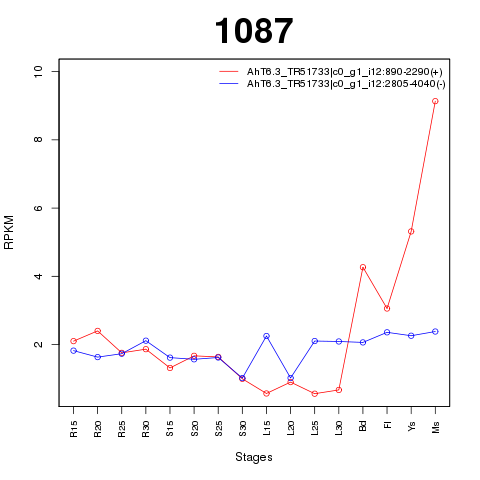

Supplement: S4 Dataset — (ZIP) [file pone.0180528.s009.zip › chimeras_581_PNGs/1087.AhT6.3_TR51733_c0_g1_i12.rpkm.png]

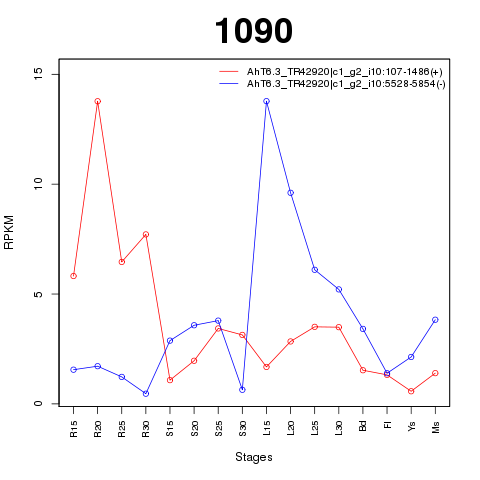

Supplement: S4 Dataset — (ZIP) [file pone.0180528.s009.zip › chimeras_581_PNGs/1090.AhT6.3_TR42920_c1_g2_i10.rpkm.png]

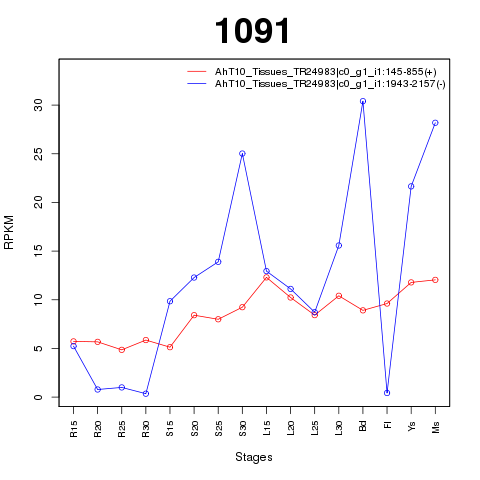

Supplement: S4 Dataset — (ZIP) [file pone.0180528.s009.zip › chimeras_581_PNGs/1091.AhT10_Tissues_TR24983_c0_g1_i1.rpkm.png]

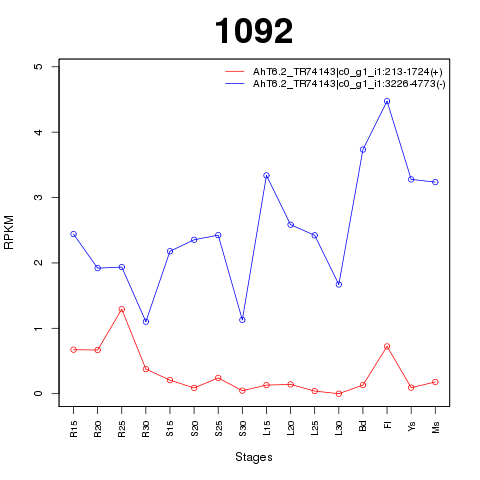

Supplement: S4 Dataset — (ZIP) [file pone.0180528.s009.zip › chimeras_581_PNGs/1092.AhT6.2_TR74143_c0_g1_i1.rpkm.png]

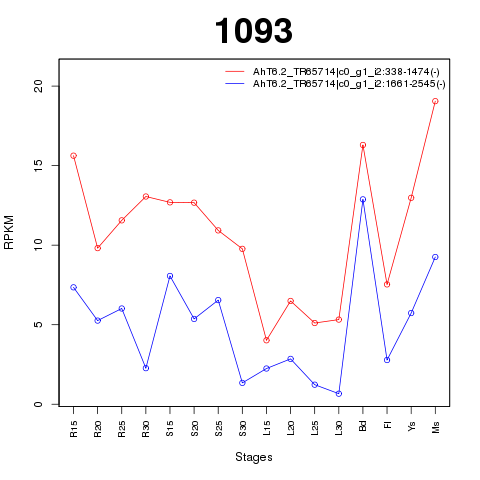

Supplement: S4 Dataset — (ZIP) [file pone.0180528.s009.zip › chimeras_581_PNGs/1093.AhT6.2_TR65714_c0_g1_i2.rpkm.png]

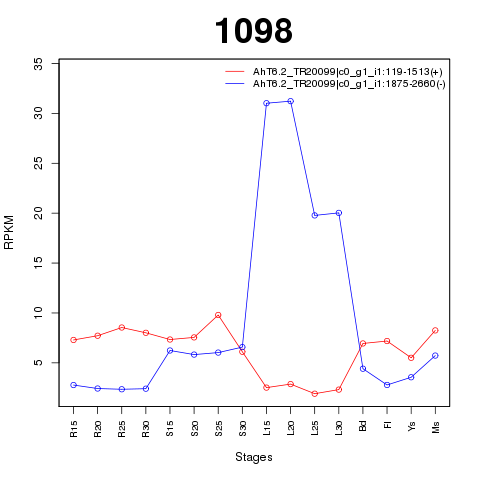

Supplement: S4 Dataset — (ZIP) [file pone.0180528.s009.zip › chimeras_581_PNGs/1098.AhT6.2_TR20099_c0_g1_i1.rpkm.png]

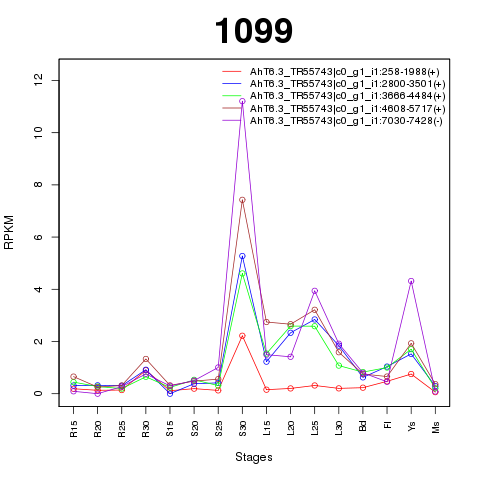

Supplement: S4 Dataset — (ZIP) [file pone.0180528.s009.zip › chimeras_581_PNGs/1099.AhT6.3_TR55743_c0_g1_i1.rpkm.png]

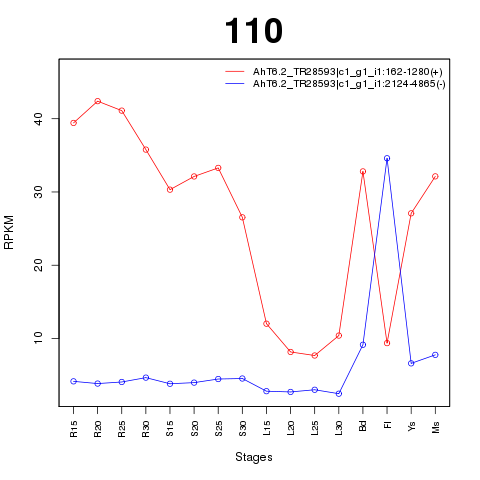

Supplement: S4 Dataset — (ZIP) [file pone.0180528.s009.zip › chimeras_581_PNGs/110.AhT6.2_TR28593_c1_g1_i1.rpkm.png]

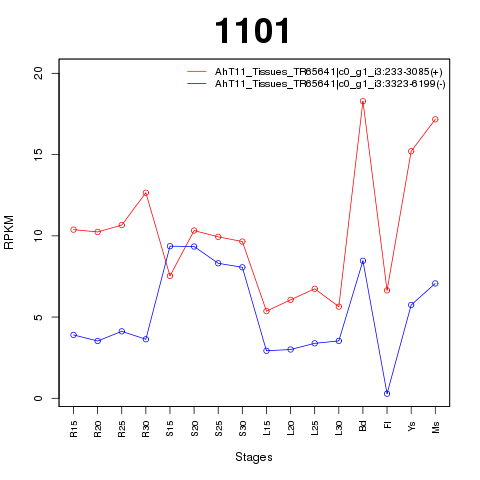

Supplement: S4 Dataset — (ZIP) [file pone.0180528.s009.zip › chimeras_581_PNGs/1101.AhT11_Tissues_TR65641_c0_g1_i3.rpkm.png]

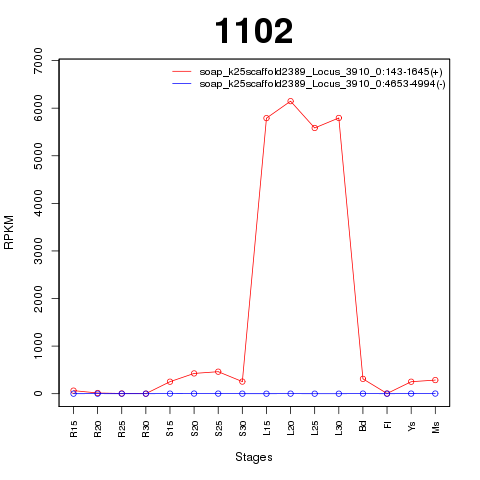

Supplement: S4 Dataset — (ZIP) [file pone.0180528.s009.zip › chimeras_581_PNGs/1102.soap_k25scaffold2389_Locus_3910_0.rpkm.png]

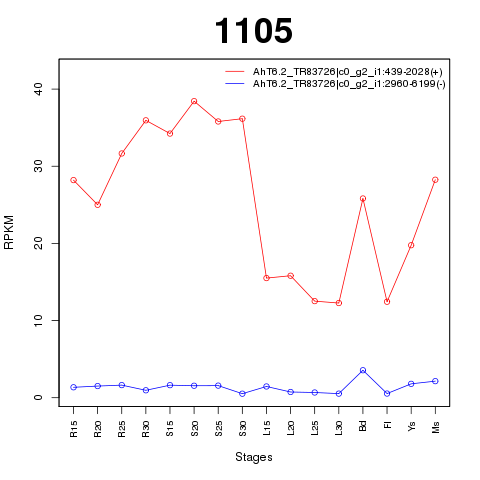

Supplement: S4 Dataset — (ZIP) [file pone.0180528.s009.zip › chimeras_581_PNGs/1105.AhT6.2_TR83726_c0_g2_i1.rpkm.png]

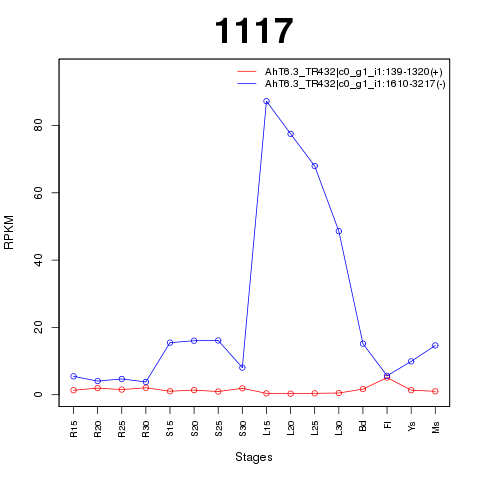

Supplement: S4 Dataset — (ZIP) [file pone.0180528.s009.zip › chimeras_581_PNGs/1117.AhT6.3_TR432_c0_g1_i1.rpkm.png]

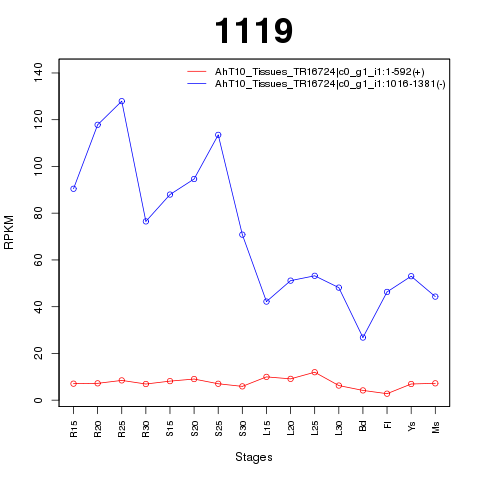

Supplement: S4 Dataset — (ZIP) [file pone.0180528.s009.zip › chimeras_581_PNGs/1119.AhT10_Tissues_TR16724_c0_g1_i1.rpkm.png]

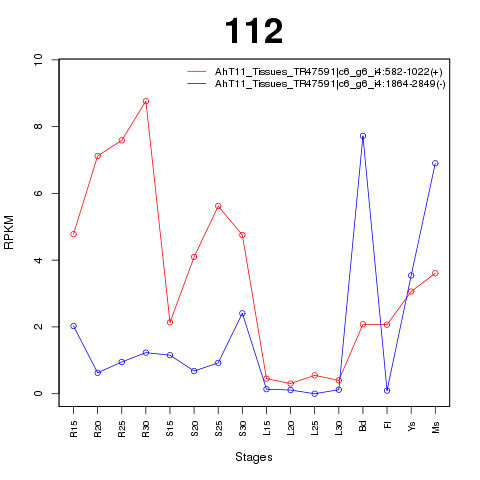

Supplement: S4 Dataset — (ZIP) [file pone.0180528.s009.zip › chimeras_581_PNGs/112.AhT11_Tissues_TR47591_c6_g6_i4.rpkm.png]

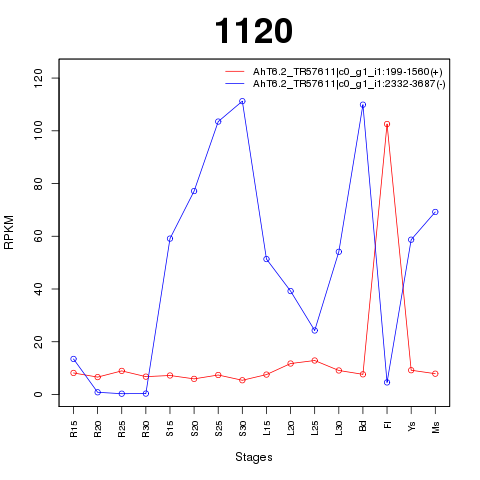

Supplement: S4 Dataset — (ZIP) [file pone.0180528.s009.zip › chimeras_581_PNGs/1120.AhT6.2_TR57611_c0_g1_i1.rpkm.png]

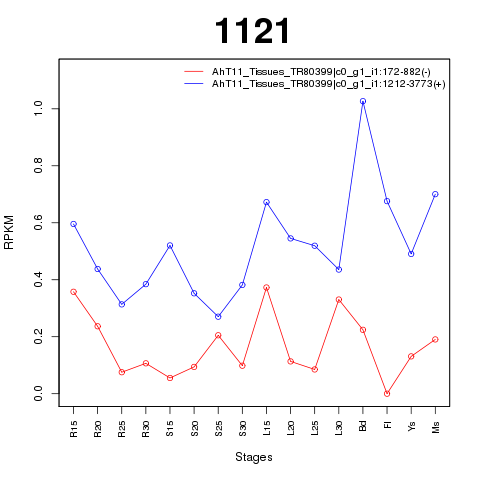

Supplement: S4 Dataset — (ZIP) [file pone.0180528.s009.zip › chimeras_581_PNGs/1121.AhT11_Tissues_TR80399_c0_g1_i1.rpkm.png]

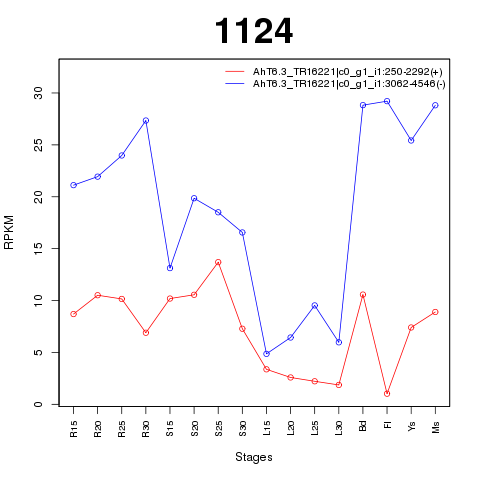

Supplement: S4 Dataset — (ZIP) [file pone.0180528.s009.zip › chimeras_581_PNGs/1124.AhT6.3_TR16221_c0_g1_i1.rpkm.png]

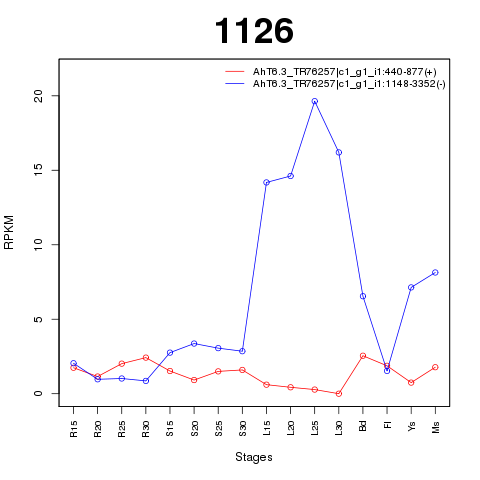

Supplement: S4 Dataset — (ZIP) [file pone.0180528.s009.zip › chimeras_581_PNGs/1126.AhT6.3_TR76257_c1_g1_i1.rpkm.png]

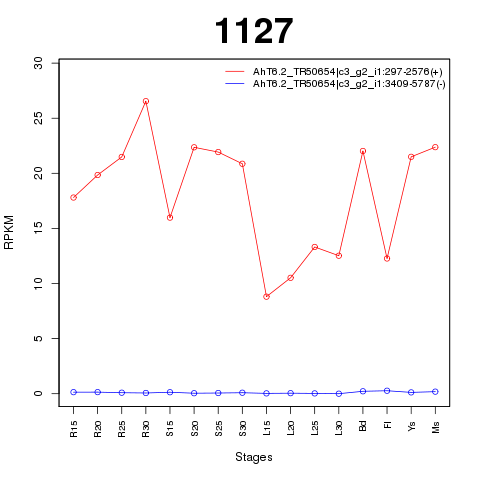

Supplement: S4 Dataset — (ZIP) [file pone.0180528.s009.zip › chimeras_581_PNGs/1127.AhT6.2_TR50654_c3_g2_i1.rpkm.png]

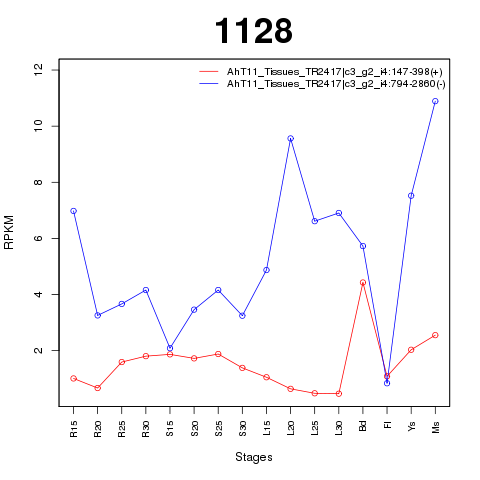

Supplement: S4 Dataset — (ZIP) [file pone.0180528.s009.zip › chimeras_581_PNGs/1128.AhT11_Tissues_TR2417_c3_g2_i4.rpkm.png]

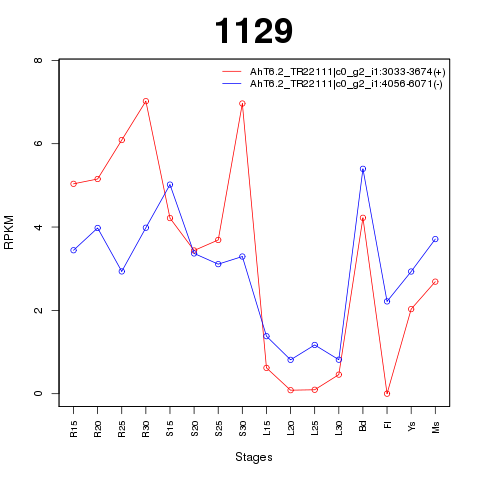

Supplement: S4 Dataset — (ZIP) [file pone.0180528.s009.zip › chimeras_581_PNGs/1129.AhT6.2_TR22111_c0_g2_i1.rpkm.png]

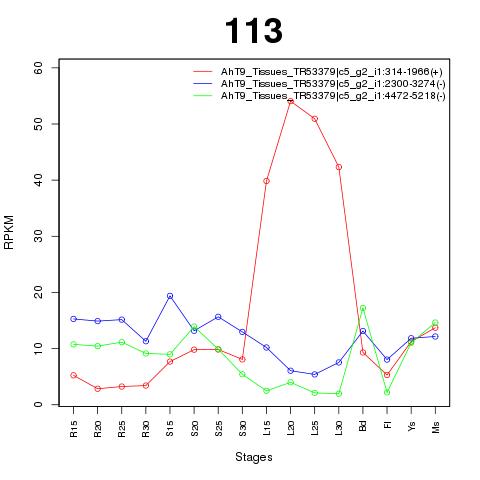

Supplement: S4 Dataset — (ZIP) [file pone.0180528.s009.zip › chimeras_581_PNGs/113.AhT9_Tissues_TR53379_c5_g2_i1.rpkm.png]

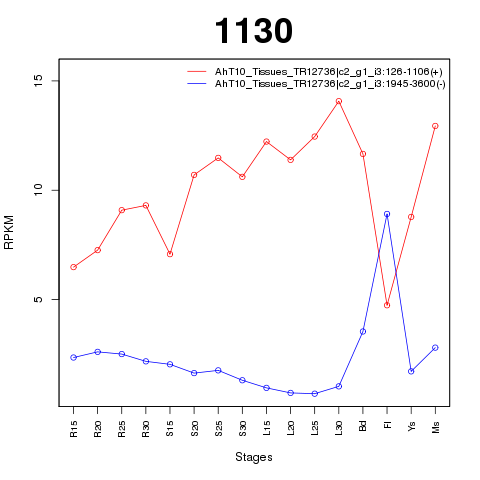

Supplement: S4 Dataset — (ZIP) [file pone.0180528.s009.zip › chimeras_581_PNGs/1130.AhT10_Tissues_TR12736_c2_g1_i3.rpkm.png]

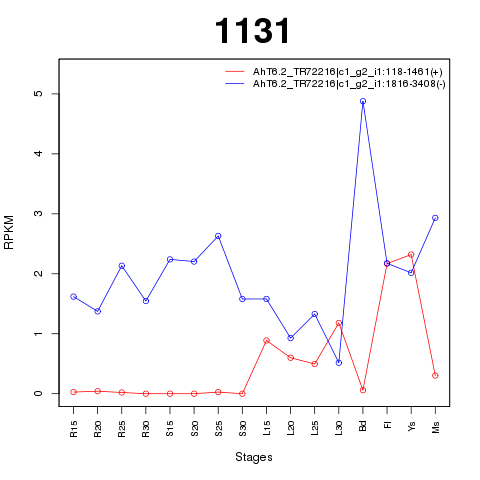

Supplement: S4 Dataset — (ZIP) [file pone.0180528.s009.zip › chimeras_581_PNGs/1131.AhT6.2_TR72216_c1_g2_i1.rpkm.png]

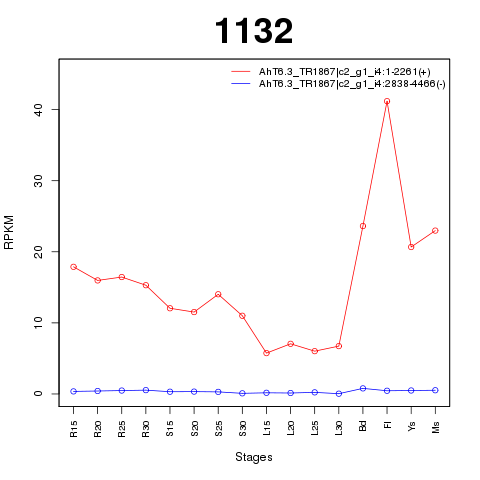

Supplement: S4 Dataset — (ZIP) [file pone.0180528.s009.zip › chimeras_581_PNGs/1132.AhT6.3_TR1867_c2_g1_i4.rpkm.png]

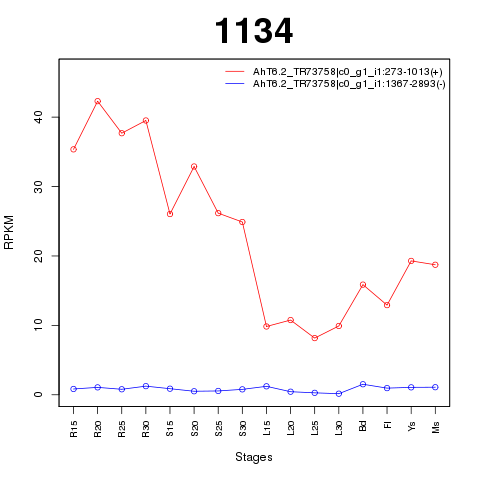

Supplement: S4 Dataset — (ZIP) [file pone.0180528.s009.zip › chimeras_581_PNGs/1134.AhT6.2_TR73758_c0_g1_i1.rpkm.png]

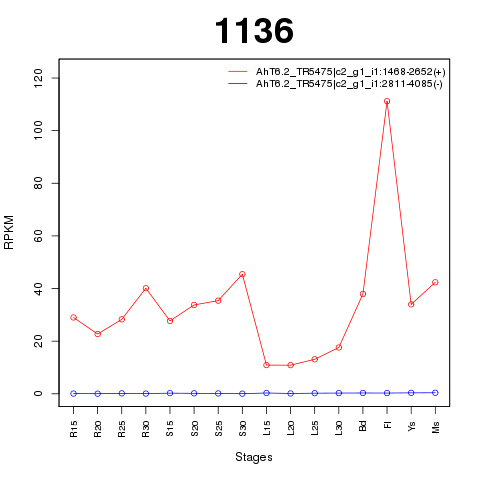

Supplement: S4 Dataset — (ZIP) [file pone.0180528.s009.zip › chimeras_581_PNGs/1136.AhT6.2_TR5475_c2_g1_i1.rpkm.png]

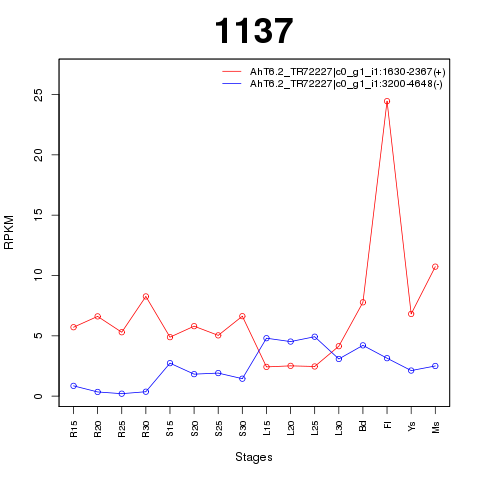

Supplement: S4 Dataset — (ZIP) [file pone.0180528.s009.zip › chimeras_581_PNGs/1137.AhT6.2_TR72227_c0_g1_i1.rpkm.png]

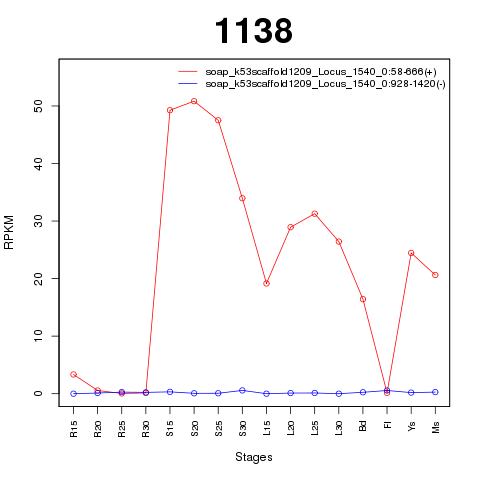

Supplement: S4 Dataset — (ZIP) [file pone.0180528.s009.zip › chimeras_581_PNGs/1138.soap_k53scaffold1209_Locus_1540_0.rpkm.png]

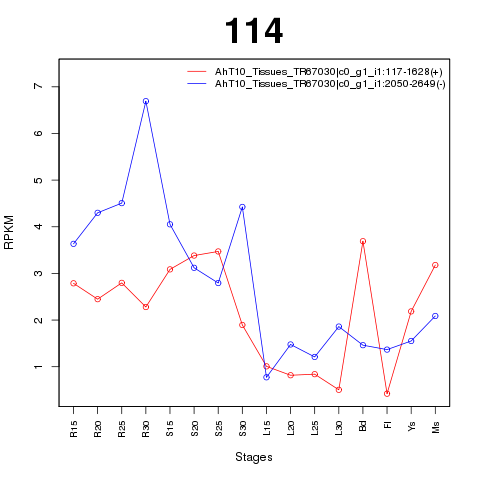

Supplement: S4 Dataset — (ZIP) [file pone.0180528.s009.zip › chimeras_581_PNGs/114.AhT10_Tissues_TR67030_c0_g1_i1.rpkm.png]

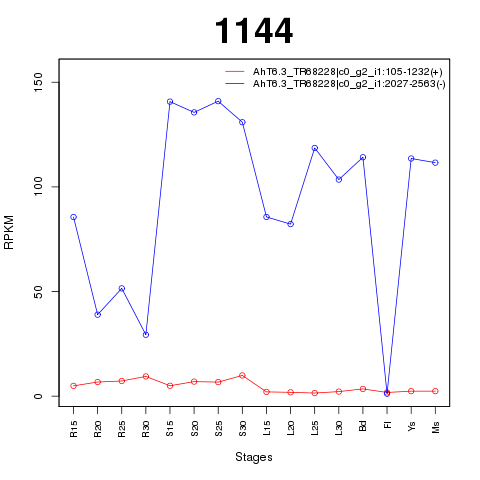

Supplement: S4 Dataset — (ZIP) [file pone.0180528.s009.zip › chimeras_581_PNGs/1144.AhT6.3_TR68228_c0_g2_i1.rpkm.png]

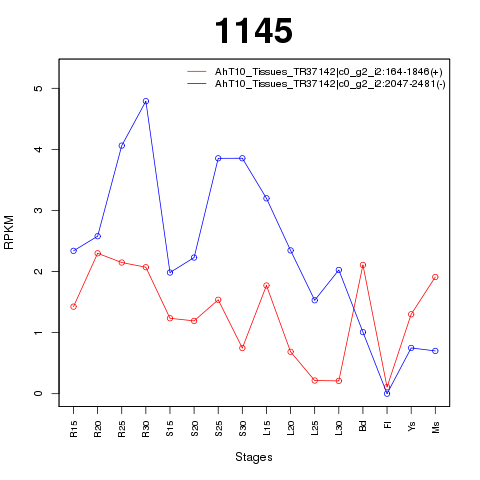

Supplement: S4 Dataset — (ZIP) [file pone.0180528.s009.zip › chimeras_581_PNGs/1145.AhT10_Tissues_TR37142_c0_g2_i2.rpkm.png]

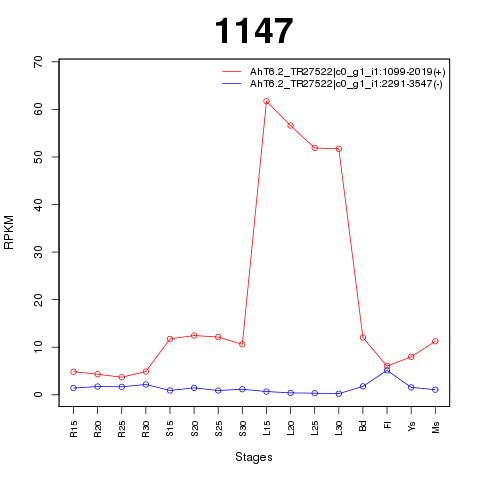

Supplement: S4 Dataset — (ZIP) [file pone.0180528.s009.zip › chimeras_581_PNGs/1147.AhT6.2_TR27522_c0_g1_i1.rpkm.png]

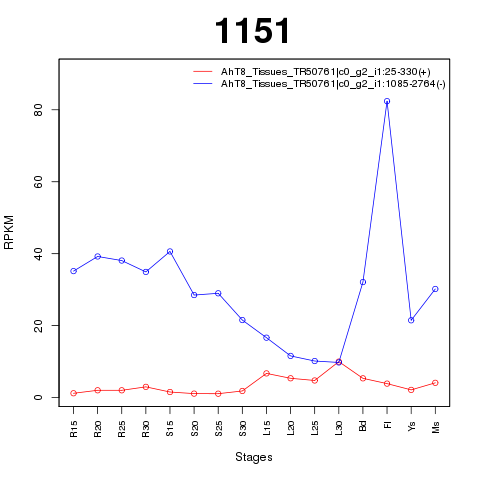

Supplement: S4 Dataset — (ZIP) [file pone.0180528.s009.zip › chimeras_581_PNGs/1151.AhT8_Tissues_TR50761_c0_g2_i1.rpkm.png]

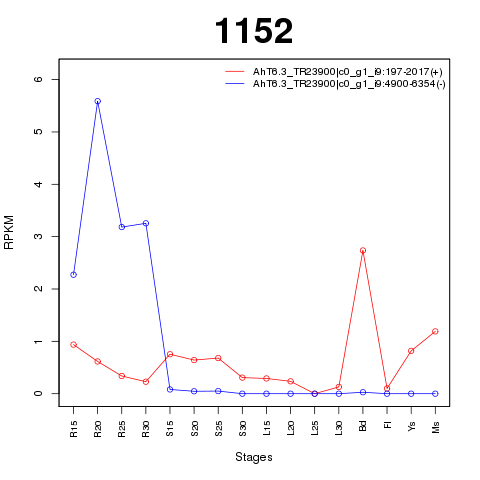

Supplement: S4 Dataset — (ZIP) [file pone.0180528.s009.zip › chimeras_581_PNGs/1152.AhT6.3_TR23900_c0_g1_i9.rpkm.png]

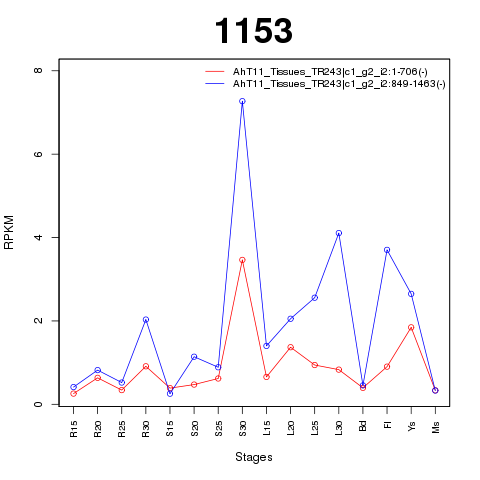

Supplement: S4 Dataset — (ZIP) [file pone.0180528.s009.zip › chimeras_581_PNGs/1153.AhT11_Tissues_TR243_c1_g2_i2.rpkm.png]

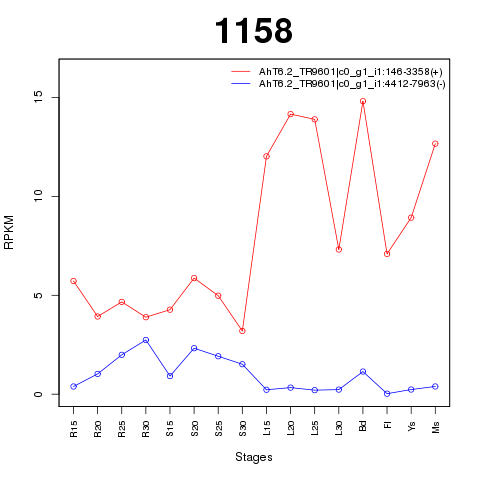

Supplement: S4 Dataset — (ZIP) [file pone.0180528.s009.zip › chimeras_581_PNGs/1158.AhT6.2_TR9601_c0_g1_i1.rpkm.png]

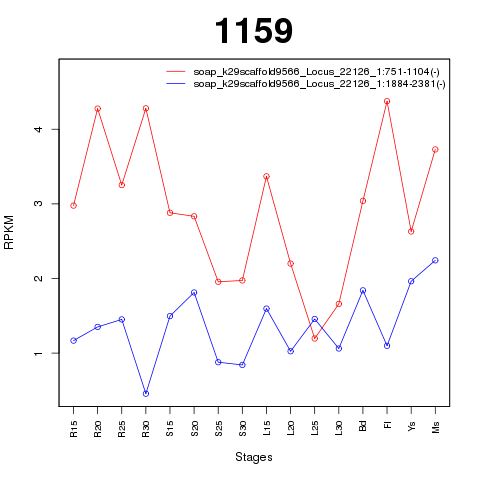

Supplement: S4 Dataset — (ZIP) [file pone.0180528.s009.zip › chimeras_581_PNGs/1159.soap_k29scaffold9566_Locus_22126_1.rpkm.png]

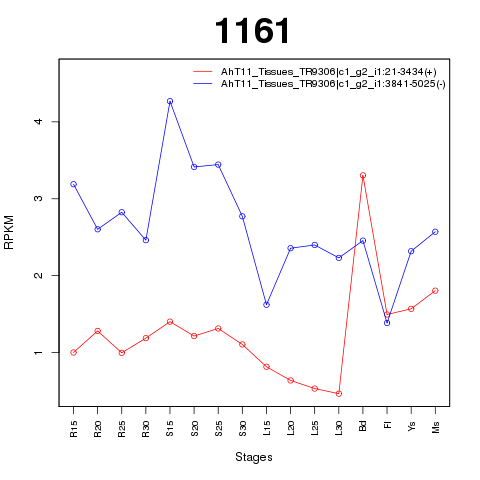

Supplement: S4 Dataset — (ZIP) [file pone.0180528.s009.zip › chimeras_581_PNGs/1161.AhT11_Tissues_TR9306_c1_g2_i1.rpkm.png]

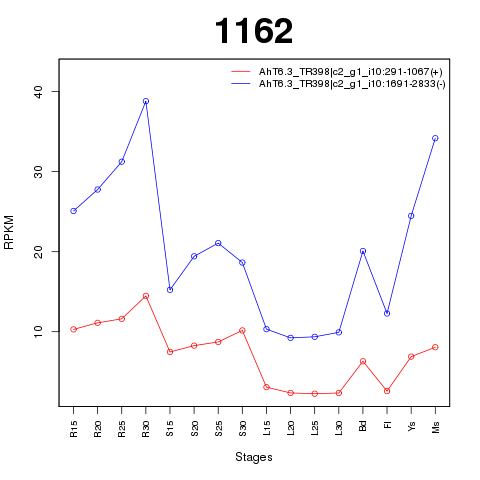

Supplement: S4 Dataset — (ZIP) [file pone.0180528.s009.zip › chimeras_581_PNGs/1162.AhT6.3_TR398_c2_g1_i10.rpkm.png]

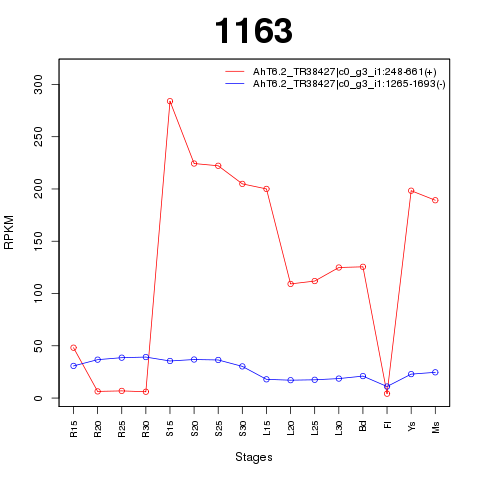

Supplement: S4 Dataset — (ZIP) [file pone.0180528.s009.zip › chimeras_581_PNGs/1163.AhT6.2_TR38427_c0_g3_i1.rpkm.png]

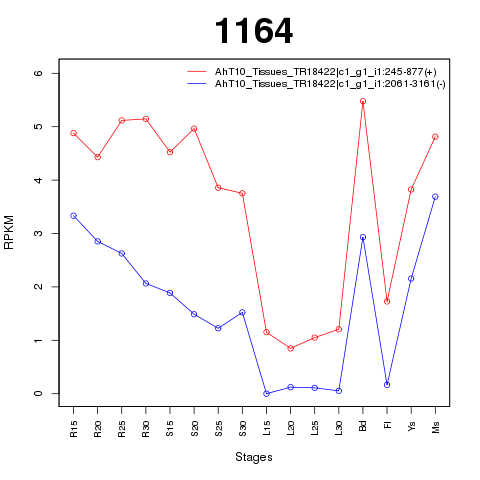

Supplement: S4 Dataset — (ZIP) [file pone.0180528.s009.zip › chimeras_581_PNGs/1164.AhT10_Tissues_TR18422_c1_g1_i1.rpkm.png]

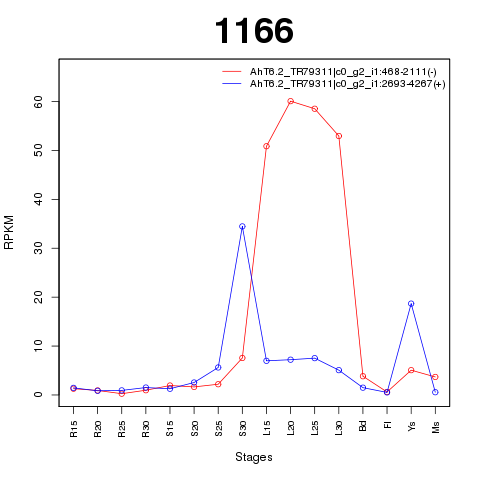

Supplement: S4 Dataset — (ZIP) [file pone.0180528.s009.zip › chimeras_581_PNGs/1166.AhT6.2_TR79311_c0_g2_i1.rpkm.png]

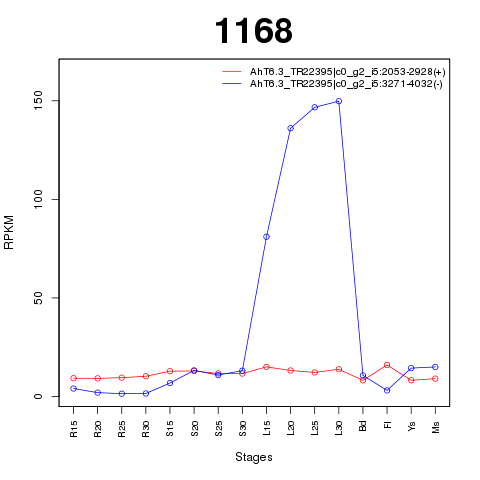

Supplement: S4 Dataset — (ZIP) [file pone.0180528.s009.zip › chimeras_581_PNGs/1168.AhT6.3_TR22395_c0_g2_i5.rpkm.png]

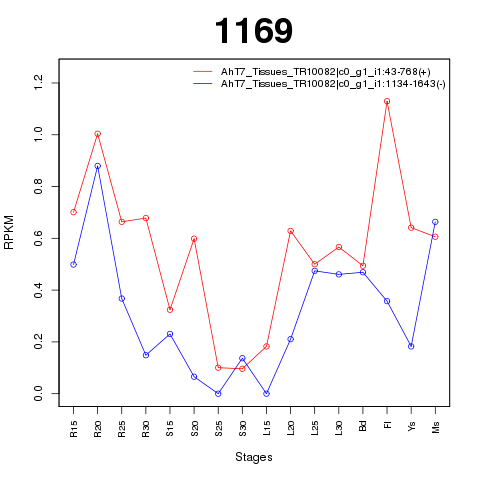

Supplement: S4 Dataset — (ZIP) [file pone.0180528.s009.zip › chimeras_581_PNGs/1169.AhT7_Tissues_TR10082_c0_g1_i1.rpkm.png]

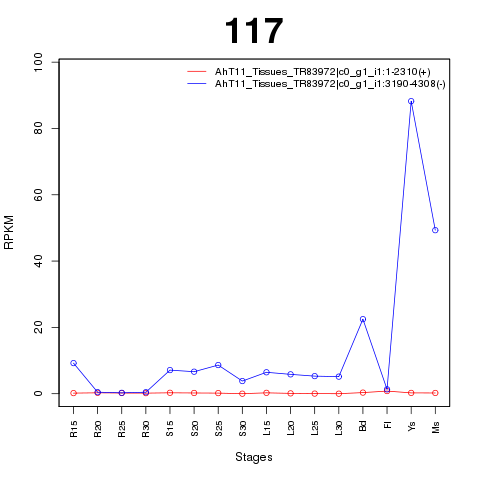

Supplement: S4 Dataset — (ZIP) [file pone.0180528.s009.zip › chimeras_581_PNGs/117.AhT11_Tissues_TR83972_c0_g1_i1.rpkm.png]

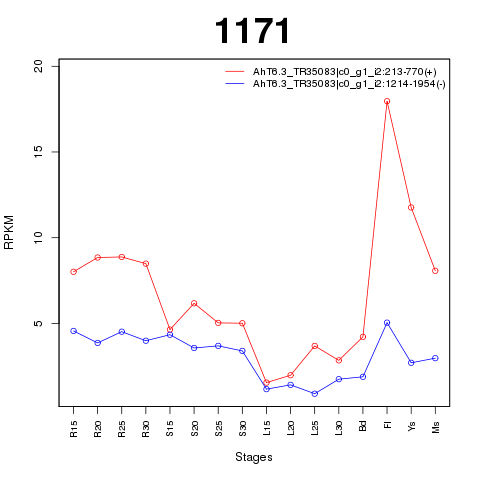

Supplement: S4 Dataset — (ZIP) [file pone.0180528.s009.zip › chimeras_581_PNGs/1171.AhT6.3_TR35083_c0_g1_i2.rpkm.png]

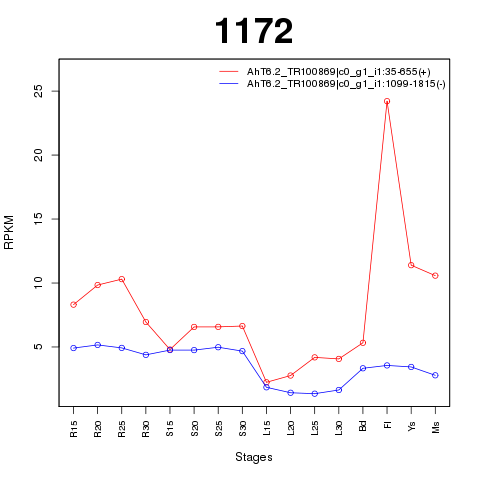

Supplement: S4 Dataset — (ZIP) [file pone.0180528.s009.zip › chimeras_581_PNGs/1172.AhT6.2_TR100869_c0_g1_i1.rpkm.png]

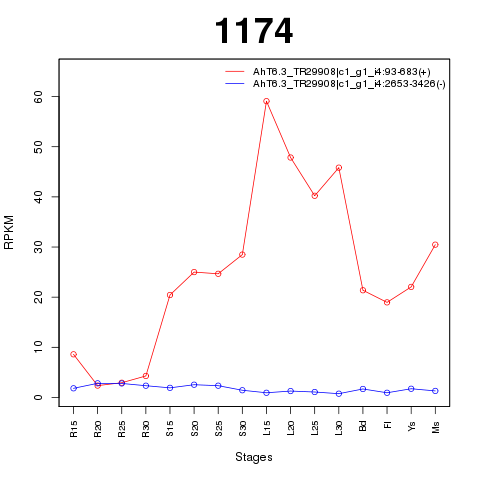

Supplement: S4 Dataset — (ZIP) [file pone.0180528.s009.zip › chimeras_581_PNGs/1174.AhT6.3_TR29908_c1_g1_i4.rpkm.png]

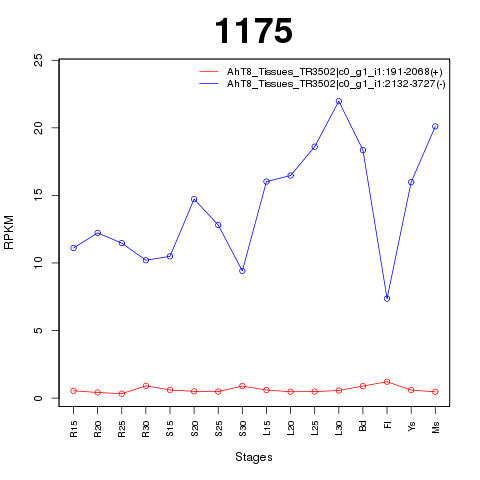

Supplement: S4 Dataset — (ZIP) [file pone.0180528.s009.zip › chimeras_581_PNGs/1175.AhT8_Tissues_TR3502_c0_g1_i1.rpkm.png]

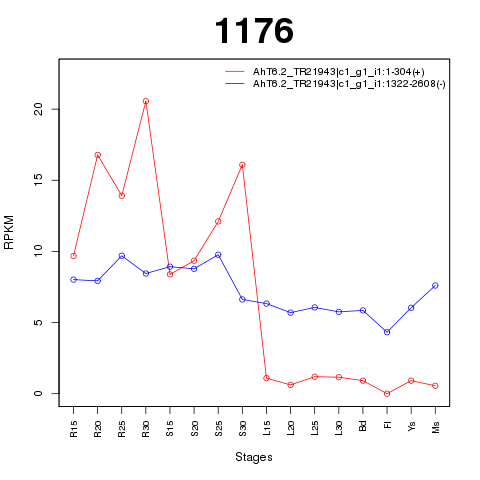

Supplement: S4 Dataset — (ZIP) [file pone.0180528.s009.zip › chimeras_581_PNGs/1176.AhT6.2_TR21943_c1_g1_i1.rpkm.png]

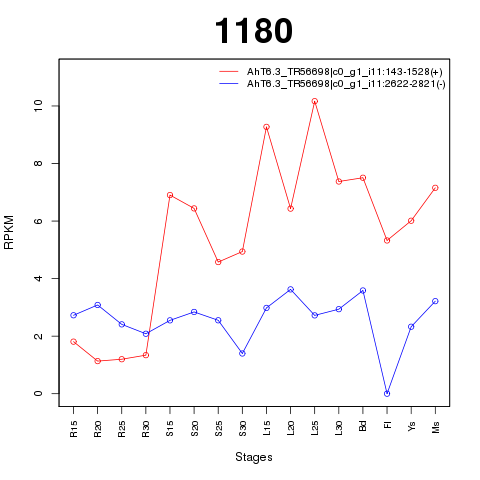

Supplement: S4 Dataset — (ZIP) [file pone.0180528.s009.zip › chimeras_581_PNGs/1180.AhT6.3_TR56698_c0_g1_i11.rpkm.png]

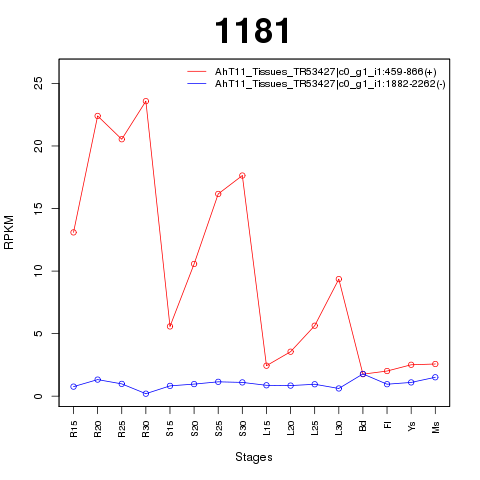

Supplement: S4 Dataset — (ZIP) [file pone.0180528.s009.zip › chimeras_581_PNGs/1181.AhT11_Tissues_TR53427_c0_g1_i1.rpkm.png]

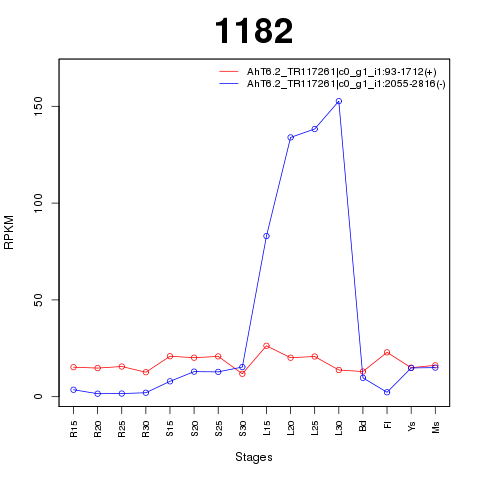

Supplement: S4 Dataset — (ZIP) [file pone.0180528.s009.zip › chimeras_581_PNGs/1182.AhT6.2_TR117261_c0_g1_i1.rpkm.png]

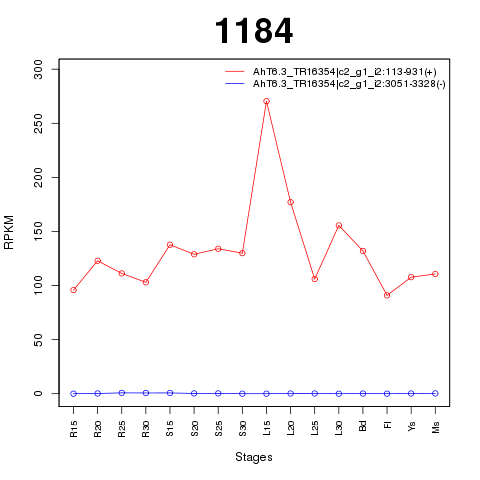

Supplement: S4 Dataset — (ZIP) [file pone.0180528.s009.zip › chimeras_581_PNGs/1184.AhT6.3_TR16354_c2_g1_i2.rpkm.png]

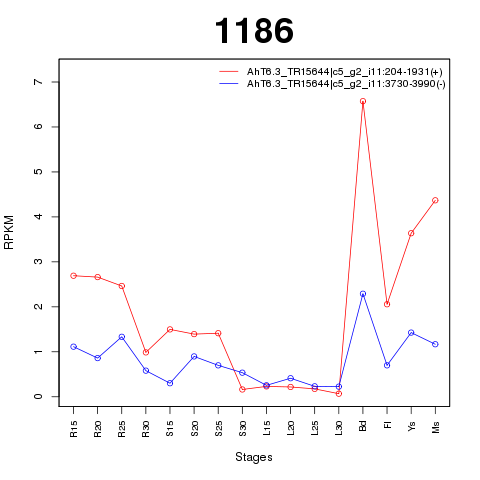

Supplement: S4 Dataset — (ZIP) [file pone.0180528.s009.zip › chimeras_581_PNGs/1186.AhT6.3_TR15644_c5_g2_i11.rpkm.png]

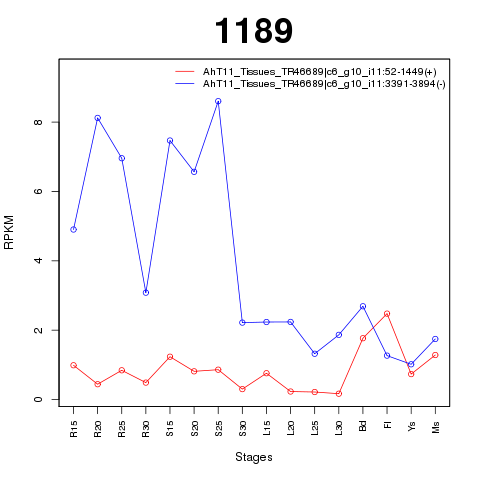

Supplement: S4 Dataset — (ZIP) [file pone.0180528.s009.zip › chimeras_581_PNGs/1189.AhT11_Tissues_TR46689_c6_g10_i11.rpkm.png]

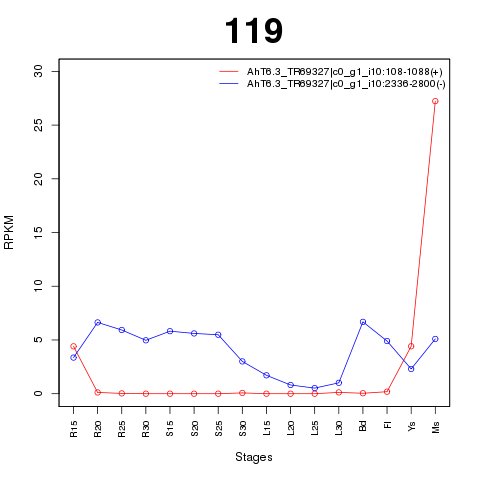

Supplement: S4 Dataset — (ZIP) [file pone.0180528.s009.zip › chimeras_581_PNGs/119.AhT6.3_TR69327_c0_g1_i10.rpkm.png]

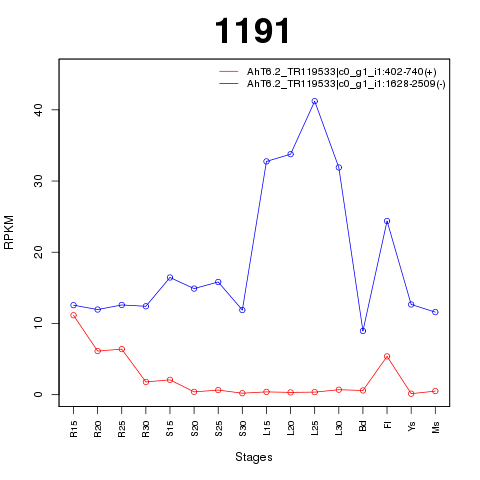

Supplement: S4 Dataset — (ZIP) [file pone.0180528.s009.zip › chimeras_581_PNGs/1191.AhT6.2_TR119533_c0_g1_i1.rpkm.png]

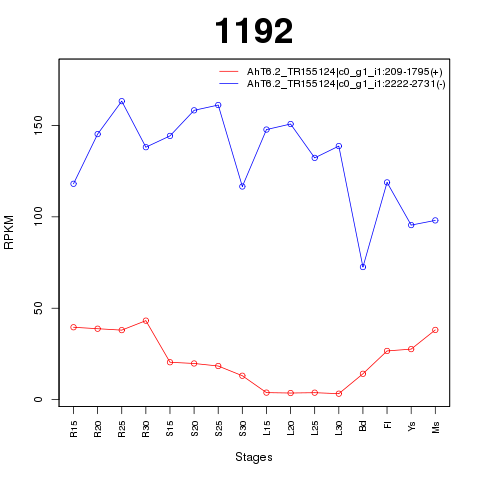

Supplement: S4 Dataset — (ZIP) [file pone.0180528.s009.zip › chimeras_581_PNGs/1192.AhT6.2_TR155124_c0_g1_i1.rpkm.png]

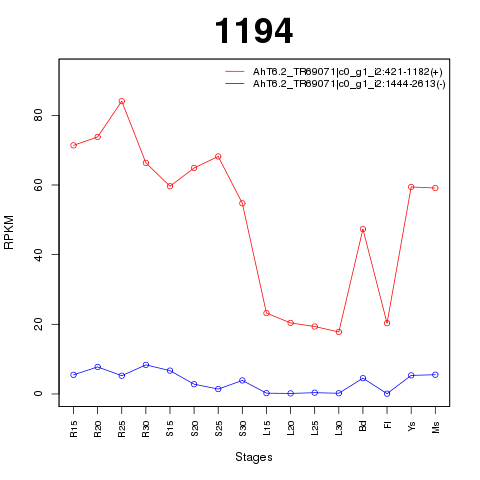

Supplement: S4 Dataset — (ZIP) [file pone.0180528.s009.zip › chimeras_581_PNGs/1194.AhT6.2_TR69071_c0_g1_i2.rpkm.png]

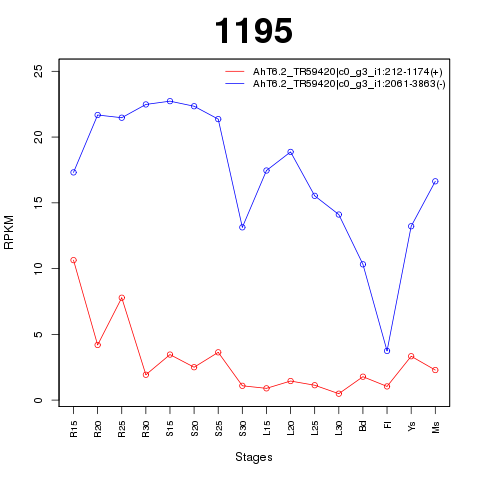

Supplement: S4 Dataset — (ZIP) [file pone.0180528.s009.zip › chimeras_581_PNGs/1195.AhT6.2_TR59420_c0_g3_i1.rpkm.png]

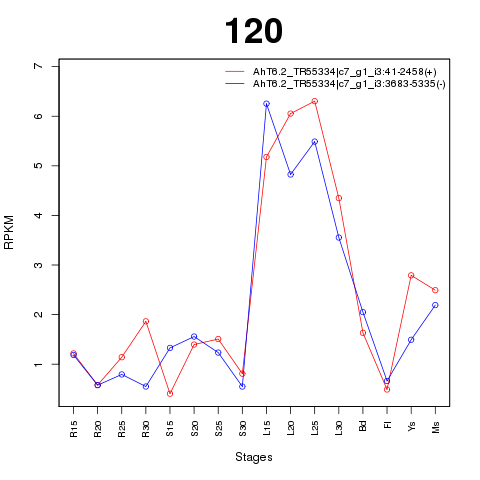

Supplement: S4 Dataset — (ZIP) [file pone.0180528.s009.zip › chimeras_581_PNGs/120.AhT6.2_TR55334_c7_g1_i3.rpkm.png]

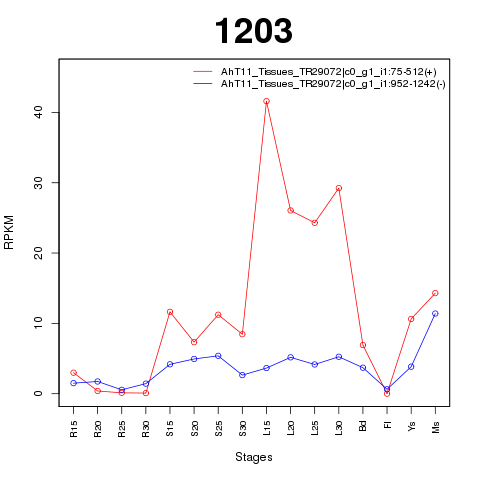

Supplement: S4 Dataset — (ZIP) [file pone.0180528.s009.zip › chimeras_581_PNGs/1203.AhT11_Tissues_TR29072_c0_g1_i1.rpkm.png]

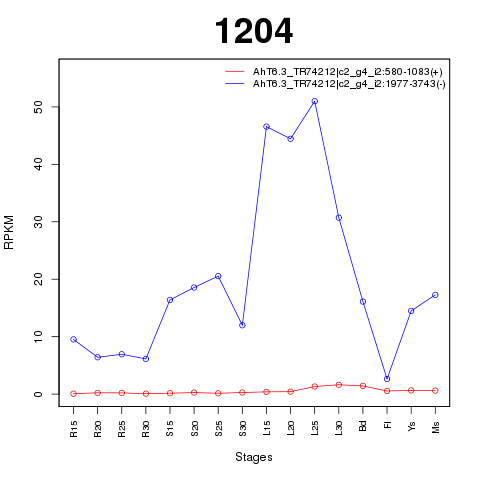

Supplement: S4 Dataset — (ZIP) [file pone.0180528.s009.zip › chimeras_581_PNGs/1204.AhT6.3_TR74212_c2_g4_i2.rpkm.png]

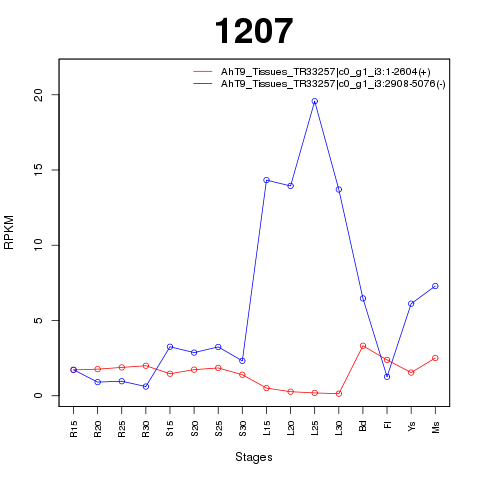

Supplement: S4 Dataset — (ZIP) [file pone.0180528.s009.zip › chimeras_581_PNGs/1207.AhT9_Tissues_TR33257_c0_g1_i3.rpkm.png]

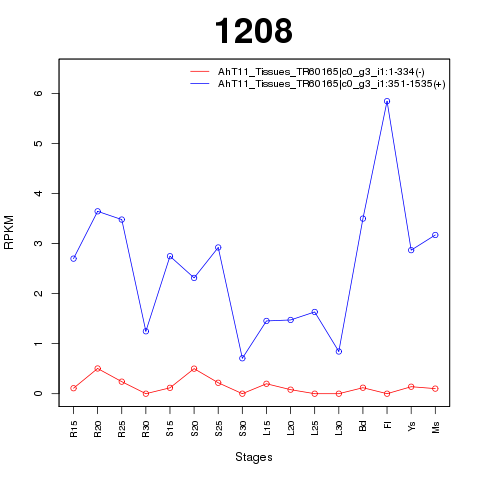

Supplement: S4 Dataset — (ZIP) [file pone.0180528.s009.zip › chimeras_581_PNGs/1208.AhT11_Tissues_TR60165_c0_g3_i1.rpkm.png]

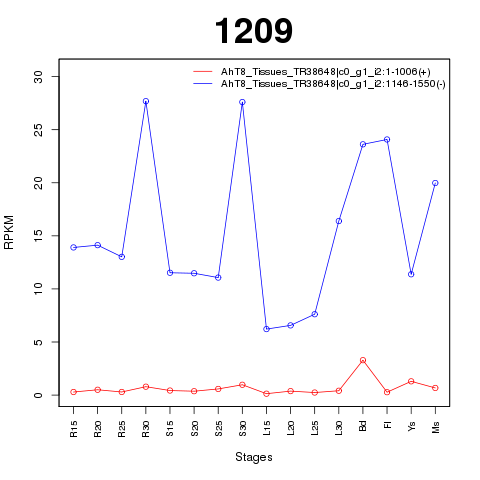

Supplement: S4 Dataset — (ZIP) [file pone.0180528.s009.zip › chimeras_581_PNGs/1209.AhT8_Tissues_TR38648_c0_g1_i2.rpkm.png]

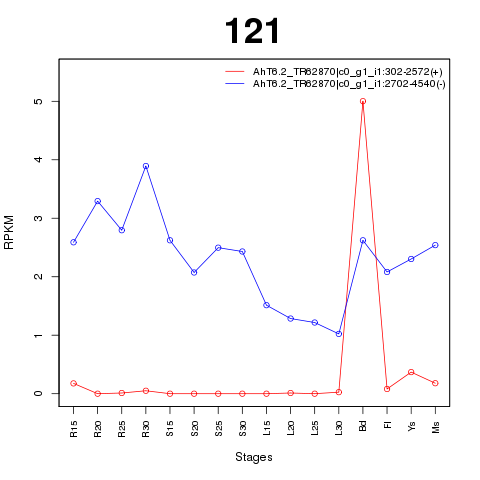

Supplement: S4 Dataset — (ZIP) [file pone.0180528.s009.zip › chimeras_581_PNGs/121.AhT6.2_TR62870_c0_g1_i1.rpkm.png]
