# Supplementary material for: Differential genomic arrangements in Caryophyllales through deep transcriptome sequencing of A. hypochondriacus
Source: PLoS One. 2017 Aug 7;12(8):e0180528. doi: 10.1371/journal.pone.0180528 (PMC5546567; doi:10.1371/journal.pone.0180528)
Supplement: S1 Table — *Libraries sequenced previously [6]. (DOCX) [file pone.0180528.s004.docx]

| **cDNA Lib #** | **Condition sequenced** | **# QCed Read pairs** | **Rd Len**  **(bp)** | **Illumina Platform** |
| --- | --- | --- | --- | --- |
| 1* | Shoot (leaves and stem) from 15 days old plants from batch 1 | 18132001 | 72 | GA IIx |
| 2* | Shoot (leaves and stem) from 25 days old plants from batch 1 | 16428322 | 72 | GA IIx |
| 3* | Shoot (leaves and stem) from 30 days old plants from batch 1 | 14167601 | 72 | GA IIx |
| 4* | Mature seeds from plants from batch 1 | 11210075 | 72 | GA IIx |
| 5 | Leaves from 15 days old plants from batches 1, 2 and 3 | 15092464 | 100 | Hiseq 2500 |
| 6 | Stem from 15 days old plants from batches 1, 2 and 3 | 25499894 | 100 | Hiseq 2500 |
| 7 | Root from 15 days old plants from batches 1, 2 and 3 | 27519831 | 100 | Hiseq 2500 |
| 8 | Leaves from 20 days old plants from batches 1, 2 and 3 | 37237718 | 100 | Hiseq 2500 |
| 9 | Stem from 20 days old plants from batches 1, 2 and 3 | 29912199 | 100 | Hiseq 2500 |
| 10 | Root from 20 days old plants from batches 1, 2 and 3 | 17833896 | 100 | Hiseq 2500 |
| 11 | Leaves from 25 days old plants from batches 1, 2 and 3 | 16533796 | 100 | Hiseq 2500 |
| 12 | Stem from 25 days old plants from batches 1, 2 and 3 | 27440240 | 100 | Hiseq 2500 |
| 13 | Root from 25 days old plants from batches 1, 2 and 3 | 37353354 | 100 | Hiseq 2500 |
| 14 | Leaves from 30 days old plants from batches 1, 2 and 3 | 17022005 | 100 | Hiseq 2500 |
| 15 | Stem from 30 days old plants from batches 1, 2 and 3 | 14313117 | 100 | Hiseq 2500 |
| 16 | Root from 30 days old plants from batches 1, 2 and 3 | 26399133 | 100 | Hiseq 2500 |
| 17 | Inflorescence with flower buds from batches 1, 2 and 3 | 25088891 | 100 | Hiseq 2500 |
| 18 | Inflorescence with flowers from batches 1, 2 and 3 | 10973325 | 100 | Hiseq 2500 |
| 19 | Inflorescence with young seeds from batches 1, 2 and 3 | 21482785 | 100 | Hiseq 2500 |
| 20 | Mature seeds from batches 1, 2 and 3 | 29540977 | 100 | Hiseq 2500 |
