# Supplementary material for: Differential genomic arrangements in Caryophyllales through deep transcriptome sequencing of A. hypochondriacus
Source: PLoS One. 2017 Aug 7;12(8):e0180528. doi: 10.1371/journal.pone.0180528 (PMC5546567; doi:10.1371/journal.pone.0180528)
Supplement: S2 Table — (DOCX) [file pone.0180528.s005.docx]

| **Assembly ID** | **Description** |
| --- | --- |
| oases_k21 - oases_k65 | 12 assemblies of libraries 1-4 listed in Table 1 using Oases with k-mers in steps of four ranging from 21 to 65 |
| soap_k21 - soap_k65 | 12 assemblies of libraries 1-4 listed in Table 1 using SOAPdenovo-Trans with k-mers in steps of four ranging from 21 to 65 |
| AhT5 | Assembly of libraries 1-4 listed in Table 1 using Trinity r20130216 |
| AhT6.2 | Assembly of all 20 transcriptomes pooled together using Trinity 2.0.5 |
| AhT6.3 | Assembly of all 20 transcriptomes pooled together and digitally normalized using Trinity 2.0.5 |
| AhT7 | Assembly of shoot transcriptomes from libraries 1-3 and 17-18 listed in Table 1 using Trinity 2.0.5 |
| AhT8 | Assembly of leaf transcriptomes from libraries 5, 8, 11 and 14 listed in Table 1 using Trinity 2.0.5 |
| AhT9 | Assembly of stem transcriptomes from libraries 6, 9, 12 and 15 listed in Table 1 using Trinity 2.0.5 |
| AhT10 | Assembly of root transcriptomes from libraries 7, 10, 13 and 16 listed in Table 1 using Trinity 2.0.5 |
| AhT11 | Assembly of seed transcriptomes from libraries 4, 19 and 20 listed in Table 1 using Trinity 2.0.5 |
