# Supplementary figures and images for: Differential genomic arrangements in Caryophyllales through deep transcriptome sequencing of A. hypochondriacus (part 3 of 3)
Source: PLoS One. 2017 Aug 7;12(8):e0180528. doi: 10.1371/journal.pone.0180528 (PMC5546567; doi:10.1371/journal.pone.0180528)

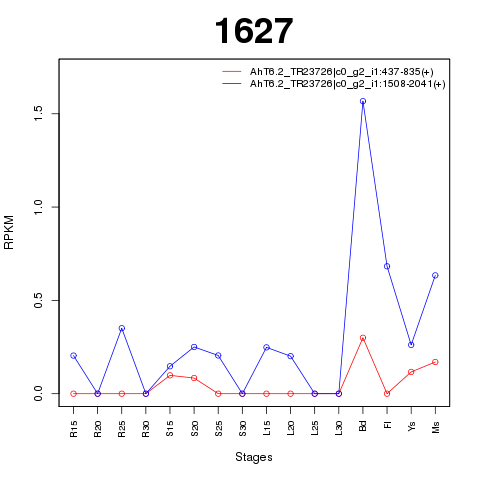

Supplement: S4 Dataset — (ZIP) [file pone.0180528.s009.zip › chimeras_581_PNGs/1627.AhT6.2_TR23726_c0_g2_i1.rpkm.png]

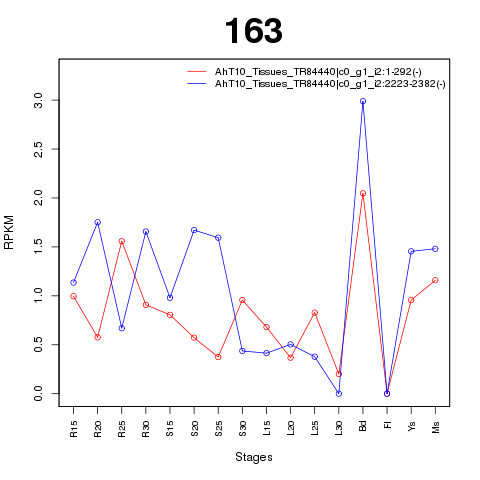

Supplement: S4 Dataset — (ZIP) [file pone.0180528.s009.zip › chimeras_581_PNGs/163.AhT10_Tissues_TR84440_c0_g1_i2.rpkm.png]

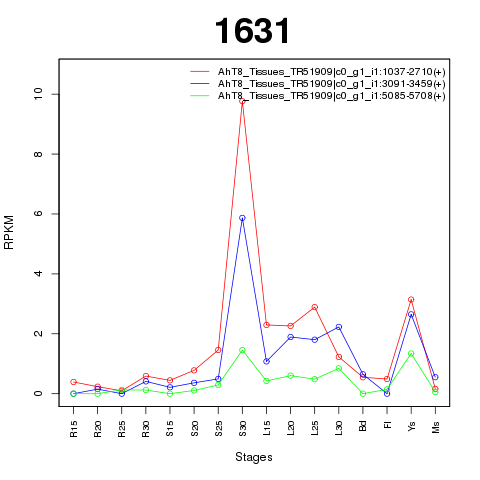

Supplement: S4 Dataset — (ZIP) [file pone.0180528.s009.zip › chimeras_581_PNGs/1631.AhT8_Tissues_TR51909_c0_g1_i1.rpkm.png]
