# Supplementary figures and images for: Differential genomic arrangements in Caryophyllales through deep transcriptome sequencing of A. hypochondriacus (part 2 of 3)
Source: PLoS One. 2017 Aug 7;12(8):e0180528. doi: 10.1371/journal.pone.0180528 (PMC5546567; doi:10.1371/journal.pone.0180528)

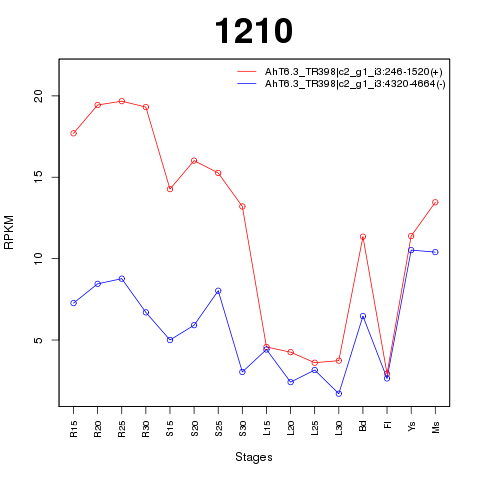

Supplement: S4 Dataset — (ZIP) [file pone.0180528.s009.zip › chimeras_581_PNGs/1210.AhT6.3_TR398_c2_g1_i3.rpkm.png]

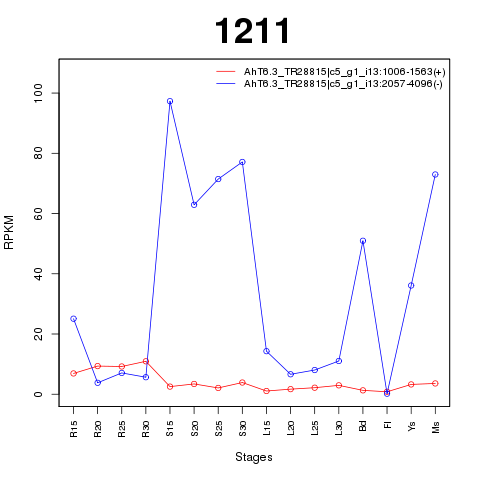

Supplement: S4 Dataset — (ZIP) [file pone.0180528.s009.zip › chimeras_581_PNGs/1211.AhT6.3_TR28815_c5_g1_i13.rpkm.png]

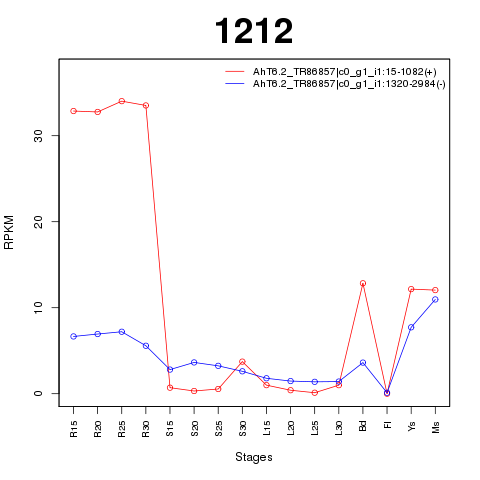

Supplement: S4 Dataset — (ZIP) [file pone.0180528.s009.zip › chimeras_581_PNGs/1212.AhT6.2_TR86857_c0_g1_i1.rpkm.png]

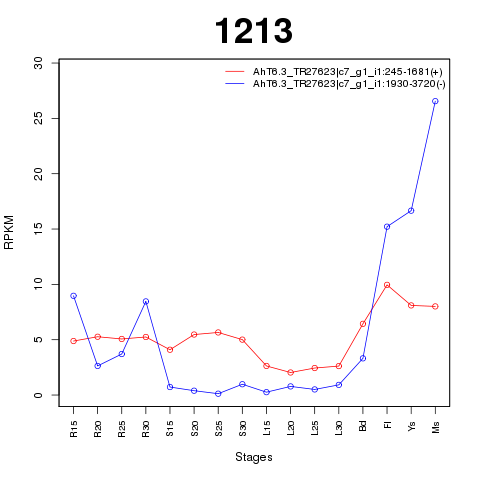

Supplement: S4 Dataset — (ZIP) [file pone.0180528.s009.zip › chimeras_581_PNGs/1213.AhT6.3_TR27623_c7_g1_i1.rpkm.png]

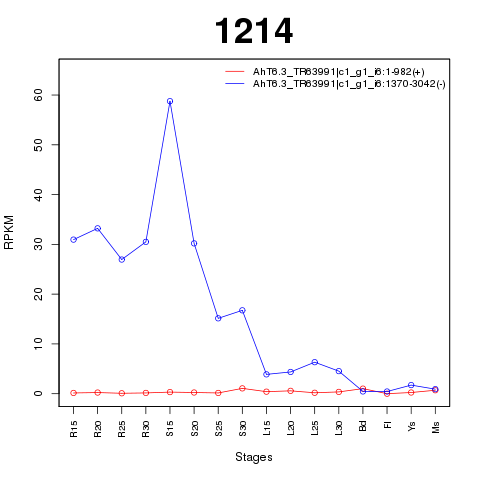

Supplement: S4 Dataset — (ZIP) [file pone.0180528.s009.zip › chimeras_581_PNGs/1214.AhT6.3_TR63991_c1_g1_i6.rpkm.png]

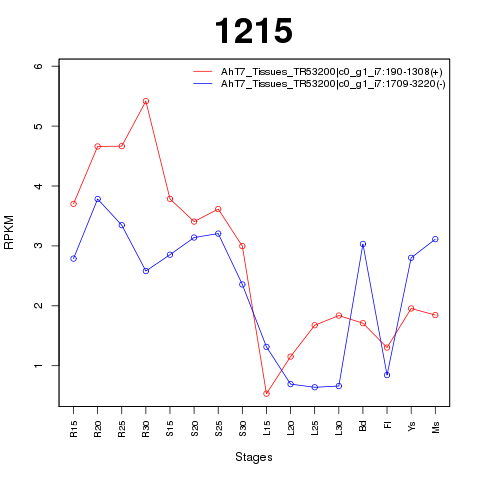

Supplement: S4 Dataset — (ZIP) [file pone.0180528.s009.zip › chimeras_581_PNGs/1215.AhT7_Tissues_TR53200_c0_g1_i7.rpkm.png]

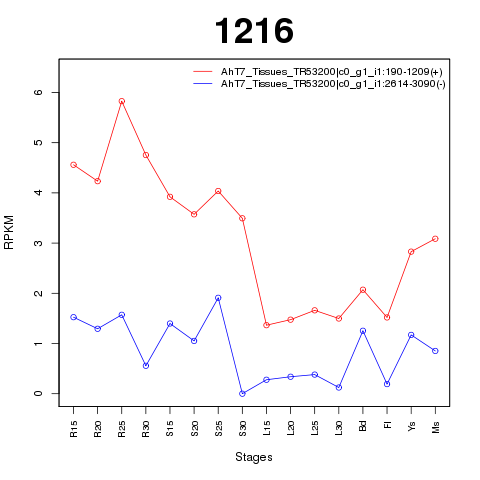

Supplement: S4 Dataset — (ZIP) [file pone.0180528.s009.zip › chimeras_581_PNGs/1216.AhT7_Tissues_TR53200_c0_g1_i1.rpkm.png]

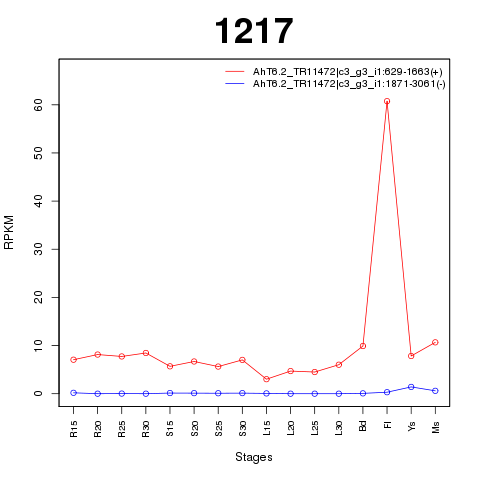

Supplement: S4 Dataset — (ZIP) [file pone.0180528.s009.zip › chimeras_581_PNGs/1217.AhT6.2_TR11472_c3_g3_i1.rpkm.png]

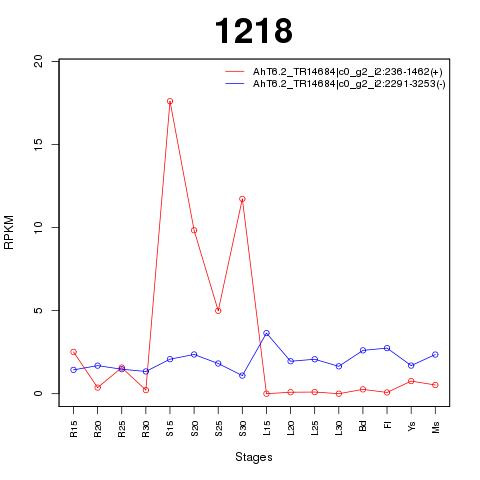

Supplement: S4 Dataset — (ZIP) [file pone.0180528.s009.zip › chimeras_581_PNGs/1218.AhT6.2_TR14684_c0_g2_i2.rpkm.png]

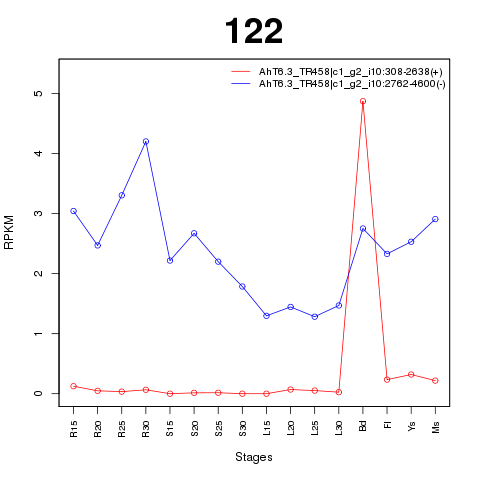

Supplement: S4 Dataset — (ZIP) [file pone.0180528.s009.zip › chimeras_581_PNGs/122.AhT6.3_TR458_c1_g2_i10.rpkm.png]

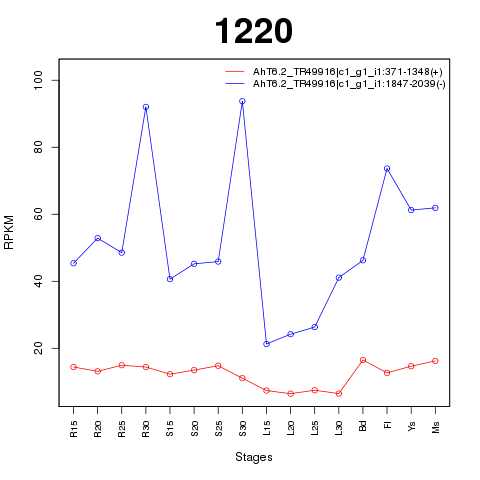

Supplement: S4 Dataset — (ZIP) [file pone.0180528.s009.zip › chimeras_581_PNGs/1220.AhT6.2_TR49916_c1_g1_i1.rpkm.png]

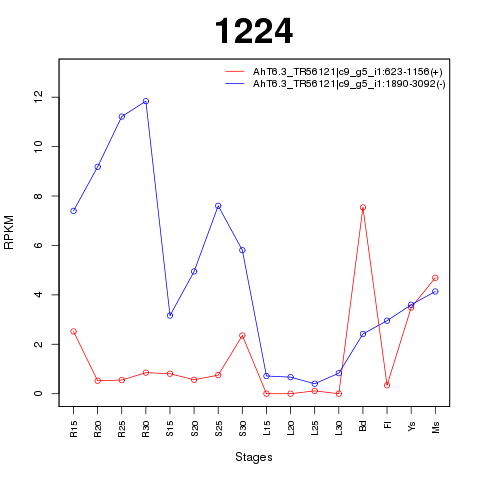

Supplement: S4 Dataset — (ZIP) [file pone.0180528.s009.zip › chimeras_581_PNGs/1224.AhT6.3_TR56121_c9_g5_i1.rpkm.png]

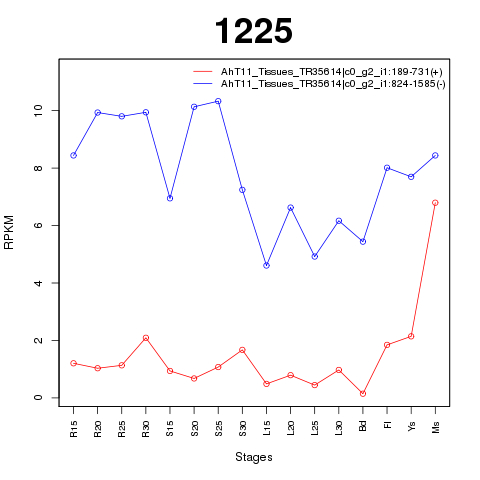

Supplement: S4 Dataset — (ZIP) [file pone.0180528.s009.zip › chimeras_581_PNGs/1225.AhT11_Tissues_TR35614_c0_g2_i1.rpkm.png]

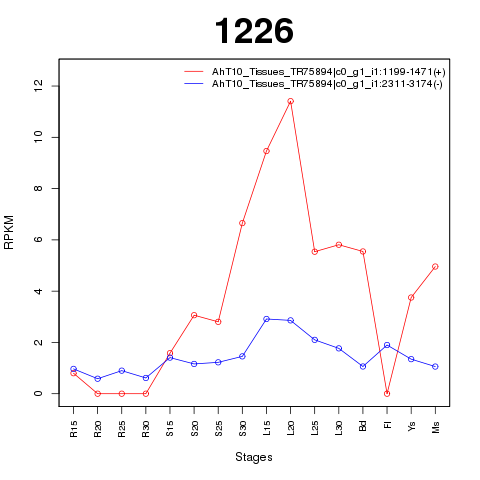

Supplement: S4 Dataset — (ZIP) [file pone.0180528.s009.zip › chimeras_581_PNGs/1226.AhT10_Tissues_TR75894_c0_g1_i1.rpkm.png]

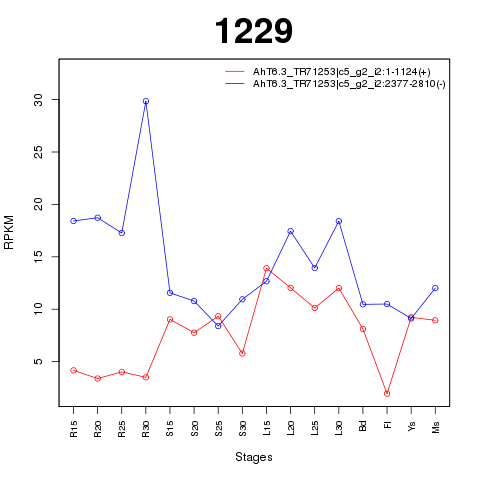

Supplement: S4 Dataset — (ZIP) [file pone.0180528.s009.zip › chimeras_581_PNGs/1229.AhT6.3_TR71253_c5_g2_i2.rpkm.png]

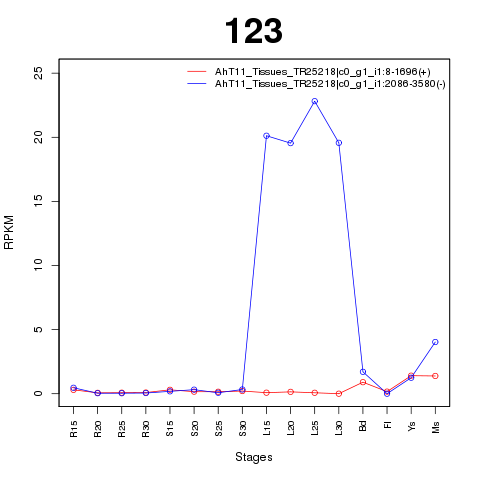

Supplement: S4 Dataset — (ZIP) [file pone.0180528.s009.zip › chimeras_581_PNGs/123.AhT11_Tissues_TR25218_c0_g1_i1.rpkm.png]

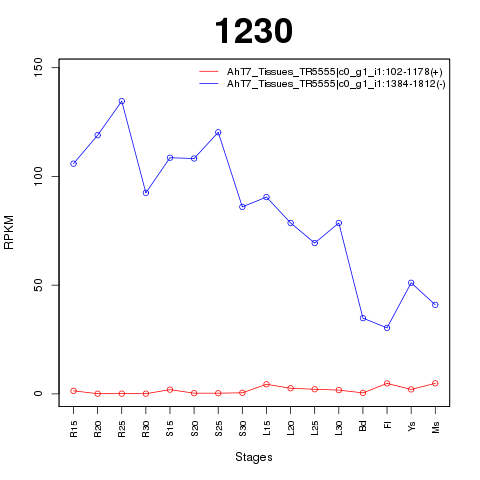

Supplement: S4 Dataset — (ZIP) [file pone.0180528.s009.zip › chimeras_581_PNGs/1230.AhT7_Tissues_TR5555_c0_g1_i1.rpkm.png]

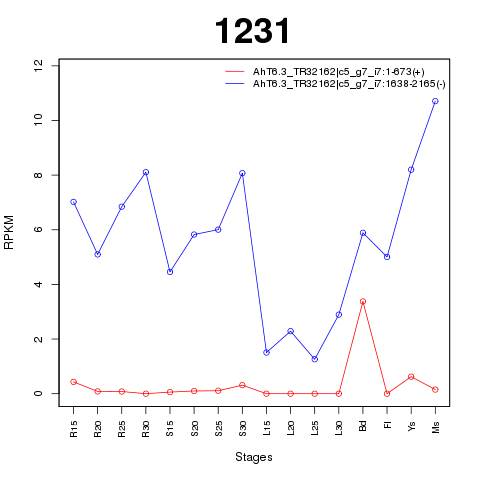

Supplement: S4 Dataset — (ZIP) [file pone.0180528.s009.zip › chimeras_581_PNGs/1231.AhT6.3_TR32162_c5_g7_i7.rpkm.png]

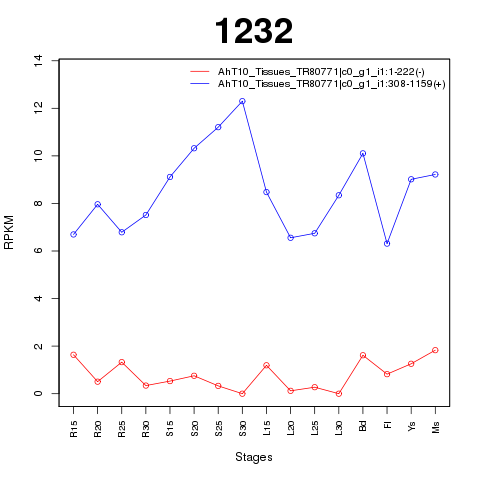

Supplement: S4 Dataset — (ZIP) [file pone.0180528.s009.zip › chimeras_581_PNGs/1232.AhT10_Tissues_TR80771_c0_g1_i1.rpkm.png]

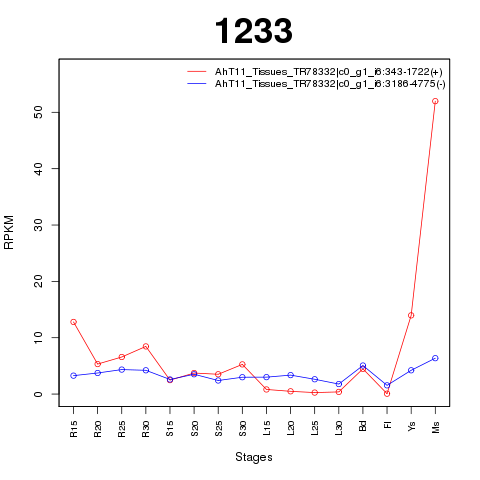

Supplement: S4 Dataset — (ZIP) [file pone.0180528.s009.zip › chimeras_581_PNGs/1233.AhT11_Tissues_TR78332_c0_g1_i6.rpkm.png]

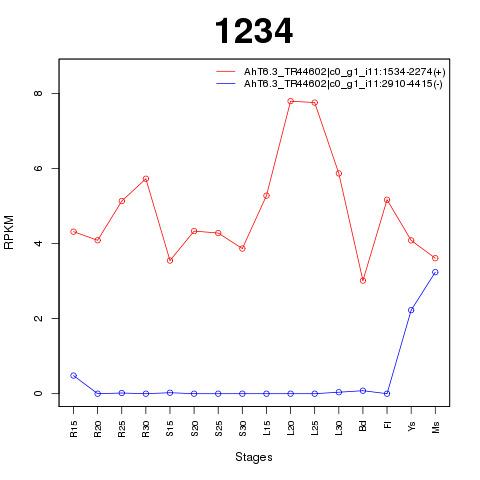

Supplement: S4 Dataset — (ZIP) [file pone.0180528.s009.zip › chimeras_581_PNGs/1234.AhT6.3_TR44602_c0_g1_i11.rpkm.png]

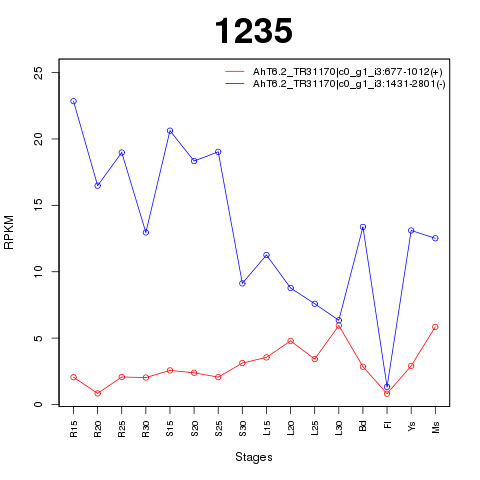

Supplement: S4 Dataset — (ZIP) [file pone.0180528.s009.zip › chimeras_581_PNGs/1235.AhT6.2_TR31170_c0_g1_i3.rpkm.png]

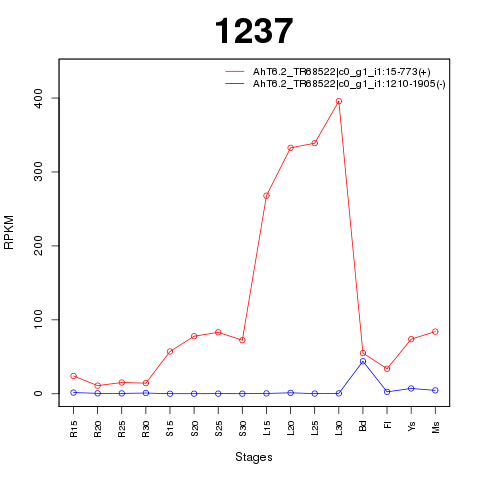

Supplement: S4 Dataset — (ZIP) [file pone.0180528.s009.zip › chimeras_581_PNGs/1237.AhT6.2_TR68522_c0_g1_i1.rpkm.png]

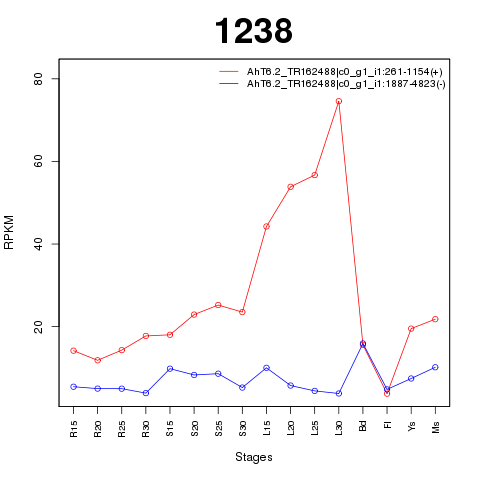

Supplement: S4 Dataset — (ZIP) [file pone.0180528.s009.zip › chimeras_581_PNGs/1238.AhT6.2_TR162488_c0_g1_i1.rpkm.png]

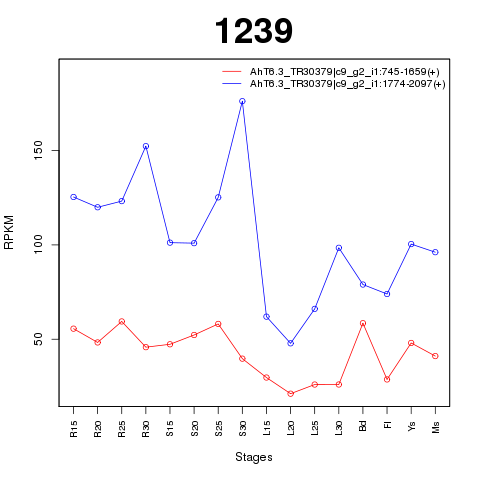

Supplement: S4 Dataset — (ZIP) [file pone.0180528.s009.zip › chimeras_581_PNGs/1239.AhT6.3_TR30379_c9_g2_i1.rpkm.png]

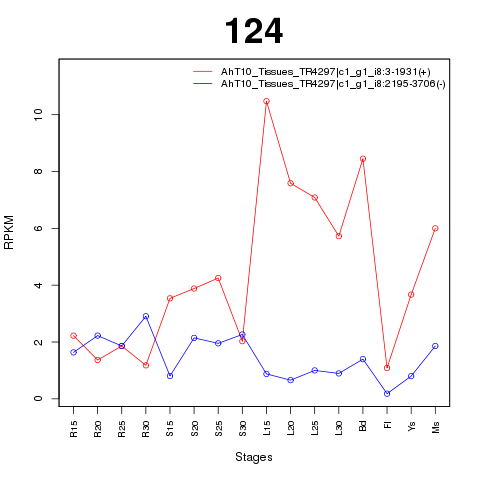

Supplement: S4 Dataset — (ZIP) [file pone.0180528.s009.zip › chimeras_581_PNGs/124.AhT10_Tissues_TR4297_c1_g1_i8.rpkm.png]

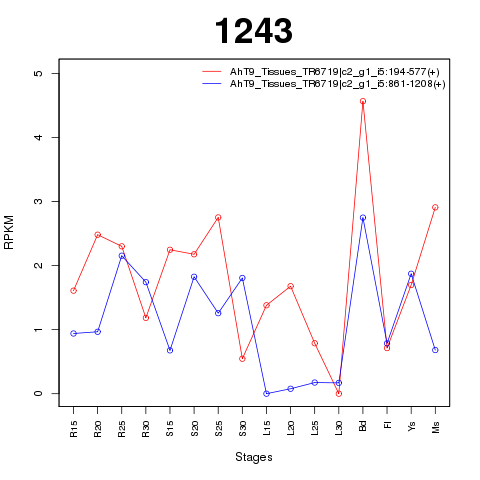

Supplement: S4 Dataset — (ZIP) [file pone.0180528.s009.zip › chimeras_581_PNGs/1243.AhT9_Tissues_TR6719_c2_g1_i5.rpkm.png]

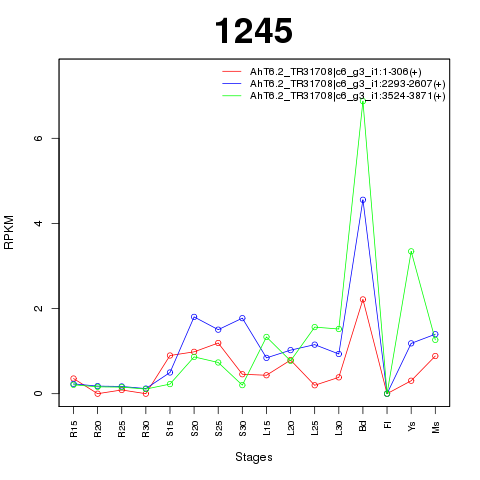

Supplement: S4 Dataset — (ZIP) [file pone.0180528.s009.zip › chimeras_581_PNGs/1245.AhT6.2_TR31708_c6_g3_i1.rpkm.png]

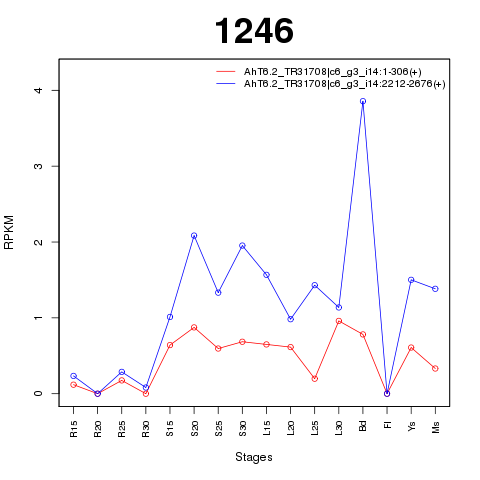

Supplement: S4 Dataset — (ZIP) [file pone.0180528.s009.zip › chimeras_581_PNGs/1246.AhT6.2_TR31708_c6_g3_i14.rpkm.png]

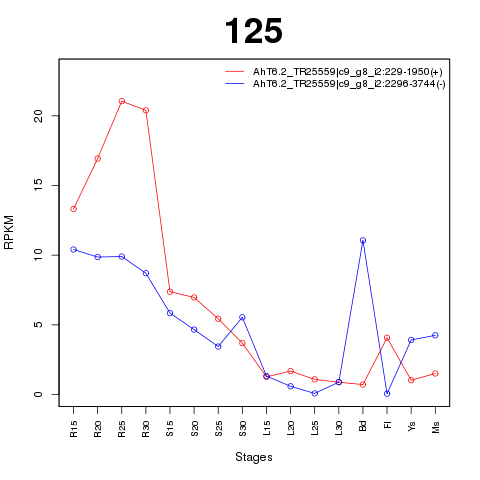

Supplement: S4 Dataset — (ZIP) [file pone.0180528.s009.zip › chimeras_581_PNGs/125.AhT6.2_TR25559_c9_g8_i2.rpkm.png]

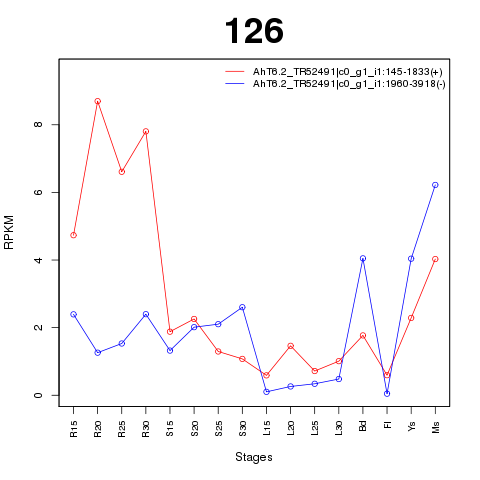

Supplement: S4 Dataset — (ZIP) [file pone.0180528.s009.zip › chimeras_581_PNGs/126.AhT6.2_TR52491_c0_g1_i1.rpkm.png]

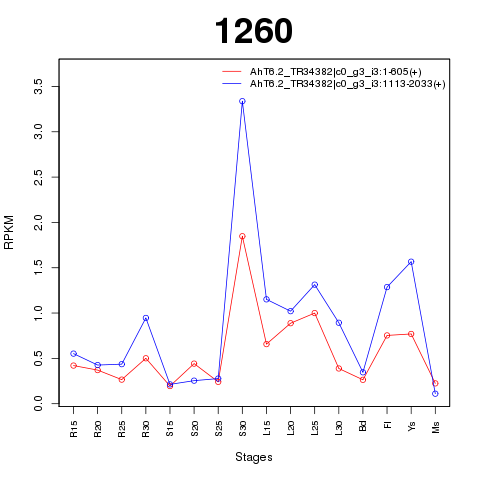

Supplement: S4 Dataset — (ZIP) [file pone.0180528.s009.zip › chimeras_581_PNGs/1260.AhT6.2_TR34382_c0_g3_i3.rpkm.png]

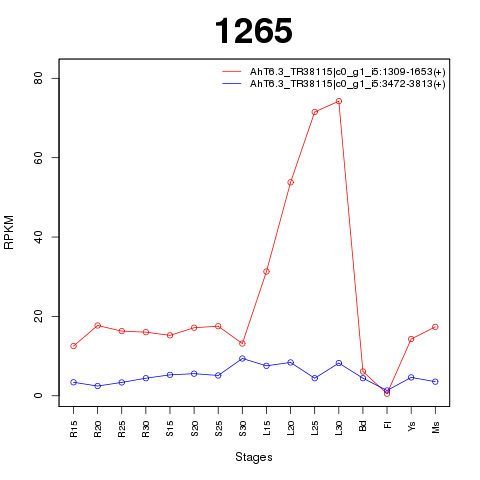

Supplement: S4 Dataset — (ZIP) [file pone.0180528.s009.zip › chimeras_581_PNGs/1265.AhT6.3_TR38115_c0_g1_i5.rpkm.png]

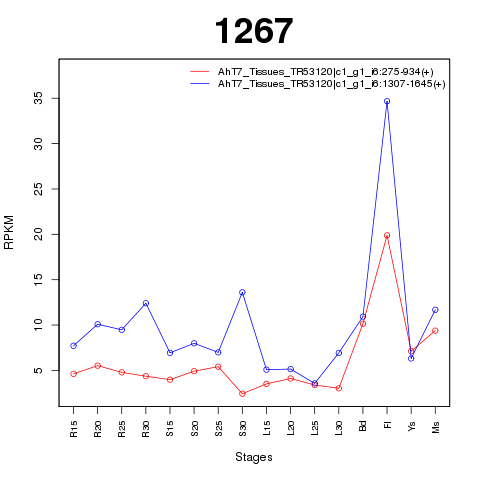

Supplement: S4 Dataset — (ZIP) [file pone.0180528.s009.zip › chimeras_581_PNGs/1267.AhT7_Tissues_TR53120_c1_g1_i6.rpkm.png]

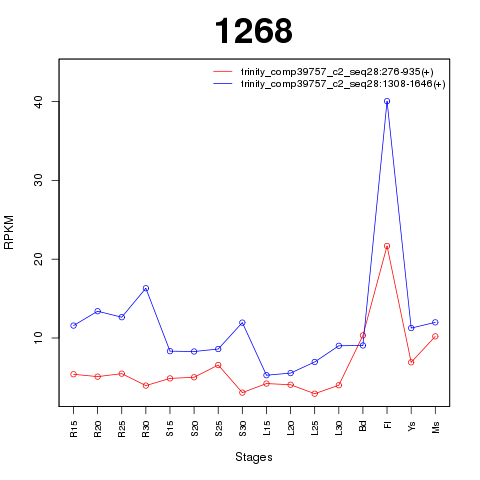

Supplement: S4 Dataset — (ZIP) [file pone.0180528.s009.zip › chimeras_581_PNGs/1268.trinity_comp39757_c2_seq28.rpkm.png]

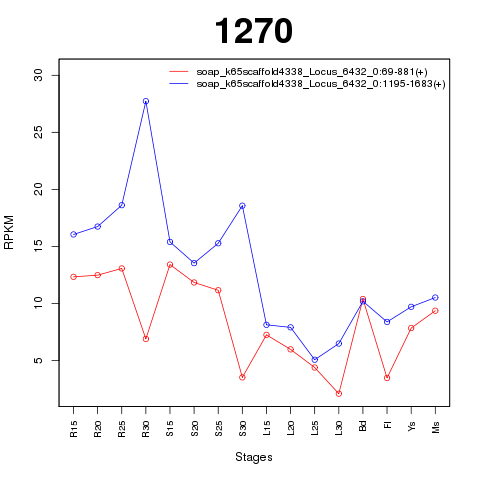

Supplement: S4 Dataset — (ZIP) [file pone.0180528.s009.zip › chimeras_581_PNGs/1270.soap_k65scaffold4338_Locus_6432_0.rpkm.png]

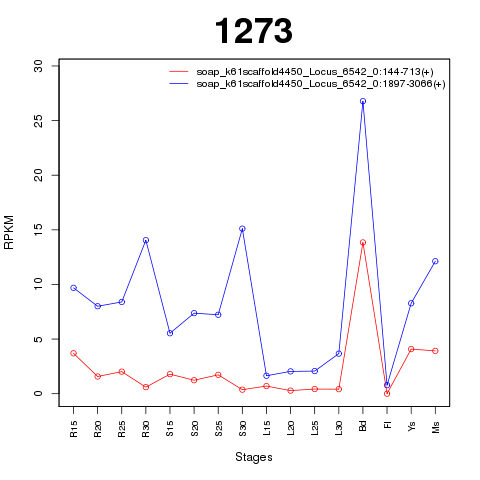

Supplement: S4 Dataset — (ZIP) [file pone.0180528.s009.zip › chimeras_581_PNGs/1273.soap_k61scaffold4450_Locus_6542_0.rpkm.png]

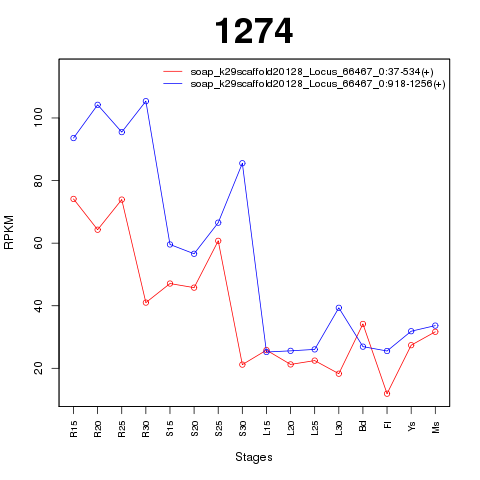

Supplement: S4 Dataset — (ZIP) [file pone.0180528.s009.zip › chimeras_581_PNGs/1274.soap_k29scaffold20128_Locus_66467_0.rpkm.png]

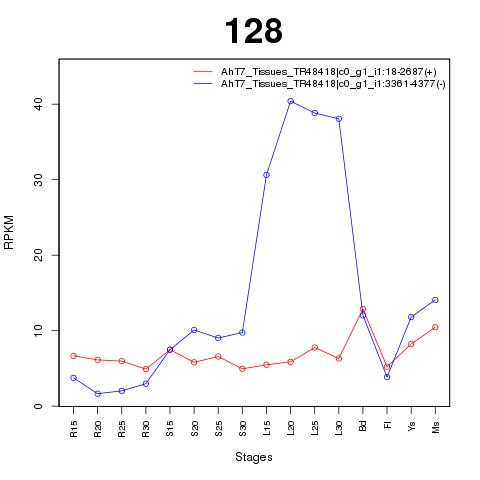

Supplement: S4 Dataset — (ZIP) [file pone.0180528.s009.zip › chimeras_581_PNGs/128.AhT7_Tissues_TR48418_c0_g1_i1.rpkm.png]

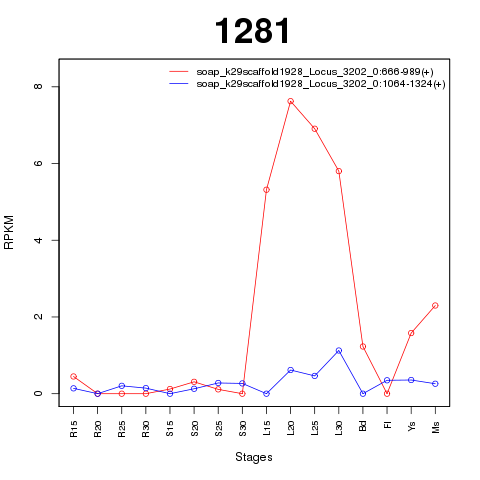

Supplement: S4 Dataset — (ZIP) [file pone.0180528.s009.zip › chimeras_581_PNGs/1281.soap_k29scaffold1928_Locus_3202_0.rpkm.png]

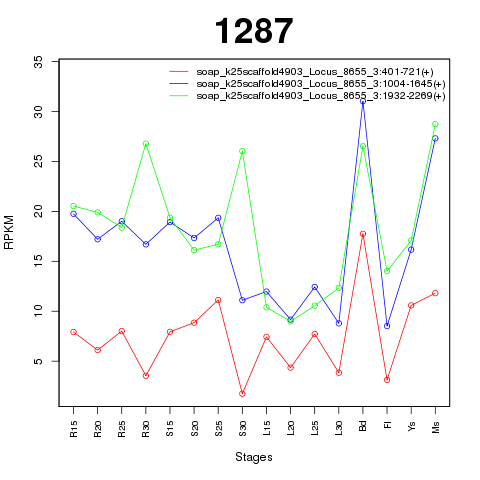

Supplement: S4 Dataset — (ZIP) [file pone.0180528.s009.zip › chimeras_581_PNGs/1287.soap_k25scaffold4903_Locus_8655_3.rpkm.png]

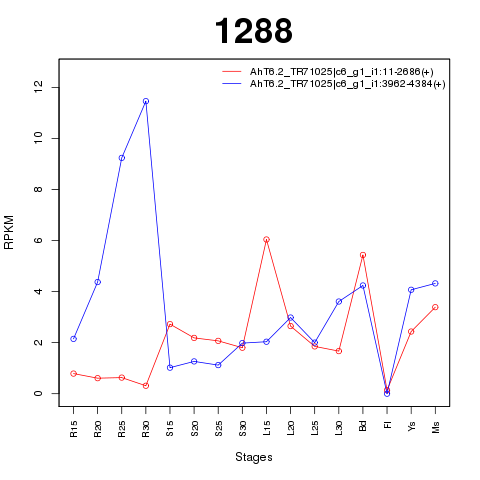

Supplement: S4 Dataset — (ZIP) [file pone.0180528.s009.zip › chimeras_581_PNGs/1288.AhT6.2_TR71025_c6_g1_i1.rpkm.png]

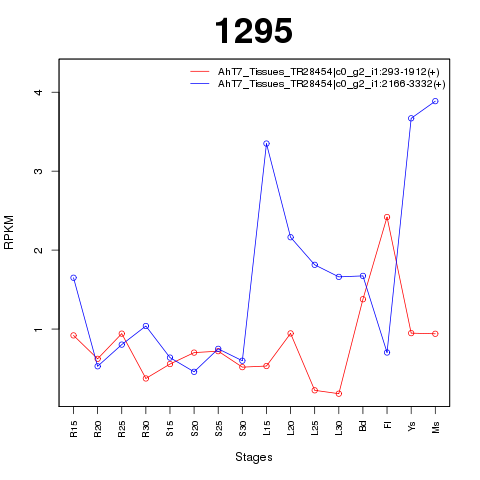

Supplement: S4 Dataset — (ZIP) [file pone.0180528.s009.zip › chimeras_581_PNGs/1295.AhT7_Tissues_TR28454_c0_g2_i1.rpkm.png]

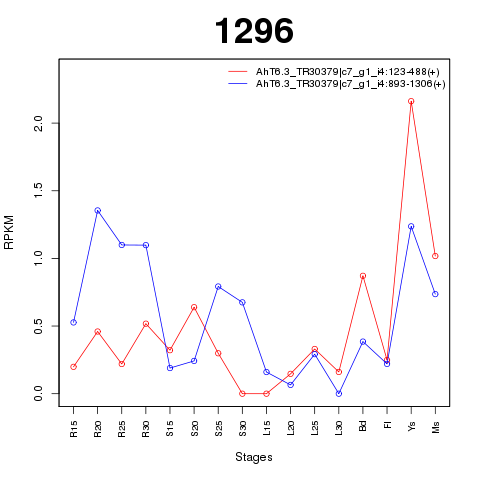

Supplement: S4 Dataset — (ZIP) [file pone.0180528.s009.zip › chimeras_581_PNGs/1296.AhT6.3_TR30379_c7_g1_i4.rpkm.png]

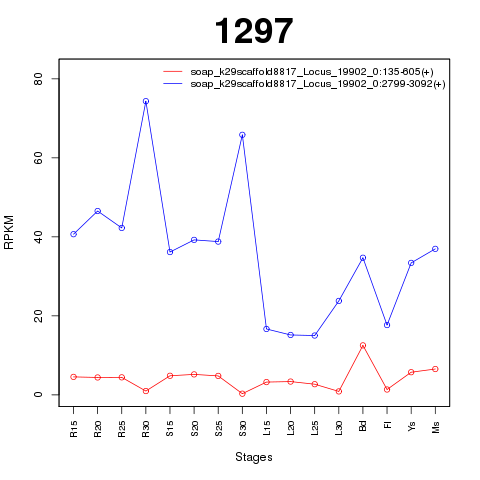

Supplement: S4 Dataset — (ZIP) [file pone.0180528.s009.zip › chimeras_581_PNGs/1297.soap_k29scaffold8817_Locus_19902_0.rpkm.png]

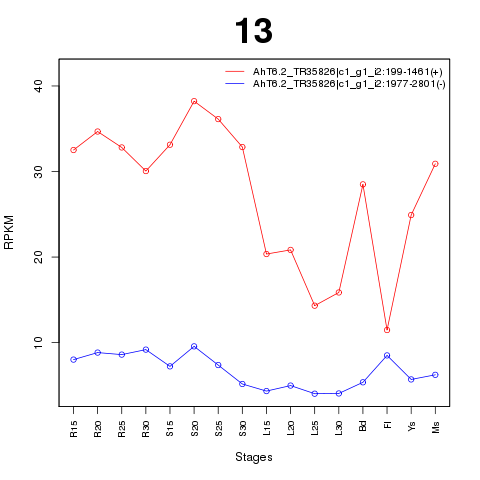

Supplement: S4 Dataset — (ZIP) [file pone.0180528.s009.zip › chimeras_581_PNGs/13.AhT6.2_TR35826_c1_g1_i2.rpkm.png]

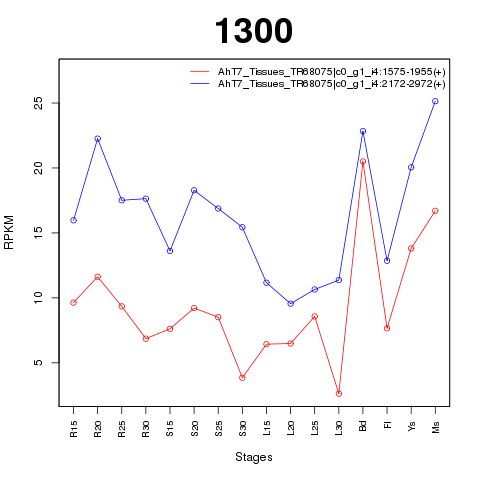

Supplement: S4 Dataset — (ZIP) [file pone.0180528.s009.zip › chimeras_581_PNGs/1300.AhT7_Tissues_TR68075_c0_g1_i4.rpkm.png]

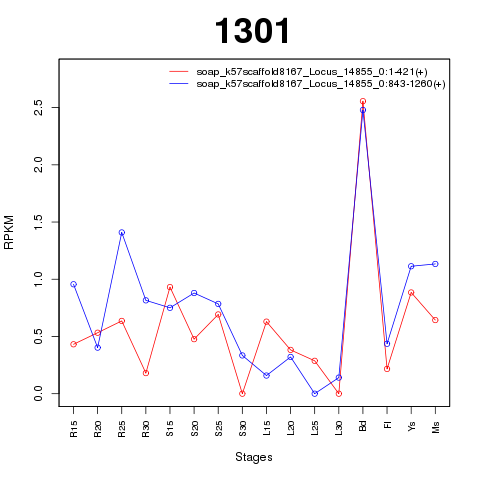

Supplement: S4 Dataset — (ZIP) [file pone.0180528.s009.zip › chimeras_581_PNGs/1301.soap_k57scaffold8167_Locus_14855_0.rpkm.png]

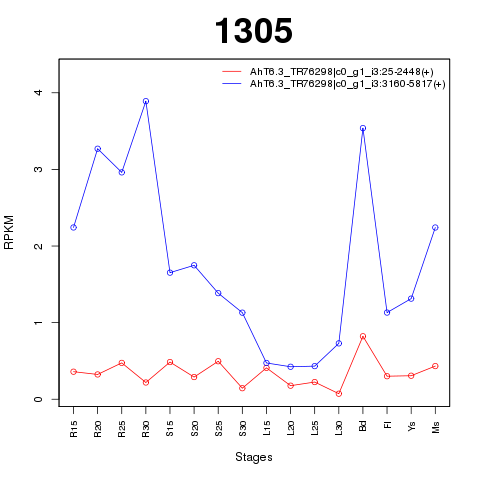

Supplement: S4 Dataset — (ZIP) [file pone.0180528.s009.zip › chimeras_581_PNGs/1305.AhT6.3_TR76298_c0_g1_i3.rpkm.png]

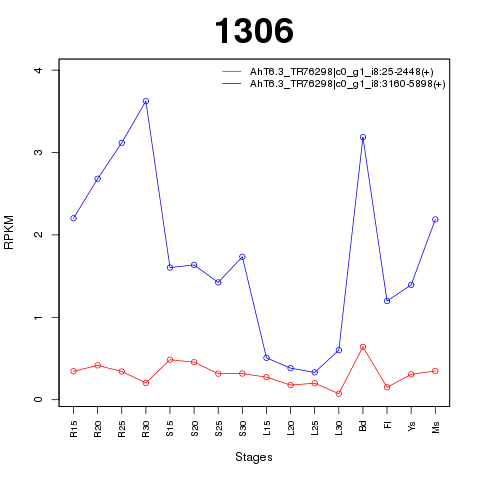

Supplement: S4 Dataset — (ZIP) [file pone.0180528.s009.zip › chimeras_581_PNGs/1306.AhT6.3_TR76298_c0_g1_i8.rpkm.png]

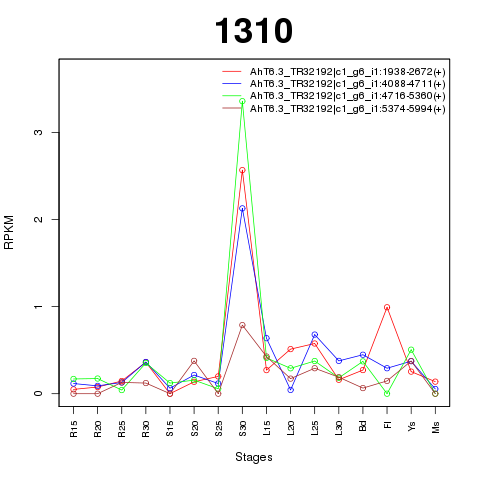

Supplement: S4 Dataset — (ZIP) [file pone.0180528.s009.zip › chimeras_581_PNGs/1310.AhT6.3_TR32192_c1_g6_i1.rpkm.png]

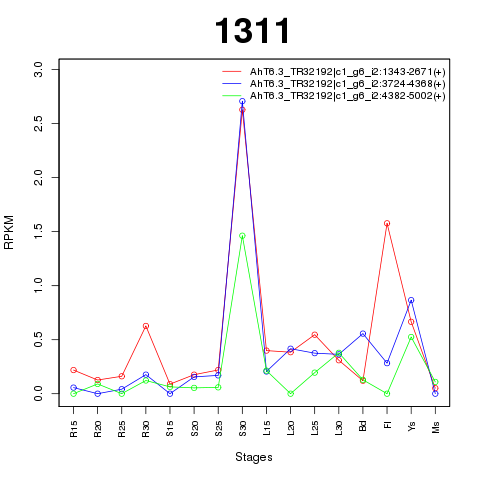

Supplement: S4 Dataset — (ZIP) [file pone.0180528.s009.zip › chimeras_581_PNGs/1311.AhT6.3_TR32192_c1_g6_i2.rpkm.png]

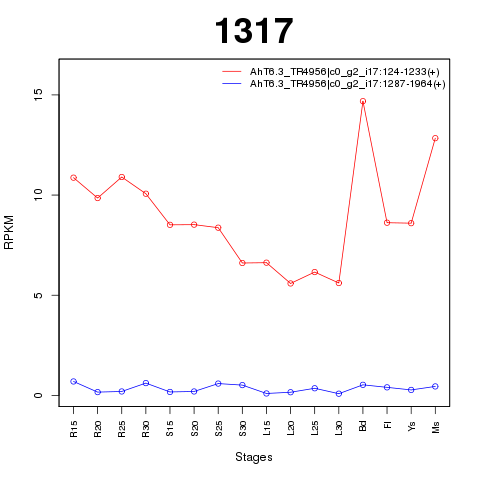

Supplement: S4 Dataset — (ZIP) [file pone.0180528.s009.zip › chimeras_581_PNGs/1317.AhT6.3_TR4956_c0_g2_i17.rpkm.png]

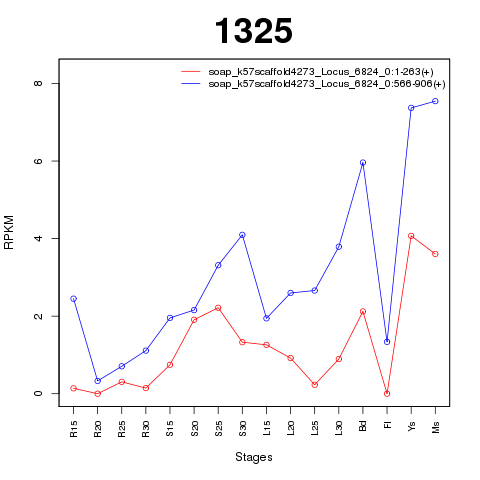

Supplement: S4 Dataset — (ZIP) [file pone.0180528.s009.zip › chimeras_581_PNGs/1325.soap_k57scaffold4273_Locus_6824_0.rpkm.png]

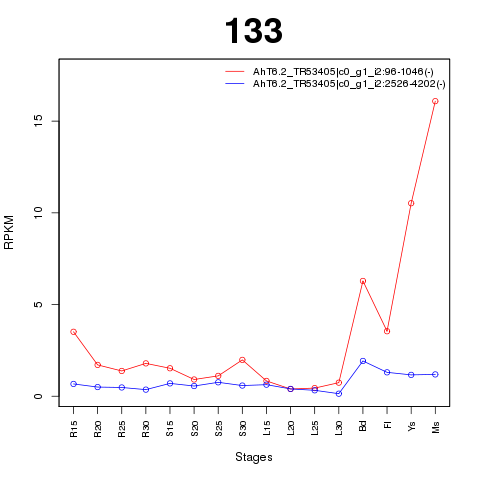

Supplement: S4 Dataset — (ZIP) [file pone.0180528.s009.zip › chimeras_581_PNGs/133.AhT6.2_TR53405_c0_g1_i2.rpkm.png]

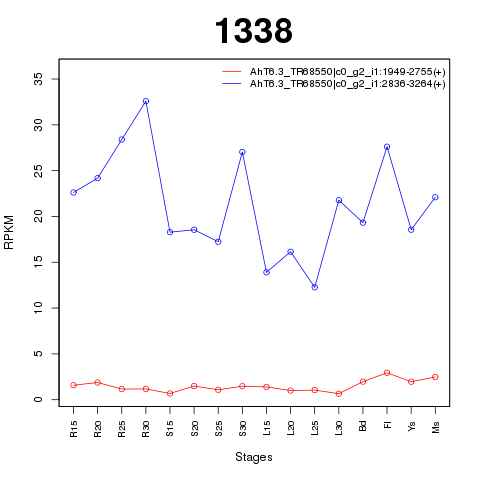

Supplement: S4 Dataset — (ZIP) [file pone.0180528.s009.zip › chimeras_581_PNGs/1338.AhT6.3_TR68550_c0_g2_i1.rpkm.png]

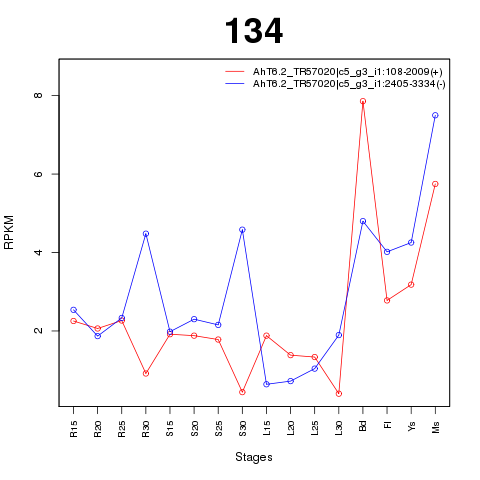

Supplement: S4 Dataset — (ZIP) [file pone.0180528.s009.zip › chimeras_581_PNGs/134.AhT6.2_TR57020_c5_g3_i1.rpkm.png]

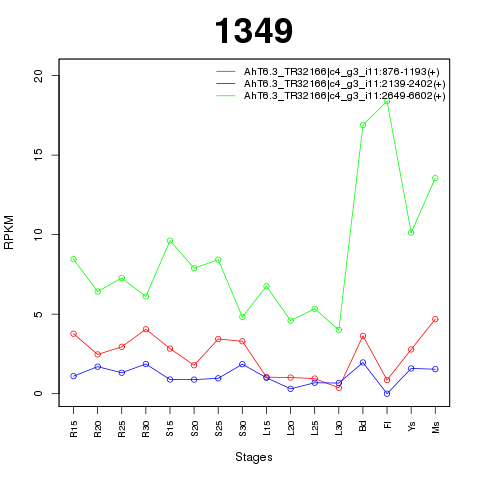

Supplement: S4 Dataset — (ZIP) [file pone.0180528.s009.zip › chimeras_581_PNGs/1349.AhT6.3_TR32166_c4_g3_i11.rpkm.png]

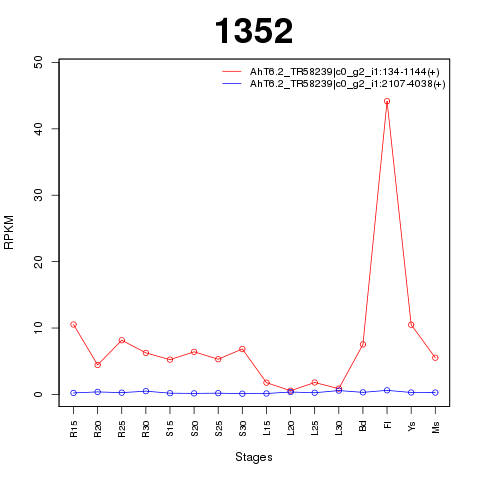

Supplement: S4 Dataset — (ZIP) [file pone.0180528.s009.zip › chimeras_581_PNGs/1352.AhT6.2_TR58239_c0_g2_i1.rpkm.png]

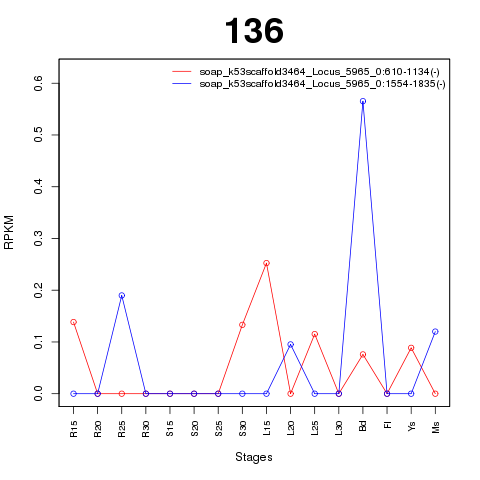

Supplement: S4 Dataset — (ZIP) [file pone.0180528.s009.zip › chimeras_581_PNGs/136.soap_k53scaffold3464_Locus_5965_0.rpkm.png]

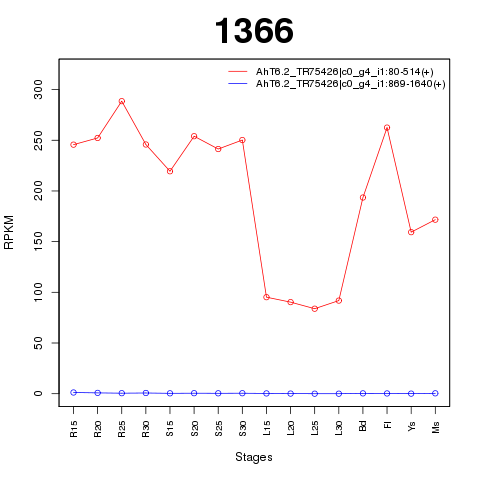

Supplement: S4 Dataset — (ZIP) [file pone.0180528.s009.zip › chimeras_581_PNGs/1366.AhT6.2_TR75426_c0_g4_i1.rpkm.png]

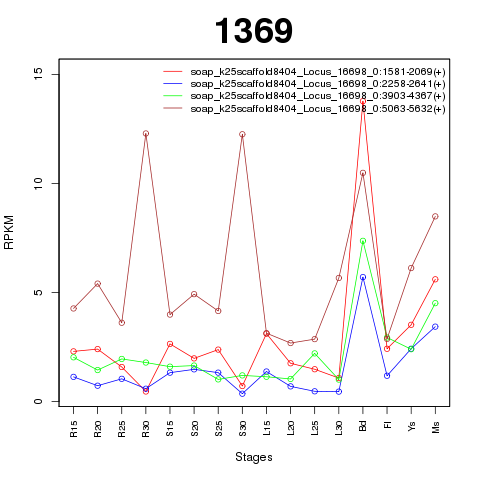

Supplement: S4 Dataset — (ZIP) [file pone.0180528.s009.zip › chimeras_581_PNGs/1369.soap_k25scaffold8404_Locus_16698_0.rpkm.png]

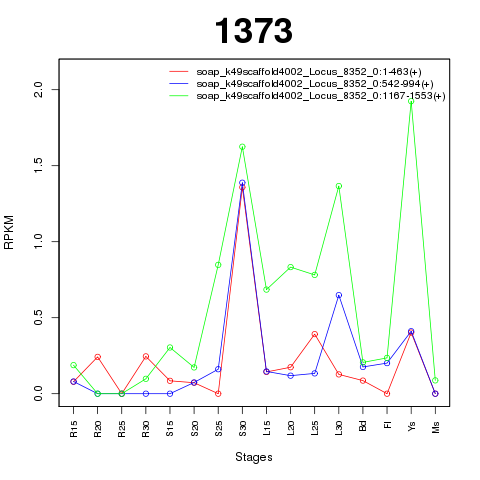

Supplement: S4 Dataset — (ZIP) [file pone.0180528.s009.zip › chimeras_581_PNGs/1373.soap_k49scaffold4002_Locus_8352_0.rpkm.png]

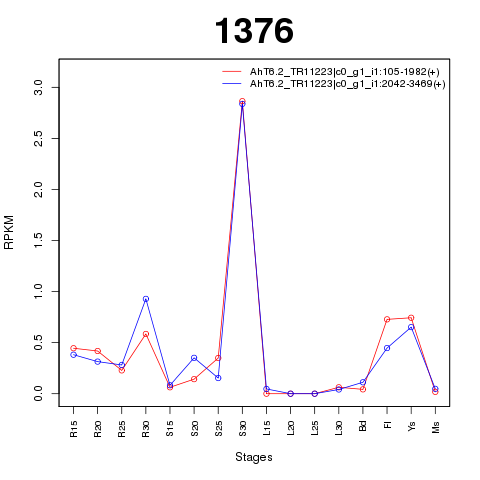

Supplement: S4 Dataset — (ZIP) [file pone.0180528.s009.zip › chimeras_581_PNGs/1376.AhT6.2_TR11223_c0_g1_i1.rpkm.png]

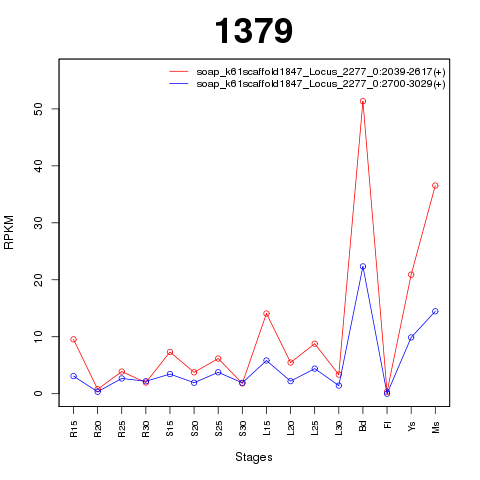

Supplement: S4 Dataset — (ZIP) [file pone.0180528.s009.zip › chimeras_581_PNGs/1379.soap_k61scaffold1847_Locus_2277_0.rpkm.png]

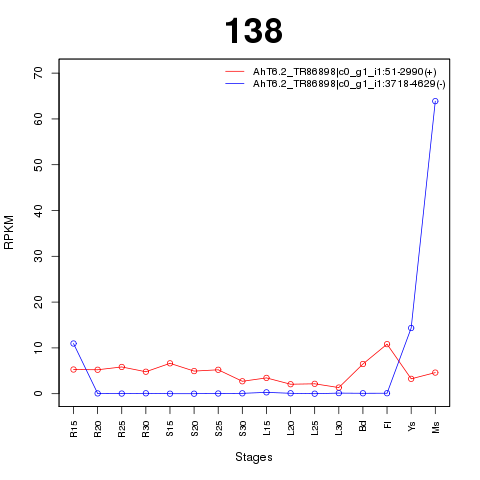

Supplement: S4 Dataset — (ZIP) [file pone.0180528.s009.zip › chimeras_581_PNGs/138.AhT6.2_TR86898_c0_g1_i1.rpkm.png]

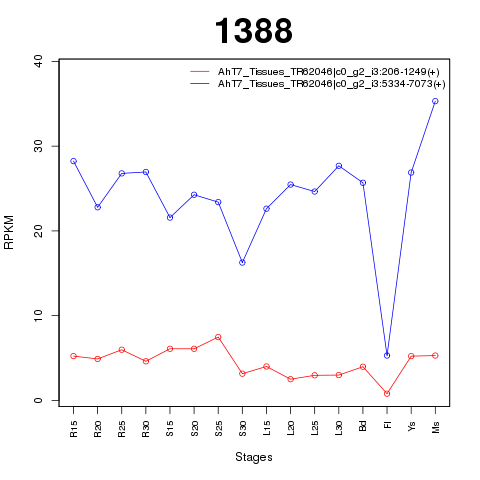

Supplement: S4 Dataset — (ZIP) [file pone.0180528.s009.zip › chimeras_581_PNGs/1388.AhT7_Tissues_TR62046_c0_g2_i3.rpkm.png]

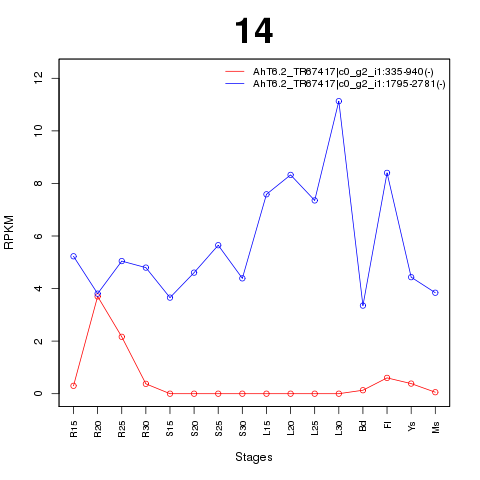

Supplement: S4 Dataset — (ZIP) [file pone.0180528.s009.zip › chimeras_581_PNGs/14.AhT6.2_TR67417_c0_g2_i1.rpkm.png]

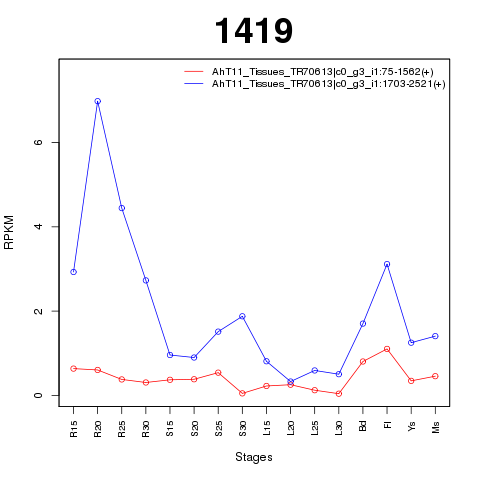

Supplement: S4 Dataset — (ZIP) [file pone.0180528.s009.zip › chimeras_581_PNGs/1419.AhT11_Tissues_TR70613_c0_g3_i1.rpkm.png]

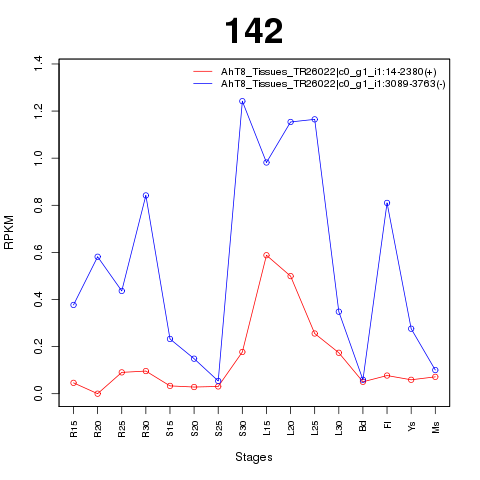

Supplement: S4 Dataset — (ZIP) [file pone.0180528.s009.zip › chimeras_581_PNGs/142.AhT8_Tissues_TR26022_c0_g1_i1.rpkm.png]

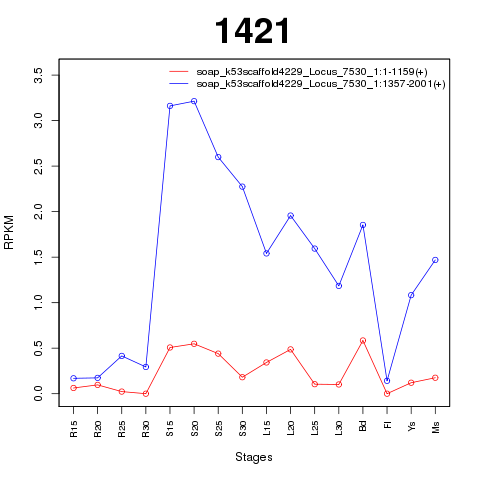

Supplement: S4 Dataset — (ZIP) [file pone.0180528.s009.zip › chimeras_581_PNGs/1421.soap_k53scaffold4229_Locus_7530_1.rpkm.png]

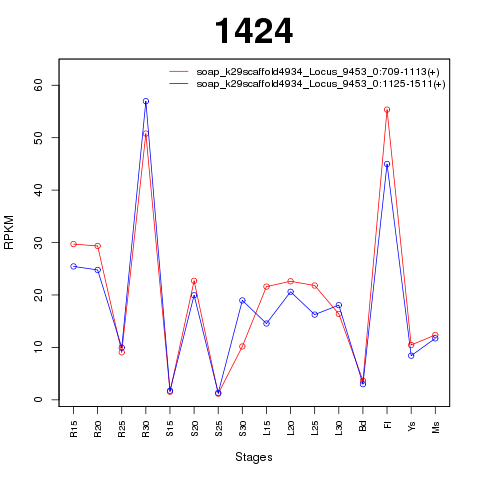

Supplement: S4 Dataset — (ZIP) [file pone.0180528.s009.zip › chimeras_581_PNGs/1424.soap_k29scaffold4934_Locus_9453_0.rpkm.png]

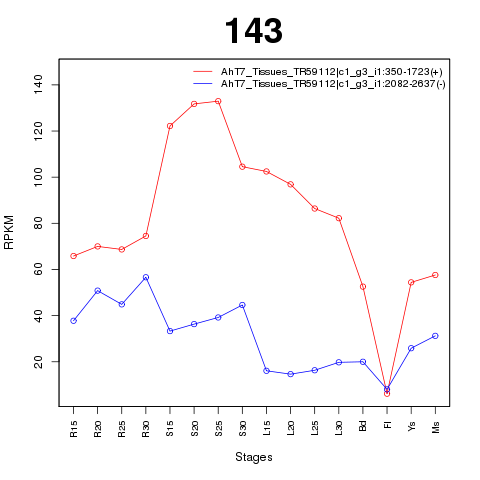

Supplement: S4 Dataset — (ZIP) [file pone.0180528.s009.zip › chimeras_581_PNGs/143.AhT7_Tissues_TR59112_c1_g3_i1.rpkm.png]

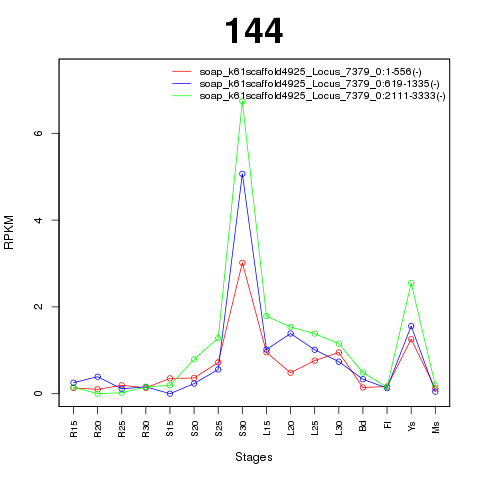

Supplement: S4 Dataset — (ZIP) [file pone.0180528.s009.zip › chimeras_581_PNGs/144.soap_k61scaffold4925_Locus_7379_0.rpkm.png]

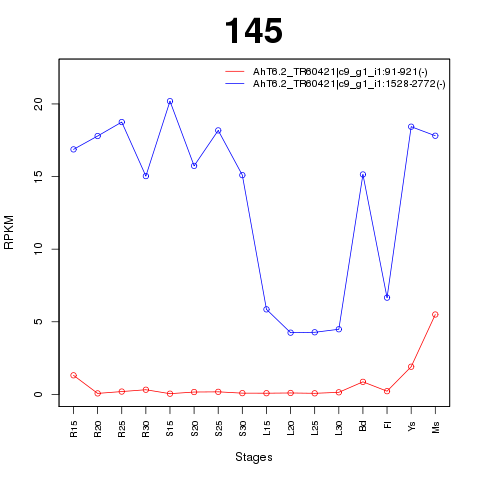

Supplement: S4 Dataset — (ZIP) [file pone.0180528.s009.zip › chimeras_581_PNGs/145.AhT6.2_TR60421_c9_g1_i1.rpkm.png]

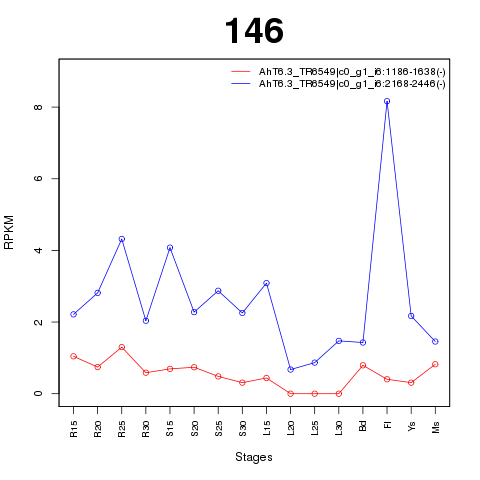

Supplement: S4 Dataset — (ZIP) [file pone.0180528.s009.zip › chimeras_581_PNGs/146.AhT6.3_TR6549_c0_g1_i6.rpkm.png]

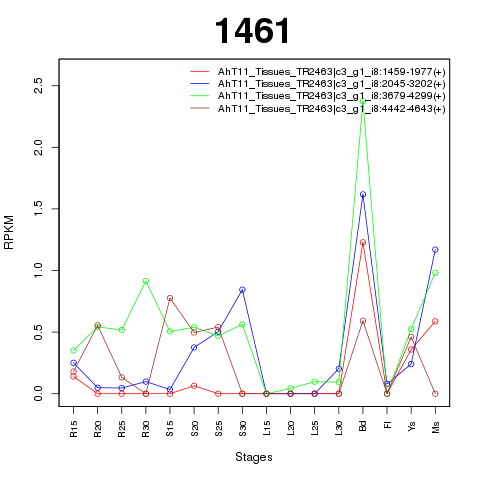

Supplement: S4 Dataset — (ZIP) [file pone.0180528.s009.zip › chimeras_581_PNGs/1461.AhT11_Tissues_TR2463_c3_g1_i8.rpkm.png]

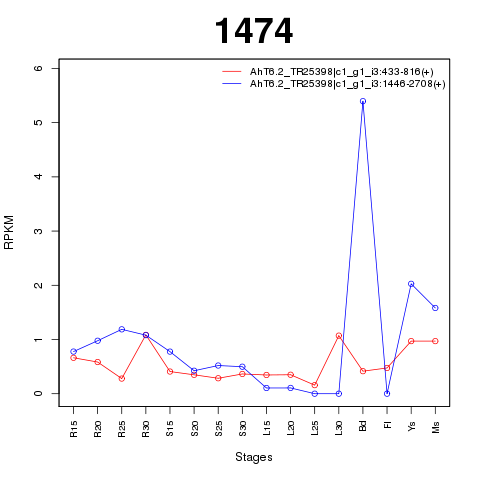

Supplement: S4 Dataset — (ZIP) [file pone.0180528.s009.zip › chimeras_581_PNGs/1474.AhT6.2_TR25398_c1_g1_i3.rpkm.png]

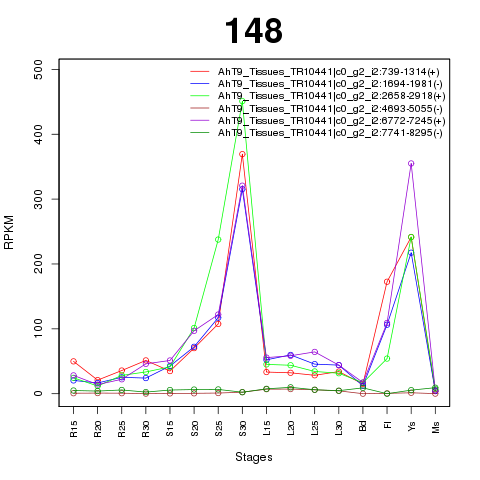

Supplement: S4 Dataset — (ZIP) [file pone.0180528.s009.zip › chimeras_581_PNGs/148.AhT9_Tissues_TR10441_c0_g2_i2.rpkm.png]

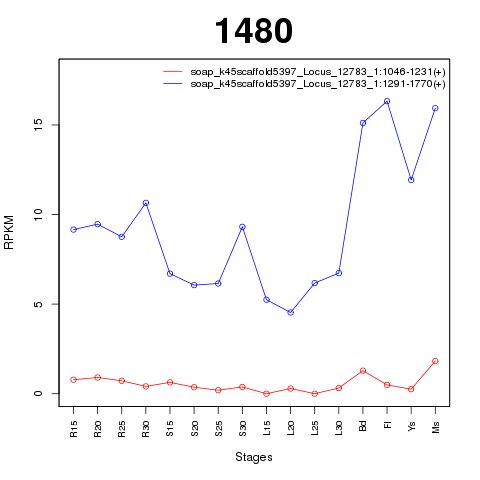

Supplement: S4 Dataset — (ZIP) [file pone.0180528.s009.zip › chimeras_581_PNGs/1480.soap_k45scaffold5397_Locus_12783_1.rpkm.png]

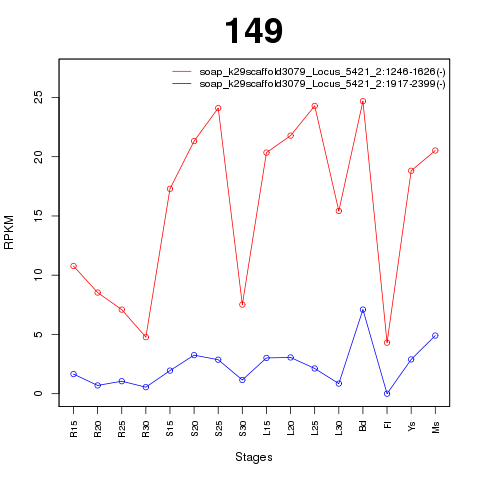

Supplement: S4 Dataset — (ZIP) [file pone.0180528.s009.zip › chimeras_581_PNGs/149.soap_k29scaffold3079_Locus_5421_2.rpkm.png]

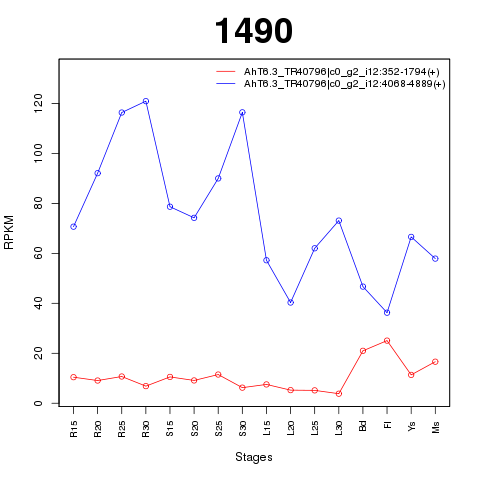

Supplement: S4 Dataset — (ZIP) [file pone.0180528.s009.zip › chimeras_581_PNGs/1490.AhT6.3_TR40796_c0_g2_i12.rpkm.png]

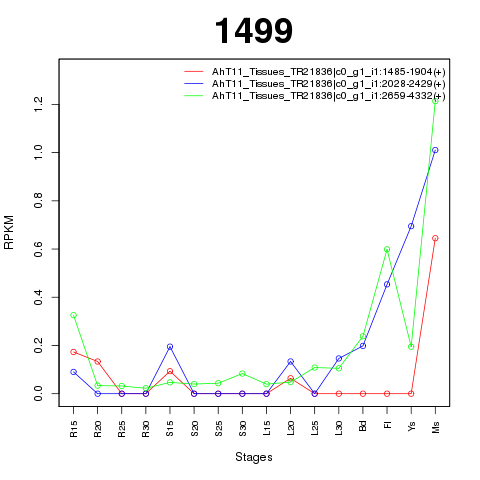

Supplement: S4 Dataset — (ZIP) [file pone.0180528.s009.zip › chimeras_581_PNGs/1499.AhT11_Tissues_TR21836_c0_g1_i1.rpkm.png]

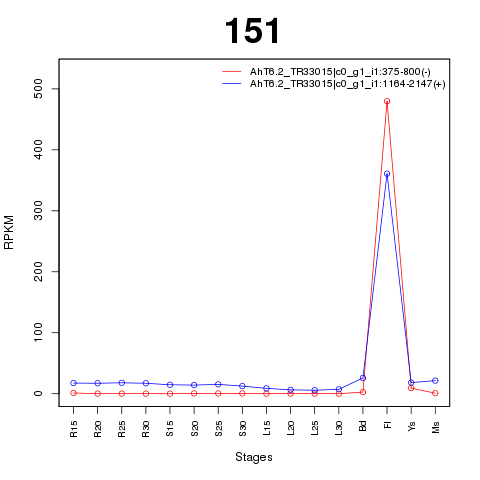

Supplement: S4 Dataset — (ZIP) [file pone.0180528.s009.zip › chimeras_581_PNGs/151.AhT6.2_TR33015_c0_g1_i1.rpkm.png]

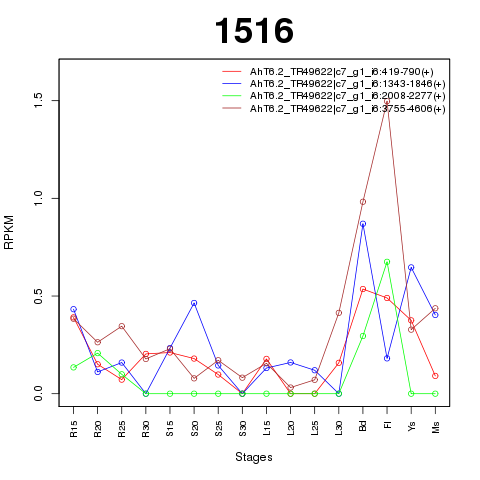

Supplement: S4 Dataset — (ZIP) [file pone.0180528.s009.zip › chimeras_581_PNGs/1516.AhT6.2_TR49622_c7_g1_i6.rpkm.png]

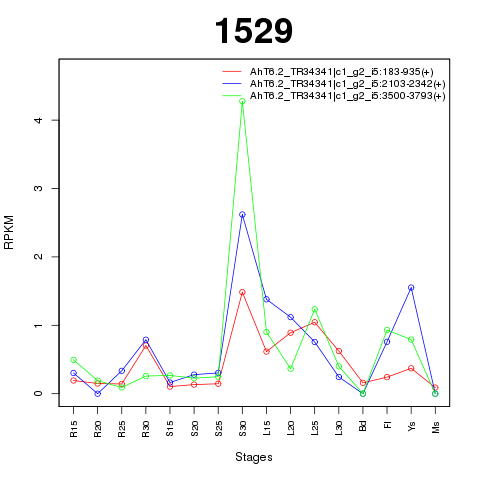

Supplement: S4 Dataset — (ZIP) [file pone.0180528.s009.zip › chimeras_581_PNGs/1529.AhT6.2_TR34341_c1_g2_i5.rpkm.png]

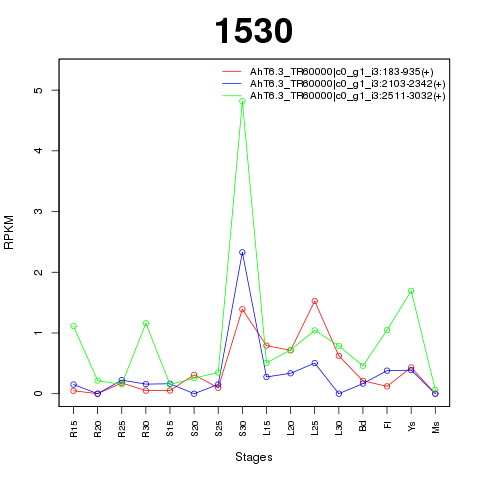

Supplement: S4 Dataset — (ZIP) [file pone.0180528.s009.zip › chimeras_581_PNGs/1530.AhT6.3_TR60000_c0_g1_i3.rpkm.png]

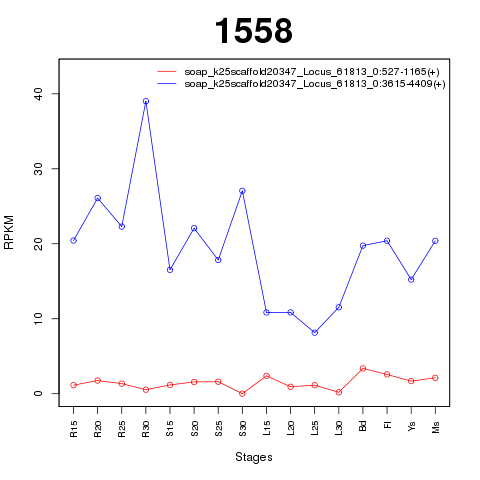

Supplement: S4 Dataset — (ZIP) [file pone.0180528.s009.zip › chimeras_581_PNGs/1558.soap_k25scaffold20347_Locus_61813_0.rpkm.png]

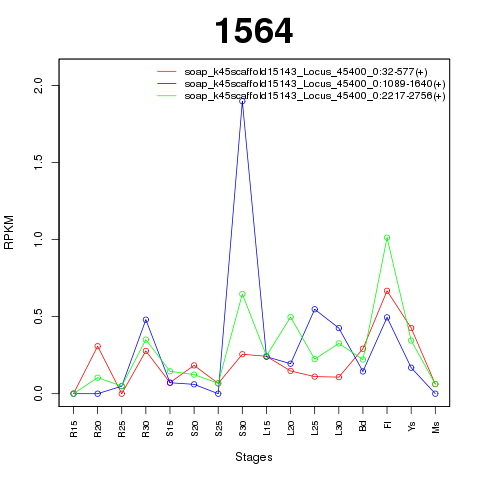

Supplement: S4 Dataset — (ZIP) [file pone.0180528.s009.zip › chimeras_581_PNGs/1564.soap_k45scaffold15143_Locus_45400_0.rpkm.png]

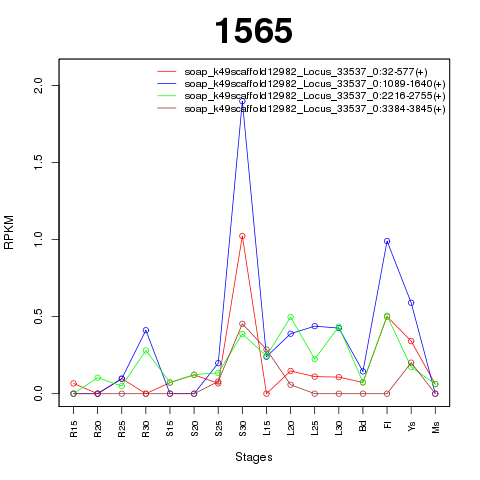

Supplement: S4 Dataset — (ZIP) [file pone.0180528.s009.zip › chimeras_581_PNGs/1565.soap_k49scaffold12982_Locus_33537_0.rpkm.png]

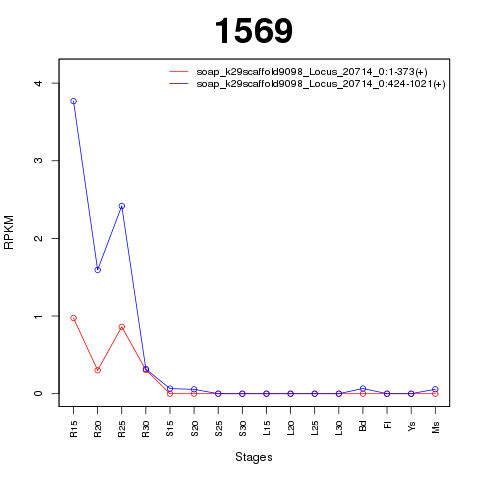

Supplement: S4 Dataset — (ZIP) [file pone.0180528.s009.zip › chimeras_581_PNGs/1569.soap_k29scaffold9098_Locus_20714_0.rpkm.png]

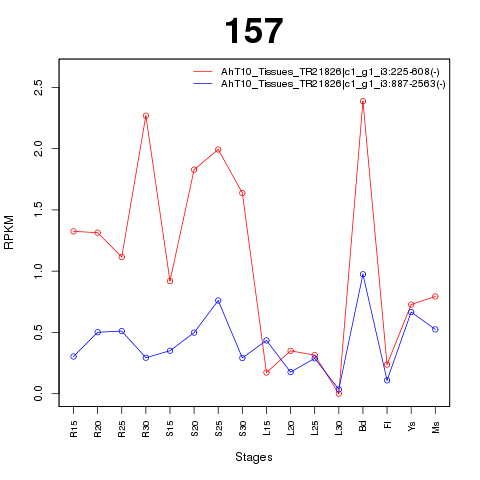

Supplement: S4 Dataset — (ZIP) [file pone.0180528.s009.zip › chimeras_581_PNGs/157.AhT10_Tissues_TR21826_c1_g1_i3.rpkm.png]

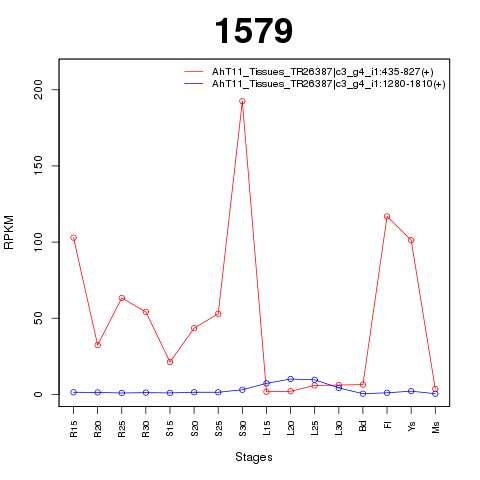

Supplement: S4 Dataset — (ZIP) [file pone.0180528.s009.zip › chimeras_581_PNGs/1579.AhT11_Tissues_TR26387_c3_g4_i1.rpkm.png]

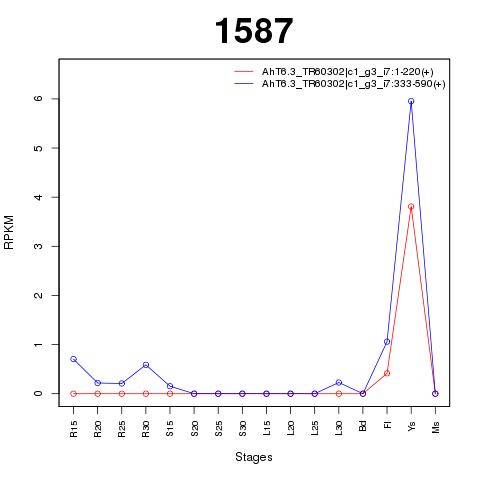

Supplement: S4 Dataset — (ZIP) [file pone.0180528.s009.zip › chimeras_581_PNGs/1587.AhT6.3_TR60302_c1_g3_i7.rpkm.png]

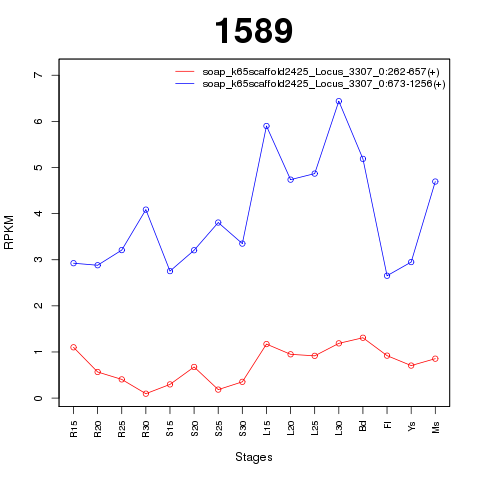

Supplement: S4 Dataset — (ZIP) [file pone.0180528.s009.zip › chimeras_581_PNGs/1589.soap_k65scaffold2425_Locus_3307_0.rpkm.png]

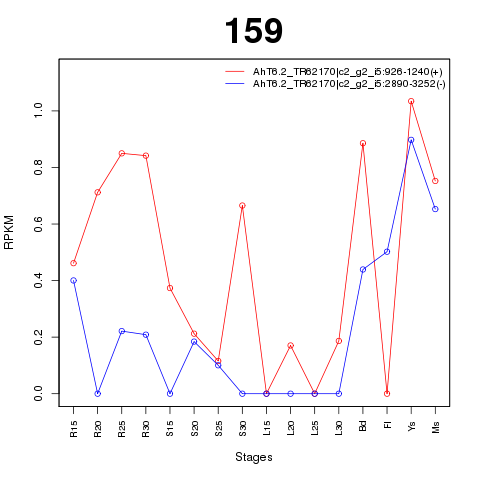

Supplement: S4 Dataset — (ZIP) [file pone.0180528.s009.zip › chimeras_581_PNGs/159.AhT6.2_TR62170_c2_g2_i5.rpkm.png]

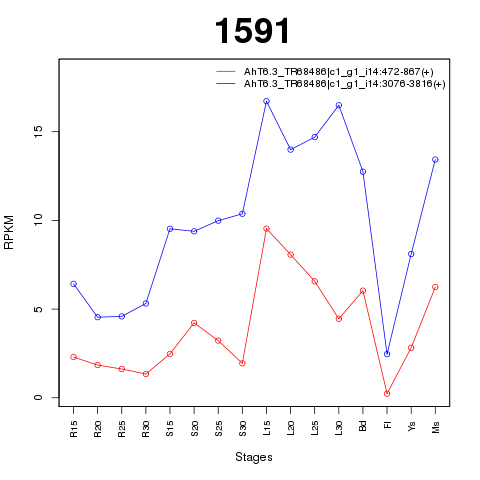

Supplement: S4 Dataset — (ZIP) [file pone.0180528.s009.zip › chimeras_581_PNGs/1591.AhT6.3_TR68486_c1_g1_i14.rpkm.png]

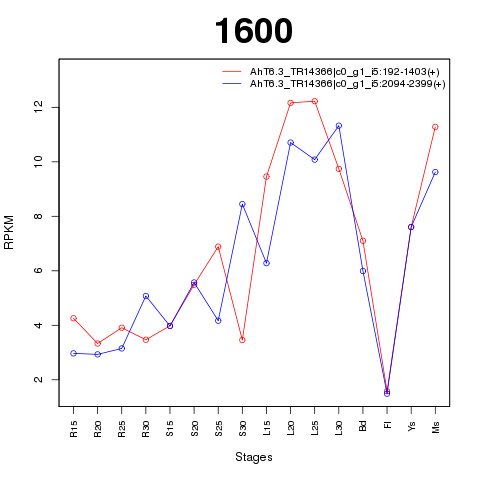

Supplement: S4 Dataset — (ZIP) [file pone.0180528.s009.zip › chimeras_581_PNGs/1600.AhT6.3_TR14366_c0_g1_i5.rpkm.png]

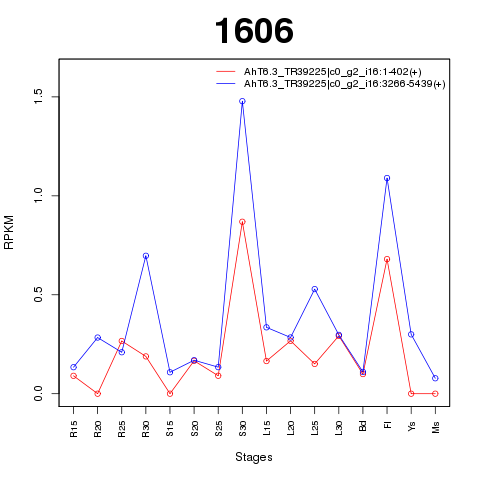

Supplement: S4 Dataset — (ZIP) [file pone.0180528.s009.zip › chimeras_581_PNGs/1606.AhT6.3_TR39225_c0_g2_i16.rpkm.png]

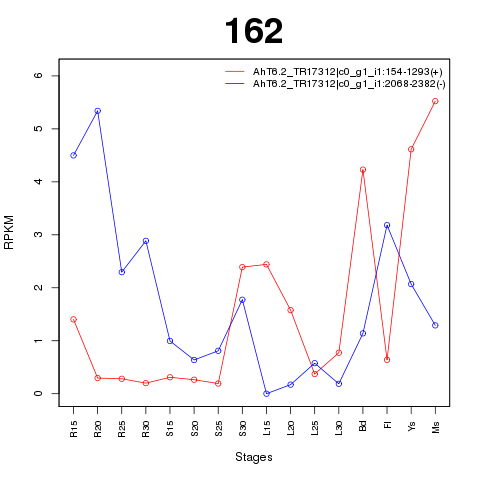

Supplement: S4 Dataset — (ZIP) [file pone.0180528.s009.zip › chimeras_581_PNGs/162.AhT6.2_TR17312_c0_g1_i1.rpkm.png]
